# Characterization of new psychoactive substances by integrating benchtop NMR to multi‐technique databases

**DOI:** 10.1002/dta.3332

**Published:** 2022-06-26

**Authors:** Thomas Castaing‐Cordier, Alejandra Benavides Restrepo, Damien Dubois, Virginie Ladroue, Fabrice Besacier, Audrey Buleté, Céline Charvoz, Anais Goupille, Denis Jacquemin, Patrick Giraudeau, Jonathan Farjon

**Affiliations:** ^1^ Nantes Université, CNRS, CEISAM, UMR 6230 Nantes France; ^2^ Laboratoire de Police Scientifique de Lyon Service National de Police Scientifique Ecully France; ^3^ Sous‐direction de la stratégie de l'innovation et du pilotage Service National de Police Scientifique Ecully France

**Keywords:** benchtop, database, IR, NMR, NPS

## Abstract

New psychoactive substances (NPS) have become a serious threat for public health due to their ability to be sold in the street or on internet. NPS are either derived from commercial drugs which are misused (recreational rather than medical use) or whose structure is slightly modified. To regulate NPS, it is essential to accurately characterize them, either to recognize molecules that were previously identified or to quickly elucidate the structure of unknown ones. Most approaches rely on the determination of the exact mass obtained by high‐resolution mass spectrometry requiring expensive equipment. This motivated us to develop a workflow in which the elucidation is assisted with databases and does not need the exact mass. This workflow combines 1D and 2D NMR measurements performed on a benchtop spectrometer with IR spectroscopy, for creating a multi‐technique database to characterize pure and mixed NPS. The experimental database was created with 57 entries mostly coming from seizures, mainly cathinones, cannabinoids, amphetamines, arylcyclohexylamines, and fentanyl. A blind validation of the workflow was carried out on a set of six unknown seizures. In the first three cases, AF, AB‐FUBINACA, and a mixture of 2C‐I and 2C‐E could be straightforwardly identified with the help of their reference spectra in the database. The two next samples were elucidated for the first time with the help of the database to reveal NEK and MPHP substances. Finally, a precise quantification of each characterized NPS was obtained in order to track NPS trafficking networks.

## Graphical abstract

New psychoactive substances (NPS) have become a serious threat for public health. A new workflow was developed for NPS characterization even in mixtures; it combines 1D and 2D NMR performed on a benchtop spectrometer with IR spectroscopy for creating a multi‐technique database (57 entries). The latter was blind validated on a set of six unknown seizures, and a precise quantification of each characterized NPS was obtained in order to track NPS trafficking networks.

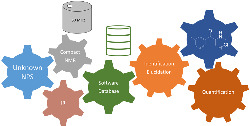

## 1 INTRODUCTION

New psychoactive substances (NPS)^1^ encompass several families of molecules mimicking the effects of different conventional illicit products, for example, ecstasy, cocaine, or cannabis. NPS are either derived from commercial drugs which have been misused (recreational rather than medical use) or whose structure has been slightly modified. As a consequence of such chemical modifications, these hazardous compounds are not always controlled under the International Drug Control Conventions, and their legal status often remains undefined. At the end of 2020, the European Monitoring Center of Drugs and Drugs Addiction (EMCDDA) followed approximately 800 NPS from different families.^2^, ^3^ These NPS include synthetic cannabinoids derived from marijuana, ketamine‐like compounds which are used as anesthetics, fentanyloids which is a painkiller more powerful than morphine, and benzodiazepines that are used for curing anxiety, cathinones, and phenethylamines. The effects of those substances generally remain unknown and their consumption can lead to intoxication, overdose, or even death. Due to the growing consumption of NPS on the market as well as the difficulty to constantly adapt the regulation of European members, there is an urgent need for accessible and reliable analytical tools allowing improved tracking of NPS seized by the Police and/or purchased on the internet. Indeed, it is essential to accurately characterize NPS, to recognize molecules that were previously identified, and to elucidate the structure of unknown ones. Identification of known substances consists in comparing experimental data of a seized compound with reference data, which is quite straightforward. In contrast, elucidation is a more challenging task, necessary when identification fails and consisting in determining the structure of the unknowns in a sample from a set of combined analytical data. An efficient NMR‐based approach named CASE (computer‐assisted spectral elucidation) has been developed for structure elucidation of small molecules.^4^ CASE uses software to generate all possible molecular structures that are consistent with a particular set of 1D and 2D NMR data. However, CASE relies on the determination of the exact mass obtained by high‐resolution mass spectrometry (HRMS), a technique which is not widely available in forensic science services. This motivated us to develop a workflow in which the elucidation is assisted with databases and does not require prior knowledge of the exact mass.

**FIGURE 1:**
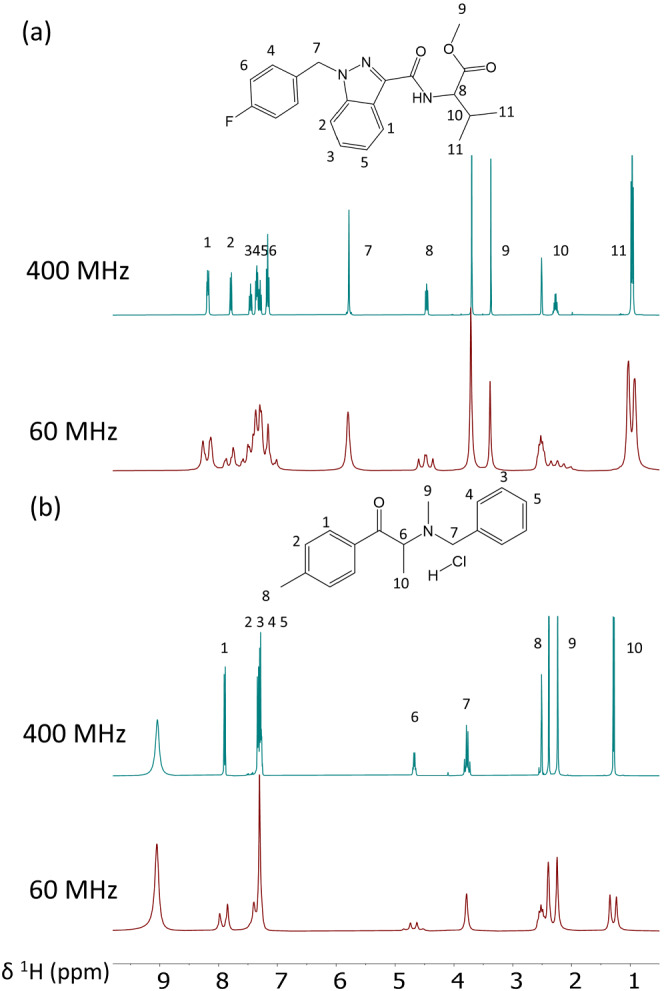
Spectrum of (a) 370mM AMB‐FUBINACA in DMSO‐d6 and (b) 306mM 2‐BMMP in DMSO‐d6 acquired at high‐field (top: 400 MHz) and on a benchtop spectrometer (bottom: 60 MHz). Peak assignment after phasing and baseline correction is indicated. The two spectra are obtained with the same conditions: eight scans, a repetition time (TR) of 30 s, and an acquisition time of 1.6 s at 299.6 K. Most of the signals are overlapped at 60 MHz especially for the aromatic area of AMB‐FUBINACA. This illustrates the resolution limitation of benchtop NMR. Indeed, since the resolution scales linearly with the magnetic field B0 in theory, the 400 MHz spectrum is expected to be roughly seven times more resolved than at 60 MHz. As regards sensitivity, it scales with B0^3/2^; hence, the 400 MHz spectrum is expected to be approximately 17 times more sensitive than a 60 MHz spectrum recorded in identical conditions [Colour figure can be viewed at wileyonlinelibrary.com]

NPS characterization can be achieved by a variety of complementary analytical techniques. The first one is gas chromatography coupled to mass spectrometry (GC–MS), currently the most used in forensics.^5^ MS combined with chemical ionization (CI) may be used to provide the molecular weight, but a relatively expensive HRMS instrumentation is required to access accurate molecular weight. MS combined with electron ionization (EI) mode provides information on different fragments of the molecule, and hyphenated MS/MS (or MS^n^) delivers further insights into the fragmentation schemes of the molecule. Another important technique is infrared (IR) spectroscopy which is mainly used to determine chemical functions within a molecule, but can also be applied for spectral recognition.^6^, ^7^, ^8^ Unfortunately, these methods provide limited structural information. NMR spectroscopy is probably the most powerful structure identification and elucidation tool for small molecules such as NPS. Indeed, it offers highly accurate and specific information on the chemical environment of all atoms through chemical shifts, while providing crucial input on atomic connectivities through J‐couplings. Moreover, highly accurate quantification is achievable with NMR with limited sample preparation and a short acquisition time for 1D experiments. However, high‐field NMR (^1^H frequency > 300 MHz) is rarely used in forensics due to the high purchase, maintenance, and running costs in addition to the need for dedicated staff and to the bulkiness of the NMR instrument. However, during the last decade, new compact NMR apparatus emerged, with magnetic fields between 1 and 2.1 T. These instruments can be installed on a benchtop, are easily transportable, and do not require maintenance (no cryogenic fluids). Nevertheless, benchtop NMR remains a young and underexplored technique for NPS characterization as compared with conventional high‐field NMR.^9^, ^10^ Also, benchtop NMR has to face intrinsic sensitivity and resolution limits, which are illustrated in Figure 1 in the case of two common NPS.

Such limitations make it difficult to only rely on benchtop NMR for structure identification or elucidation. Therefore, combinations with other methods like GC–MS or IR and/or with databases become a prerequisite for a reliable, fast, and automated characterization. Two such multi‐method approaches were already explored in a forensic framework^11^ In a first study merging NMR and GC–MS, ^1^H NMR spectra of reference compounds were collected using an 80 MHz instrument to create a reference library of 302 spectra of different NPS classes.^12^ Next 432 seized samples were analyzed by NMR and GC–MS for cross‐validation. ^1^H NMR analysis nicely matched the GC–MS results with a 93% consistency rate. Another study reported the identification of drugs in two case samples, using a limited library of 12 spectra on an 80 MHz benchtop spectrometer, visually compared with data collected on a 600 MHz spectrometer.^13^ In agreement with GC–MS analysis, the seized samples were found to contain morphine, acetyl codeine, MAM, and MDMA. In spite of these encouraging preliminary results, there is no general workflow incorporating benchtop NMR for the identification and elucidation of NPS structures. A first reason is that the above‐mentioned examples were limited to 1D ^1^H NMR spectroscopy and did not include 2D NMR experiments, which should provide a much higher degree of confidence in terms of structure characterization. Moreover, the reported elucidation procedures rely on the determination of the exact mass as an initial step, but as explained above, HRMS instruments are uncommon in forensic laboratories. It is also important to note that the SWDRUGS group recommends using at least two techniques for unambiguous identification, which motivated us to combine benchtop NMR with another analytical approach.^14^

Databases can be used with other analytical techniques such as IR. For instance, in a study by Jones et al., 221 samples—most of them in mixtures—were screened using IR and Raman.^15^ Authors compared the spectra to the database, and if a reference matched, they subtracted the reference spectra to the unknown one and made another comparison with the database. Only 41% of samples were unambiguously identified. Other samples were sent to MS and NMR, for a full elucidation procedure. With this approach, 33 samples were identified and added to the IR and Raman library.

About NMR quantification, some previous works were carried out on benchtop NMR spectra. Naqi et al. quantified seized MDMA by using ^1^H‐qNMR, UHPLC, and UHPLC–MS.^16^ Maleic acid and MDMA‐d_5_ were used, respectively, as an internal reference for NMR and UHPLC measurements. The MDMA concentrations determined by UHPLC and NMR were found comparable with no significant statistical difference as revealed by ANOVA single factor analysis. Quantitative NMR with benchtop NMR was applied to drugs with similar molecular structures. For example, Hussain et al. were able to quantify the mass weight of MDMA contained in a tablet.^17^ The values obtained on eight tablets were between 209 and 212 mg and are comparable with results given by GC–MS.

In this context, this work aims to develop and evaluate a general workflow based on optimized 1D and 2D NMR measurements performed on a benchtop spectrometer, combined with IR spectroscopy, for creating a multi‐technique database allowing the characterization of pure and mixed NPS. Both identification and elucidation are considered in this workflow, which uses accessible analytical techniques while keeping in mind the demand of end‐users in terms of reliability, robustness, and experiment time. After describing its implementation and the choice of the key parameters, the workflow was blind‐tested on six real‐seized samples, and the quantification capabilities of benchtop NMR were also assessed.

## 2 MATERIALS AND METHODS

### 2.1 Sample preparation

Fifty‐seven samples were used in this study. Five of them were purchased from Lipomed, seven samples were seized by the Finnish police, while all the others came from seizures by the French police. Also, six unknown seizures were obtained from the French police to allow validating the database. DMSO‐d_6_ purchased from Eurisotop with a purity of 99.8% was used as an NMR solvent. TMS from Acros Organics with a purity of 99.9% and TSP from Eurisotop with a purity of 99.8% were used as chemical shift and concentration references, respectively. All seizures were dissolved in DMSO‐d_6_ to obtain a concentration as close as possible to 300 mM (see Table S1), and 10 μl of TMS was added to calibrate ^1^H and ^13^C chemical shifts.

### 2.2 NMR

All the spectra were recorded at 26.5°C using a ^1^H, ^19^F, ^13^C 60 MHz Spinsolve spectrometer from Magritek equipped with a 20‐position autosampler and a gradient coil in the direction of the static magnetic field B_0_. The duration of 90° hard pulses was 14 μs at 0 dBW for ^1^H, 60 μs at 0 dBW for ^19^F, and 65.9 μs at −14 dBW for ^13^C. All acquisitions were made with the SpinsolveExpert software (v.1.41.07). Moreover, an Avance III HD 700 MHz spectrometer with a ^1^H/^13^C/^15^N cryoprobe from Bruker was used to confirm the identification/elucidation of the unknown NPS in samples.

Three NMR pulse sequences were chosen for our study, 1D ^1^H, 1D ^19^F, and 2D ^1^H‐^13^C heteronuclear single quantum coherence (HSQC). The HSQC sequence was improved by optimization with the help of a test molecule, named 2‐FDCK (see Table S1). Three variants of the HSQC sequence were tested, namely, Echo‐Antiecho (EA) HSQC with WALTZ decoupling, phase sensitive (PH) HSQC with WALTZ decoupling, PH HSQC with Multiplicity Edition (ME), and WALTZ decoupling. PH HSQC was found in average twice more sensitive than EA HSQC (see S2). The PH HSQC ME (see S3) was found to be the most informative since it allowed determining the multiplicity *n* of ^13^C(^1^H)*n* but this pulse sequence was 20% less sensitive than PH HSQC. The most important acquisitions parameters are gathered in Table 1.

**TABLE 1:** NMR acquisitions parameters

Parameters	1D ^1^H	1D ^19^F	2D ^1^H‐^13^C HSQC
NS	8	360	24
DW (μs)	200	50	100
AQ (ms)	1638.4	204.8	409.6
TR (s)	30	1	2
NI	—	—	128
T_exp_ (min)	4	6	103

*Note*: NS is the number of scans, DW is the dwell time, AQ is the AcQuisition time, TR is the repetition time (including the acquisition time and the recovery delay), and NI is the number of increments in the indirect dimension of 2D HSQC spectra. T_exp_ indicates the experiment time.

The NMR processing was performed with MestreNova™ software (version 14.1). The 1D free induction decays were first multiplied by an exponential apodization function (0.3 Hz line broadening) and zero‐filled to a factor 4 then manually phased and baseline corrected with a Whittaker smoother algorithm. The 2D free induction decays were first multiplied by a cosine apodization function and zero‐filled to a factor 4 in both dimensions, then manually phased and baseline corrected with Whittaker smoother algorithm. ^1^H and ^13^C chemical shifts were referenced to TMS, and ^19^F chemical shifts were referenced to the internal lock substance of the spectrometer.

### 2.3 IR

Analyses were carried out with a Bruker tensor 27 spectrometer in Attenuated Total Reflectance (ATR) mode. Each spectrum was acquired with 32 scans with a 400—4000 cm^−1^ spectral range and a 2 cm^−1^ spectral resolution. The processing of the spectra was achieved with the Opus™ (v8.5) software. CO_2_, H_2_O compensation, baseline, and smoothing were applied to all IR spectra to obtain more reliable NPS IR fingerprints.

### 2.4 ACD/Labs software

All analytical data were gathered in the ACD/Labs software (version 2020.1.0 of July 15, 2020) from Advanced Chemistry Development, Inc (ACD/Labs). First, a project with all NMR experiments and the dedicated structure was created for each molecule and added to the database after peak picking and assignment of all signals and correlations. Then the corresponding IR spectrum was added to the database after an automatic peak picking.

### 2.5 Database parameters

Once the database has been created, two search methods are available in the ACD/Labs software to compare an unknown spectrum with one from the database. The first one, denoted “similarity search”, compares the shape of the spectra only, disregarding peak picking and the multiplicity of the signals. The second one, called “peak searching”, compares the peak picking of the NMR signal, an approach which is, obviously, significantly operator‐dependent. With both search methods, the comparison between the experimental spectrum and the database yields a result called the Hit Quality Index (HQI) which quantifies the agreement between the database spectrums the most closely matching and the spectrum of the unknown compound.

In the “similarity search” method, HQI can be calculated with two algorithms detailed below. In both cases, the query spectrum is first indexed and split into *N* small regions. Next, all regions are integrated and, depending on the calculated integral, an index in the [−127;+127] range is assigned to each region.

The first algorithm relies on the calculation of the Euclidian difference and treats each spectrum as a vector in a *N*‐dimensional space. Thus, the resulting HQI is a measurement of the angle between the vectors giving a value between 0 (best match) and the square root of 2 (worst match). In the ACD/Labs software, this value is next scaled to deliver a value between 0 (worst match) and 100 (best match) for the HQI display:

(1) $$\mathrm{HQI}=\frac{1}{N\times 127}\sqrt{\sum_{i=1}^{L}\mathrm{abs}{\left(p_{i}^{\mathrm{EXP}}-p_{i}^{\mathrm{DB}}\right)}^{2}},$$

with *L* the compared regions, *N* the number of used indexes, and $p_{i}$ the index from the experimental (EXP) spectrum and the database spectrum (DB).

The second algorithm relies on the calculation of the absolute distance based on the difference for each data point with respect to the total area under the curve (i.e., the sum of all the intensities). In other words, each data point represents a percentage of the total area. Thus, the larger the intensity value of a data point, the larger its weight:

(2) $$\mathrm{HQI}=\frac{1}{N\times 127}\sum_{i=1}^{L}|\left(p_{i}^{\mathrm{EXP}}-p_{i}^{\mathrm{DB}}\right)|.$$

Only one HQI algorithm is available for “peak searching” calculated as follows:

(3) $$\mathrm{HQI}=\frac{1}{L}\sum_{k=1}^{L}\frac{N_{k}}{M_{k}}\times 100\%,$$

with *L* the total number of peak regions in the experimental spectrum, $N_{k}$ the number of peaks in the *k*‐peak region of the experimental spectrum, and $M_{k}$ the number of peaks in the *k*‐peak region of the DB spectrum.

Finally, for improving the database search, two parameters can be chosen by the operator. The first one is the HQI threshold value under which the results are filtered out. The second one, only available for “peak searching”, is the looseness factor (LF) that allows considering the variability in the chemical shifts of the different peaks obtained in NMR. LF corresponds to the highest difference in ppm which can be accepted to provide a match between the experimental spectra and the database (both in 1D and 2D).

### 2.6 Analytical time

The overall data acquisition and analysis times should be kept as short as possible, to remain compatible with the typical duration of a police custody (48 to 72 h in France, for instance). In this study, optimized NMR experiments last a total time of 113 min in total for each sample (see Table 1) and IR experiments take less than 1 min per acquisition. Finally, the data processing steps following acquisition are not very time‐consuming (a few minutes per experiment at most). Thus, for identification, the largest share of the processing time originates in the acquisition of analytical data. In contrast, for the elucidation of unknown NPS, the analysis of the spectral data by a trained operator is likely the rate‐limiting step, which is also dependent on the complexity of the problem to solve.

### 2.7 SWDRUGS recommendations

The choice of creating a database containing IR and NMR analytical data is perfectly justified by the recommendations of the SWDRUGS group.^14^ All analytical techniques can be separated into three different categories. Category A techniques provide the highest level of selectivity through structural information, category B techniques deliver an intermediate level of selectivity through physical or chemical characteristics but lack structural information, and category C techniques give rise to a low level of selectivity but provide general/chemical class informations. Unambiguous structure identification requires combining one category A technique to another from any category. According to SWDRUG, NMR (category A) and IR (category A) convey enough information to validate the proposed structure.

## 3 RESULTS AND DISCUSSIONS

### 3.1 NMR methods

**FIGURE 2:**
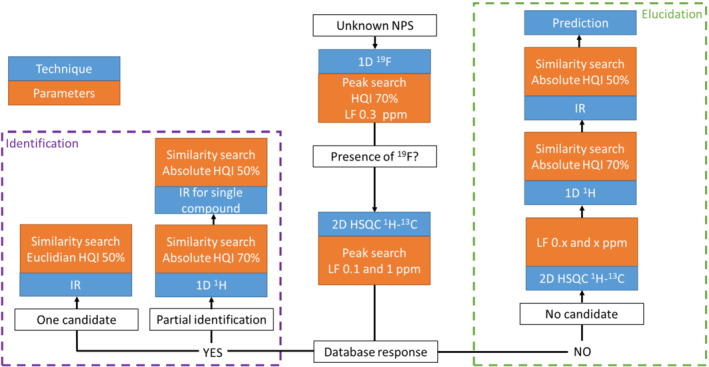
Workflow for structural identification and elucidation. For the heteronuclear single quantum coherence (HSQC) comparison in the elucidation section, the looseness factor (LF) is incremented by *x* (a nonzero natural number) [Colour figure can be viewed at wileyonlinelibrary.com]

Among the broad variety of NMR experiments employed for structural analysis, a reduced set of three experiments was chosen for our identification and elucidation workflow: 1D ^1^H, 1D ^19^F, and 2D ^1^H‐^13^C HSQC experiments. A systematic order was set up for the acquisition of these NMR experiments. First, 1D ^19^F spectroscopy was used because ^19^F nuclei were present in a minority of NPS.^18^, ^19^ This experiment allows separating unknown molecules into two categories, those with ^19^F and those without (Figure 2). Second, ^1^H‐^13^C HSQC was used as it is one of the most sensitive and informative heteronuclear 2D pulse sequences. 2D HSQC spectra were acquired with spectral edition which allows to label the ^13^C‐^1^H multiplicity for each correlation. CH and CH_3_ are represented in red (positive) and CH_2_ in blue (negative). This second experiment allows creating a double entry table with ^13^C chemical shifts in rows and ^1^H chemical shifts in columns encoded with C‐H_*n*_ parity. Therefore, provided that the LF parameter is correctly adjusted, the spectrum of the unknown NPS should either yield a unique answer or deliver no match (i.e., HQI 100 or 0). In some cases, however, the HSQC spectra recorded on a benchtop spectrometer could be limited by sensitivity due to the low natural abundance of ^13^C nuclei. This is why 1D ^1^H NMR spectra were also incorporated in the workflow, offering a more sensitive, yet less resolved technique to compare the spectral patterns of the unknown molecule with those included in the database.

### 3.2 Multi‐technique workflow

The choice of the analytical workflow is essential regarding the complexity of the sample embedding NPS. The strategy to build for identifying and elucidating NPS in seized samples has to face difficulties met to detect and quantify NPS even in mixture.

For a more comprehensive and integrated drug investigation, different multi‐methods workflows have been reported and allow for elucidating NPS^11^. They all combine multi‐analytical techniques like Raman, DSC, IR, NMR, MS, and coupled chromatographic techniques with chemoinformatic tools and commercial, in‐house databases for assisting spectral assignment, identification, and elucidation.

An applicative case where seized blotter paper^20^ was analyzed by GC–MS, UHPLC‐HRMS as well as 1D and 2D NMR in combination with multi‐methods chemometric tools like ACD/Labs, Mass Hunter, and XCalibur. This workflow led to the identification of a LSD homologous product. In the case of seized NPS in mixture with herbs, the same workflow was utilized after NPS extraction and successfully revealed a new synthetic cannabinoid.^18^

Since high‐field NMR and HRMS are expensive instruments and not owned by a majority of forensic laboratories, there is a need for cheaper and more accessible alternative workflows. We propose 1D and 2D optimized experiments implemented on liquid‐state benchtop instruments in combination with IR and dedicated associated databases to identify and also elucidate NPS. Note that our approach aims at being quite general and may not be adapted to NPS hidden or sprayed in materials that would require a more specific strategy.

For our approach, an experimental database was created with 57 entries, mainly coming from cathinones, cannabinoids, amphetamines, arylcyclohexylamines, and fentanyloids (see Table S1). Before elaborating the multi‐technique workflow, three model molecules were used to determine the best search method, the optimum algorithm for HQI calculation, the most adapted HQI threshold, as well as the optimum LF value. Table 2 summarizes the optimized parameters, details of the optimization process being provided in the SI (see S4 and S5).

**TABLE 2:** Selected parameters for spectral comparison with ACD/Labs software

Experiment	1D ^1^H	1D ^19^F	^1^H‐^13^C HSQC	IR
Search method	Similarity	Peak	Peak	Similarity
HQI algorithm	Absolute	Peak	Peak	Euclidian
HQI limit (%)	70	70	a	50
LF (ppm)	b	0.3	0.1/1	b

*Note*: a: No HQI threshold for HSQC comparison because the two possibilities are only 0 or 100. b: No LF for similarity search, LF is defined at 0.1 ppm in the ^1^H dimension and 1 ppm in the ^13^C dimension for HSQC comparison.

Abbreviations: HQI, Hit Quality Index; HSQC, heteronuclear single quantum coherence; IR, infrared; LF, looseness factor.

As described above, the workflow starting point is the 1D ^19^F analysis to determine if the molecule possesses one or several ^19^F (Figure 2). Then, comparison between the measured HSQC spectrum and the database is carried out. The result of this comparison dictates the choice between the identification and elucidation parts of the workflow. When the HSQC pulse sequence gives a single match for the unknown structure, the leftmost part of the workflow in Figure 2 is used, corresponding to identification. Nevertheless, according to the SWGDRUG guidelines, one needs to ascertain the structure of the NPS with another technique. This is done by recording the IR spectrum. There is also a small chance that HSQC provides several matches or that not all signals are assigned. In this case, both 1D ^1^H NMR and IR can be used to determine the most probable structure. The rightmost side of the workflow displayed in Figure 2 is dedicated to cases where the HSQC comparison returns no match. In such scenario, one would try to pinpoint the closest structure present inside the database, in order to provide structural insights into the molecular structure of the unknown NPS. To this end, the HSQC comparison is performed again with less restrictive LF values which are incremented until one match is obtained. This can help in determining the family of the unknown structure or part of its skeleton. Finally, IR and 1D ^1^H NMR can be used to refine the proposed structure which can be validated by comparison between the experimental HSQC spectrum and a 2D HSQC spectrum predicted by the software. Of course, full elucidation with a database can remain difficult, especially when considering the limited performance of benchtop NMR. There will be cases where conventional structure elucidation at high field, relying on a complete set of 2D experiments (including correlation spectroscopy and heteronuclear multiple bond correlation), will be required to determine the exact structure. However, the present approach is directly accessible and advantageously requires a limited level of expertise compared with a full traditional structure elucidation. Therefore, it may prove useful as an initial structure elucidation tool, able of sorting out a large number of cases in a time compatible with the requirements of the police services.

### 3.3 Database validation on real cases

A blind validation of the workflow was carried out on a set of six unknown seizures (numbered from 1 to 6) to evaluate if the database would be able to cope with concrete cases and could be routinely used in practice. All relevant data associated to these cases are given in supplementary information.

Sample no. 1 gives one signal on the 1D ^19^F spectrum. AMB‐FUBINACA (HQI = 100), AB‐FUBINACA (100), and MDMB‐FUBINACA (100) were returned as matches by comparison with the fluorine spectra present in the database. It indicates that the structure is part of the cannabinoid family and more specifically of the FUBINACA subfamily. Then, HSQC comparison provided only one hit, namely, AB‐FUBINACA (100). The structure was then easily validated by its IR spectrum. Indeed, for IR, the four top matches all belong to the cannabinoid family: AB‐FUBINACA (89.43), AB‐CHMINACA (66.56), ADB‐CHMINACA (58.88), and APINACA (53.52), which unambiguously validates AB‐FUBINACA as the actual sample no. 1 (see S6).

Sample no. 2 followed a similar workflow even if no signal appeared in the 1D ^19^F spectrum, indicating the absence of fluorine. Only one match was found with HSQC, namely, acetyl fentanyl (AF, HQI = 100). The structure was next confirmed by IR since AF (86.54) is the top match, its HQI largely exceeding the one of the second hit, PHENF with HQI of 58.85 (see S7).

Likewise, sample no. 3, that returned no match based on the 1D ^19^F spectrum, provided a single hit after the HSQC comparison, 2C‐E (100). However, when the ^1^H spectrum in the 2D projection was manually checked, it appeared that two signals in the aromatic region (6.9 and 7.3 ppm) could not be assigned to this structure. A comparison with 1D ^1^H experiments confirmed that those two signals belong to another NPS, 2C‐I (HQI 93.70), that was also present in the same sample and whose structure was similar to 2C‐E (HQI 95.85). In short, 2C‐I could be identified by the direct HSQC comparison whereas the presence of 2C‐E was found by the ^1^H NMR comparison and confirmed by visual inspection of the operator (see S8).

These first three examples highlight the efficiency of the identification component of the workflow since the database was able to systematically provide the structure of NPS obtained from seizures. In addition, the identification of a (simple) mixture could be achieved thanks to our workflow.

**FIGURE 3:**
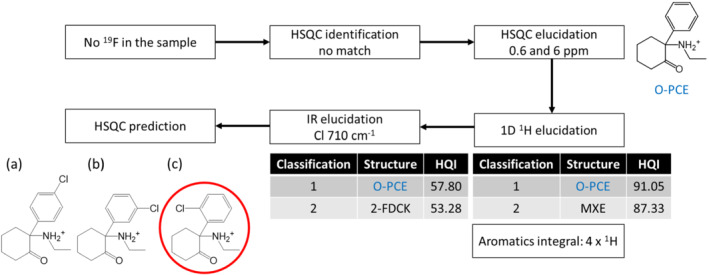
Workflow for the determination of unknown sample no. 4, finally yielding NEK as a structure (a) para substitution, (b) meta substitution, and (c) ortho substitution [Colour figure can be viewed at wileyonlinelibrary.com]

The three next samples correspond to cases where the elucidation part of the workflow was applied, because the identification failed to provide a clear hit. As illustrated in Figure 3, sample no. 4 provided no signal in 1D ^19^F spectrum, and we moved directly to the HSQC comparison. No match could be obtained using a LF of 0.1 ppm for ^1^H and 1 ppm for ^13^C. Next, we tried to find structures that would be close to the unknown one (elucidation by homology) by comparing the experimental HSQC spectrum with the database while concomitantly incrementing LF by steps of 0.1 ppm for ^1^H and 1 ppm for ^13^C. With such procedure, we obtained O‐PCE as a first match, with a LF of 0.6 ppm for ^1^H and 6 ppm for ^13^C. Then, a 1D ^1^H comparison was performed and the first two structures were O‐PCE (91.04) and MXE (87.33). All ^1^H signals had been integrated, and we found that the aromatic region integrated to four protons in agreement with a di‐substituted phenyl ring, with no major modification of the spectral pattern between the unknown structure and O‐PCE. Therefore, we attempted to better clarify the nature of substituting group on the aromatic region with the IR experiment. Again, O‐PCE came as a first hit (57.80) and a band at 710 cm^−1^ was found as a possible indicator of the presence of chlorine atom(s) in the structure (see S9). Due to the high number of IR bands and their overlap, the first hit for IR was lower than the first NMR hit for O‐PCE.

**FIGURE 4:**
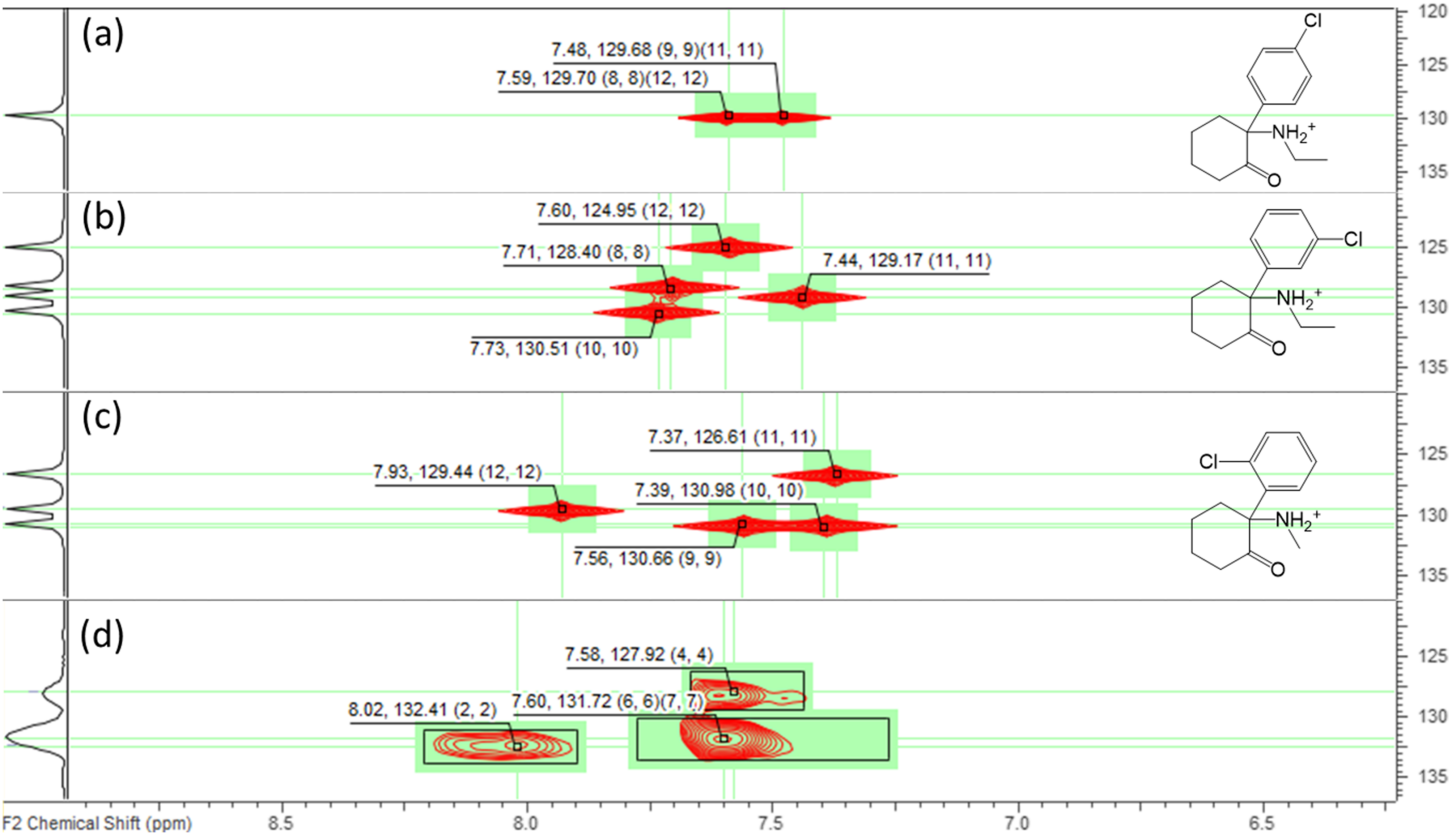
Comparison between predicted (a) para, (b) meta, (c) ortho, and (d) experimental heteronuclear single quantum coherence (HSQC) spectra for unknown sample no. 4 [Colour figure can be viewed at wileyonlinelibrary.com]

Three different positional isomers were possible in that case, with the Cl center in *ortho* (Figure 3c), *meta* (Figure 3b), or *para* (Figure 3a), and prediction was therefore needed to identify the structure. In the HSQC spectrum three correlations could be peak picked 8.02/132.4, 7.58/127.9, and 7.60/131.7 ppm, the latter apparently corresponding to two different protons (the signal intensity was much higher for this one than for the two others correlations). With those information at hand, *para* substitution could already be discarded as only two correlations would be expected for this isomer. Next, the Predictor ACD/Labs module was used to predict the chemical shift of the three isomers (Figure 4). The closest match with the actual measured spectrum of sample no. 4 was obtained for the *ortho* isomer; this molecule is called NEK (Figure 3c). For further validation, the structure was later confirmed by high‐field experiments performed at 700 MHz.

The workflow for sample no. 5 started directly with HSQC since no signal was observed in the 1D ^19^F spectrum. No match could be found with the identification parameters given in Table 2. As above, we incremented LF until 0.5 ppm for ^1^H and 5 ppm for ^13^C, values leading to PV9 as the closest structure. The HSQC spectra of the unknown compound and of PV9 are reported in S10. In the aromatic region PV9 presents three correlations representative of a monosubstituted species, whereas substance no. 5 shows two correlations, a pattern consistent with a *para* bisubstitution (CH_3_ correlation at 0.73 and 13.47 ppm). Correlations for protons number 9, 12, 13, 14, and 15 are identical in the two spectra. A correlation at 1.98 ppm (^1^H) and 29 ppm (^13^C) is present for both structures, this CH_2_ being the beginning of the side chain. Then, two CH_2_ and one CH_3_ remained unassigned with this HSQC comparison. Then, a 1D ^1^H NMR comparison was achieved to obtain more information on the nature of the lateral chain. Two different chain lengths were present in the three first hit: propyl (PVP, MDPV) and hexyl (PV9). However, visually, those spectra strongly differed from the one of our unknown structure. In addition, unknown compound no. 5 could not contain only methyl or ethyl groups since the NMR spectra would respectively show a doublet or a triplet coupled to a quadruplet in these two cases (see S10 Figure S1). IR comparison was also made, and the same structures as before were identified as the closest to our structure. Finally, the HSQC prediction was done considering a butyl side chain (3 CH_2_ and 1 CH_3_) and it returned a map closely resembling one of the unknown samples (Figure S10a). Thus, the structure was identified as MPHP with a butyl side chain, and this was confirmed by 700 MHz experiments (see S10).

The two previous cases demonstrate that elucidation with the help of a database can be carried out relying on benchtop NMR combined with IR spectroscopy. The two new structures were eventually added to the database. Indeed, the database can be improved while previously unknown NPS are discovered, allowing a continuous improvement of its identification and elucidation abilities.

For the last case, sample no. 6, the usual workflow was followed. The 1D ^19^F comparison was not performed since no fluorine signal could be observed on the spectrum. Then, the comparison with HSQC provided no match with the LF factors given in Table 2. Therefore, LF was incremented until a match was obtained with a LF of 0.5 for ^1^H and 5 ppm for ^13^C. The proposed structure with this comparison is NEK, the sample that was added to the database just after processing sample no. 4. To confirm that structure, 1D ^1^H comparison was used and provided NEK (92.50), O‐PCE (89.72), and HEXEN (86.56) as the three best matches. However, when spectral patterns of those two spectra were compared, it could be seen that additional signals were present in sample no. 6 (see S11). The presence of a mixture explains why LF needed to be increased in order to obtain a match with HSQC. The two components of this mixture were determined and validated at 700 MHz (see S11): It turns out that the sample is a mixture of NEK and MDMC. This shows that although benchtop NMR failed to identify all the analytes in the mixture, the major component (NEK) could be identified relying on our workflow.

To summarize, six cases were examined using the previously constructed workflow. In the first three cases, AF, AB‐FUBINACA, and a mixture of 2C‐I and 2C‐E, were straightforwardly identified with the help of their reference spectra in the database. The two next samples were elucidated with the help of the database to determine the structures of NEK and MPHP (see S10 and S11). Finally, the last sample could not be fully characterized by our workflow which nevertheless identified NEK as the major component of the mixture. The strategy based on structure similarity was also reported with LC/ESI/HRMS in the field of a non‐targeted screening.^21^ This approach also made it possible to access semiquantitative data of main interest for tracking drugs.

### 3.4 Quantification

To complete our study, the purity of the fully characterized NPS was measured by quantitative benchtop NMR. Purity determination is an additional data to trace the manufacturers and to further dismantle drug networks. It is generally performed by GC‐FID or LC‐DAD analysis, but these techniques require finding the corresponding reference material, which is not systematically accessible and can also be prohibitively expensive. Therefore, NMR appears as a valuable alternative because, on the one hand, quantification can be achieved without physical separation of the sample components, and on the other hand, multiple compounds can be precisely quantified with a single internal or external reference, which does not necessarily have to be close to the analyte (in contrast to chromatography). An internal reference was not used here since avoiding overlap with NMR signals was beyond reach for the benchtop apparatus. Therefore, the choice was made to determine the purity using TSP as an external reference. Purity determination was carried out relying on the following equation:

(4) $$P_{\mathrm{NPS}}=P_{\mathrm{TSP}}\times \left(\frac{A_{x}}{A_{\mathrm{TSP}}}\right)\times \left(\frac{N_{\mathrm{TSP}}}{N_{x}}\right)\times \left(\frac{M_{x}}{M_{\mathrm{TSP}}}\right)\times \left(\frac{m_{\mathrm{TSP}}}{m_{x}}\right),$$

where $P_{\mathrm{TSP}}\;$ is the purity of the external standard TSP, $A_{x}$ the integral of the analyte signal, $A_{\mathrm{TSP}}\;$ the integral of the reference signal, $N_{\mathrm{TSP}}$ the number of reference protons, $N_{x}$ the number of analyte protons, $M_{x}$ the molecular weight of the analyte (mg/mol), $M_{\mathrm{TSP}}$ the molecular weight of the reference (mg/mol), $m_{\mathrm{TSP}}$ the mass of reference compound (g), and $m_{x}$ the mass of sample (g).

Then, the precision of the purity was determined on five successive spectra for each sample, and it was calculated with the following formula.

(5) $$P\left(\%\right)=\mathrm{CV}\left(A_{x}\right)+\mathrm{CV}\left(A_{\mathrm{TSP}}\right)+\left(\frac{∆m_{x}}{m_{x}}\right)\times 100+\left(\frac{∆m_{\mathrm{TSP}}}{m_{\mathrm{TSP}}}\right)\times 100,$$

where $\mathrm{CV}\left(A_{x}\right)$ is the standard deviation of the reference integral determined with five repetitions, $\mathrm{CV}\left(A_{\mathrm{TSP}}\right)\;$ is the standard deviation of the analyte integral determined with five repetitions, $∆m_{x}$ and $∆m_{\mathrm{TSP}}$ equal to 0.01 mg, and $m_{x}$ and $m_{\mathrm{TSP}}$ have been define above.

NMR quantification with 1D ^1^H benchtop NMR can be difficult, because it is necessary to find at least one signal per compound which is not overlapped with others and where the baseline reaches zero on both sides to reach an accurate (true and precise) quantification. Thus, purity of the substance, nature of the substance, and sample/matrix interferences could impact the accuracy of the quantification. For each of the previous samples, the signal which was the least overlapped with others was used (see S12). Calculated purity values are displayed in Table 3. The two pure identified structures have a purity higher than 90% with a precision of approximately 2%. For the identified mixture, the purities of 2C‐I and 2C‐E were determined as 66% and 21%, respectively, with again a precision close to near 2%. MPHP was determined to be pure at more than 90%. Finally, the purity of NEK was determined to be much higher that 100%, and this awkward result is related to the absence of signal free of overlapping in the 1D spectrum. Signals integrated for the determination of purity belonged to the aromatic region which were presumably overlapped with signals of a minor component that could not be detected with a magnetic field under 100 MHz (some signals could be detected but not identified at 700 MHz). With a benchtop NMR spectrometer, quantitative 1D NMR could be limited when seized samples are complex, for example, embedding different types of compounds and impurities. Thus, in these cases 2D quantitative NMR could be relevant, as well as the combination with other chromatographic‐based separations in case of highly complex mixtures.

**TABLE 3:** Purity obtained for NPS samples 1 to 6 and associated precision

Sample no.	Molecule	Purity (%)	Precision (%)
1	AB‐FUBINACA	90.2	1.8
2	AF	99.0	2.2
3	2C‐E	66.4	2.4
3	2C‐I	21.3	3.1
4	NEK	113.5	2.6
5	MPHP	94.8	4.5

## 4 CONCLUSION

An integrative workflow was created to help identifying NPS. It includes benchtop 1D and 2D NMR, IR, and prediction together with an experimental database to characterize NPS according to the directives of the SWGDRUGS workgroup. The database includes data for 57 compounds but will be enriched with more samples in the near future. The efficiency of the workflow was evaluated on six seizures. Most structures were unambiguously identified or elucidated, even in the case of mixtures. This evaluation also highlighted the limitation of the workflow when significant overlap between peaks occurs. In some cases, an expert or habituated eye will most likely be needed, but we are confident that the workflow presented in this study would facilitate the identification process.

Additionally, purity was also determined for each sample thanks to 1D ^1^H NMR. Precise purities were obtained except for one case where the NMR signal was heavily overlapped with the one of another molecule. To the best of our knowledge, this work stands as the first example of using benchtop NMR in an integrated workflow for identifying, elucidating and quantifying NPS. This workflow can serve as an alternative to the use of GC–MS: It could allow forensic laboratories to efficiently characterize and quantify a wider panel of drugs and especially NPS.

The present workflow and database work well for simple elucidation cases, for more difficult ones, the creation of a bigger database including more reference structures is needed. In addition, the proposed workflow could be combined with the current workflows already used by the police, by including GC–MS in the database to maximize its potential. Another appealing perspective would be to combine those developments with machine learning, to create an even more automated and reliable elucidation procedure.

## CONFLICT OF INTEREST

None.

## Supporting information


**Data S1.** Supporting Information

## Data Availability

Data available on request from the authors.
