# Supplementary material for: Characterization of new psychoactive substances by integrating benchtop NMR to multi‐technique databases
Source: Drug Test Anal. 2022 Jun 26;14(9):1629–38. doi: 10.1002/dta.3332 (PMC9545896; doi:10.1002/dta.3332)
Supplement: Supplementary file 1 — Data S1. Supporting Information [file DTA-14-1629-s001.docx]

Supplementary information: Characterization of new psychoactive substances by integrating benchtop NMR to multi-technique databases

Thomas Castaing-Cordier^a^, Alejandra Benavides Restrepo^a^, Damien Dubois^b^, Virginie Ladroue^b^, Fabrice Besacier^c^, Audrey Bulete^b^, Céline Charvoz^b^, Anais Goupille^a^, Denis Jacquemin^a^, Patrick Giraudeau^a^ and Jonathan Farjon^a,*^

^a^Nantes Université, CNRS, CEISAM UMR 6230, F-44000 Nantes, France

^b^Service National de police scientifique, Laboratoire de police scientifique de Lyon, 69134 Ecully, France

^c^Service National de police scientifique, Sous-direction de la stratégie de l’innovation et du pilotage, 69134 Ecully, France

*Corresponding author: jonathan.farjon@univ-nantes.fr

[S1: Database entries 4](#_Toc103005731)

[S2: ^1^H-^13^C HSQC spectral comparison 16](#_Toc103005732)

[S2: SNR comparison of different ^1^H-^13^C HSQC 17](#_Toc103005733)

[S3: PH HSQC ME pulse program 18](#_Toc103005734)

[S4: LF, HQI optimization 25](#_Toc103005735)

[S5: 1D ^1^H 3-MMC peak search 27](#_Toc103005736)

[S5: 1D ^1^H 4-MMC peak search 28](#_Toc103005737)

[S5: 1D ^1^H 3-MMC absolute algorithm 29](#_Toc103005738)

[S5: 1D ^1^H 3-MMC euclidian algorithm 30](#_Toc103005739)

[S5: 1D ^1^H 4-MMC absolute algorithm 31](#_Toc103005740)

[S5: 1D ^1^H 4-MMC euclidian algorithm 32](#_Toc103005741)

[S5: 1D ^19^F AB-FUBINACA similarity search 33](#_Toc103005742)

[S5: 1D ^19^F AB-FUBINACA peak search 34](#_Toc103005743)

[S5: HSQC 3-MMC identification 35](#_Toc103005744)

[S5: HSQC 4-MMC identification 36](#_Toc103005745)

[S5: HSQC 4-MMC spectral homology 37](#_Toc103005746)

[S5: IR 3-MMC peak search 38](#_Toc103005747)

[S5: IR 3-MMC absolute algorithm 39](#_Toc103005748)

[S5: IR 3-MMC euclidian algorithm 40](#_Toc103005749)

[S5: IR 4-MMC peak search 41](#_Toc103005750)

[S5: IR 4-MMC absolute algorithm 42](#_Toc103005751)

[S5: IR 4-MMC euclidian algorithm 43](#_Toc103005752)

[S6: 1D ^19^F substance n°1 44](#_Toc103005753)

[S6: 2D HSQC substance n°1 45](#_Toc103005754)

[S6: IR substance n°1 46](#_Toc103005755)

[S6 : Identification substance 1 at 700MHz 47](#_Toc103005756)

[S7: 2D HSQC substance n°2 50](#_Toc103005757)

[S7: IR substance n°2 51](#_Toc103005758)

[S7: Identification substance 2 at 700MHz 52](#_Toc103005759)

[S8: 2D HSQC substance n°3 55](#_Toc103005760)

[S8: 1D ^1^H substance n°3 56](#_Toc103005761)

[S8: Identification substance 3 at 700MHz 57](#_Toc103005762)

[S9: 2D HSQC identification substance n°4 58](#_Toc103005763)

[S9: 2D HSQC elucidation substance n°4 59](#_Toc103005764)

[S9: 1D ^1^H substance n°4 60](#_Toc103005765)

[S9: IR substance n°4 61](#_Toc103005766)

[S9: Elucidation substance n°4 at 700MHz 62](#_Toc103005767)

[S10: 2D HSQC identification substance n°5 65](#_Toc103005768)

[S10: 2D HSQC elucidation substance n°5 66](#_Toc103005769)

[S10: 1D ^1^H substance n°5 67](#_Toc103005770)

[S10: IR substance n°5 68](#_Toc103005771)

[S10: HSQC prediction for MPHP 69](#_Toc103005772)

[S10A: 700 MHz and predicted HSQC comparison 70](#_Toc103005773)

[S10: Elucidation substance n°5 at 700MHz 71](#_Toc103005774)

[S11: 2D HSQC identification substance n°6 74](#_Toc103005775)

[S11: 2D HSQC elucidation substance n°6 75](#_Toc103005776)

[S11: 1D ^1^H substance n°6 76](#_Toc103005777)

[S11: Elucidation substance 6 at 700MHz 77](#_Toc103005778)

[S12: Integrations area for the determination of purity 80](#_Toc103005779)

S1: Database entries

| Name | Structure | NMR concentration (mM) | NMR | IR |
| --- | --- | --- | --- | --- |
| 2-AI | 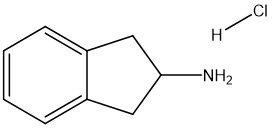 | 295 | Yes | Yes |
| 2-BMMP |  | 306 | Yes | Yes |
| 2C-E | 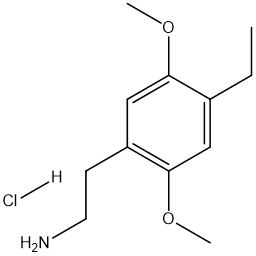 | 298 | Yes | Yes |
| 2C-I | 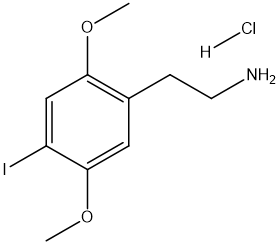 | 258 | Yes | Yes |
| 2-FDCK | 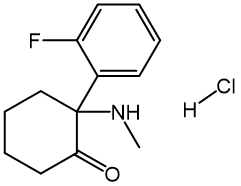 | 288 | Yes | Yes |
| 2C-B | 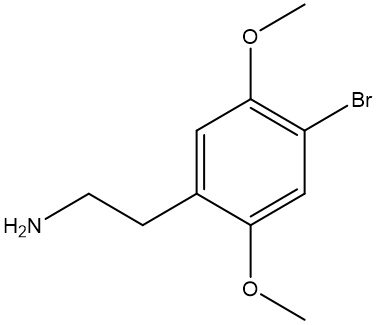 | x | No | Yes |
| 3-CMC | 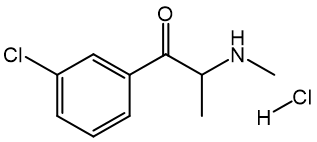 | 291 | Yes | Yes |
| 3-FPM | 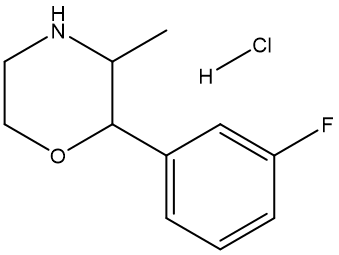 | 307 | Yes | Yes |
| 3-MMC | 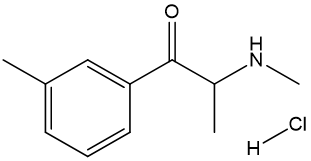 | 299 | Yes | Yes |
| 4-CEC | 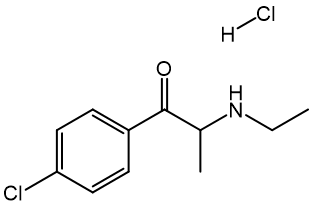 | 150 | Yes | Yes |
| 4-EPD |  | 315 | Yes | Yes |
| 4F-MPH | 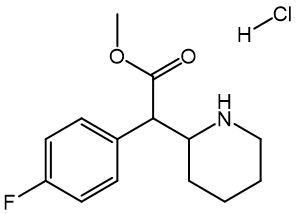 | 317 | Yes | Yes |
| 4F-A | 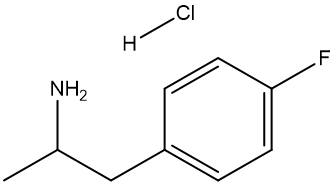 | 273 | Yes | Yes |
| 4-MEC | 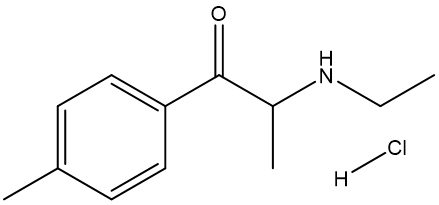 | 258 | Yes | Yes |
| 4-MeO-PCP | 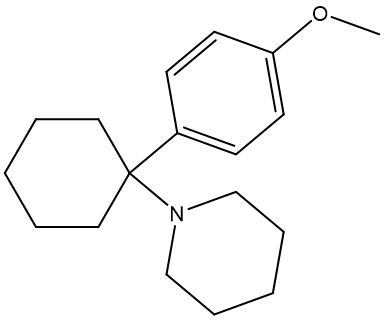 | 305 | Yes | Yes |
| 4-MMC | 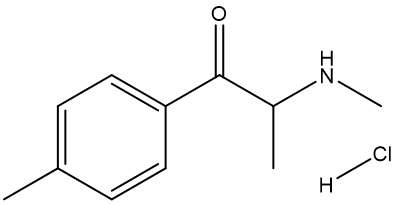 | 243 | Yes | Yes |
| 5-APB | 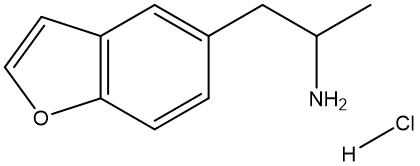 | 298 | Yes | Yes |
| 5F-ADB | 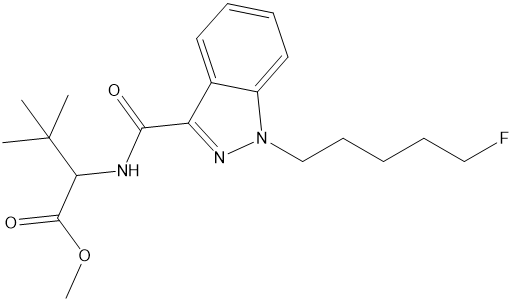 | 273 | Yes | Yes |
| 5-MeO-DALT | 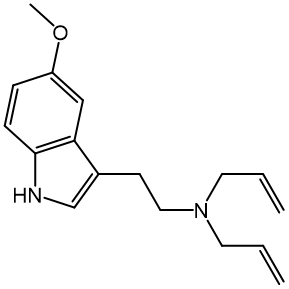 | X | No | Yes |
| 5F-MDMB-PICA | 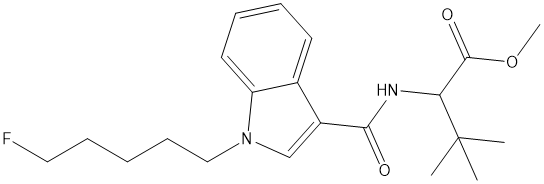 | 330 | Yes | Yes |
| 5F-PB-22 | 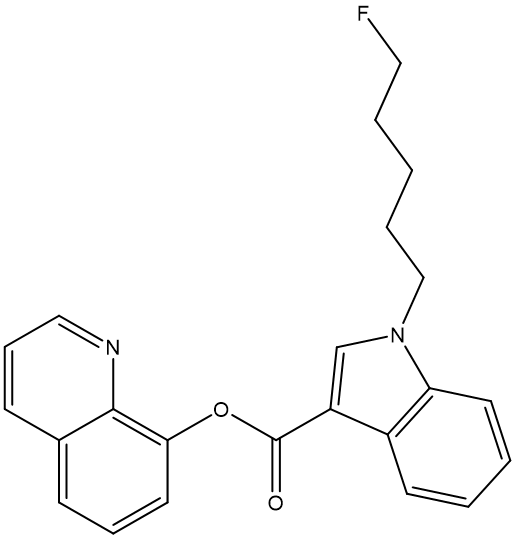 | 147 | Yes | Yes |
| **AMT** | **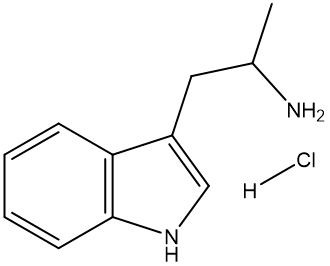** | **277** | **Yes** | **Yes** |
| α-PVP | 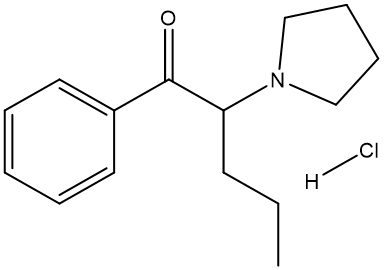 | 265 | Yes | Yes |
| AB-CHMINACA | 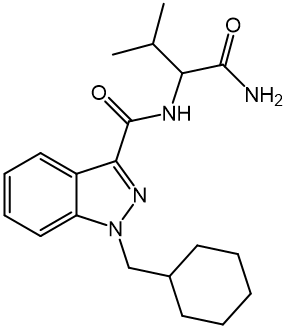 | 312 | Yes | Yes |
| AB-FUBINACA | 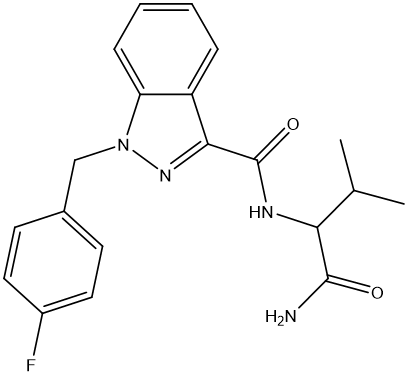 | 291 | Yes | Yes |
| ADB-CHMINACA | 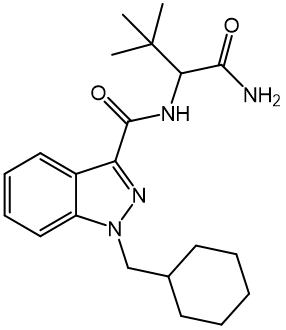 | 279 | Yes | Yes |
| AF | 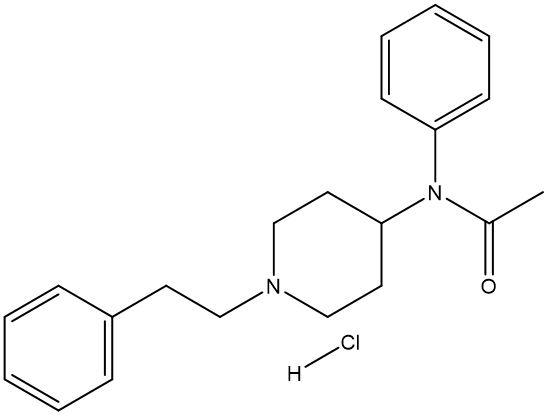 | 276 | Yes | Yes |
| AMB-CHMICA | 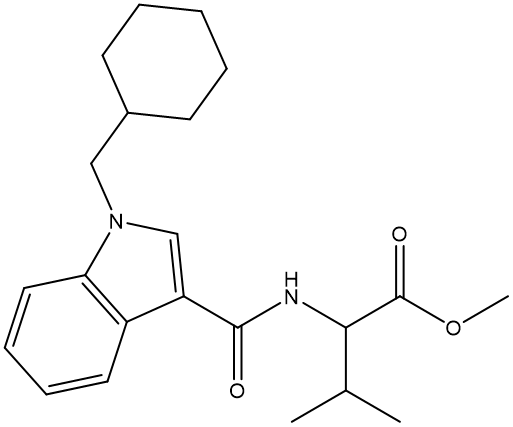 | 275 | Yes | Yes |
| AMB-FUBINACA | 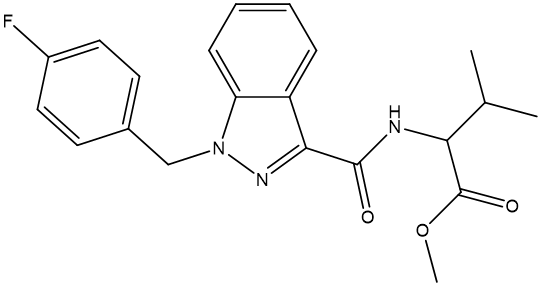 | 248 | Yes | Yes |
| AMF | 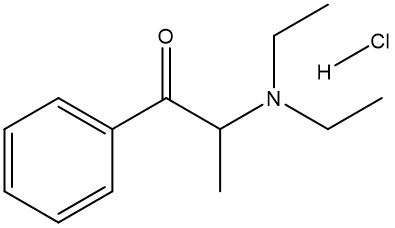 | 236 | Yes | Yes |
| AMP | 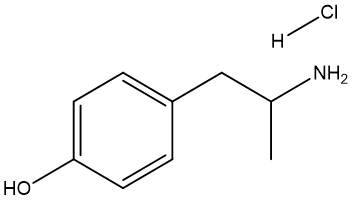 | 300 | Yes | Yes |
| APINACA |  | 301 | Yes | Yes |
| bk-MBDB | 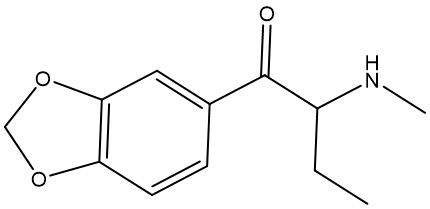 | X | No | Yes |
| bk-MPA |  | 286 | Yes | Yes |
| BPR | 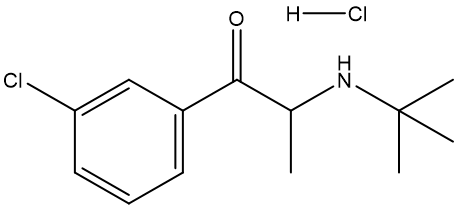 | 319 | Yes | Yes |
| DMPEA-NBTOMe |  | 268 | Yes | Yes |
| DPD | 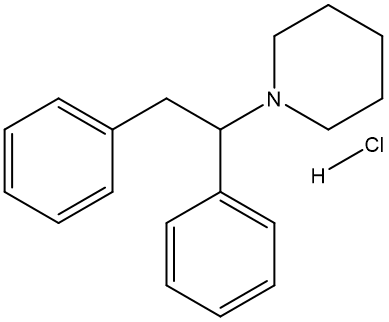 | 297 | Yes | Yes |
| ETH-CAT |  | 310 | Yes | Yes |
| FIBF | 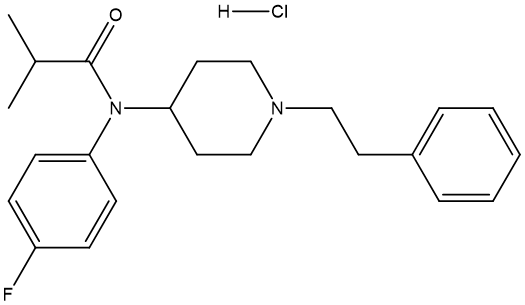 | 206 | Yes | Yes |
| HEXEN | 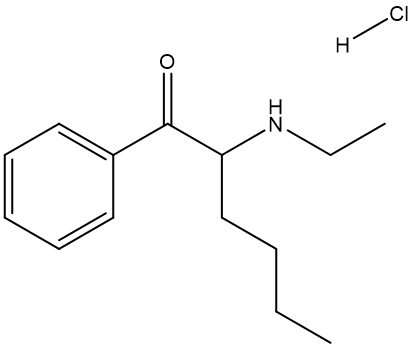 | 310 | Yes | Yes |
| JWH210 | 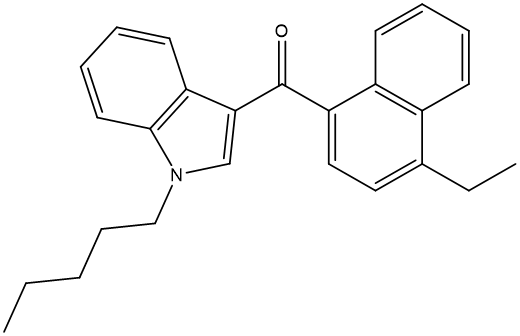 | 265 | Yes | Yes |
| MDAI | 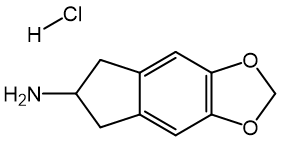 | 275 | Yes | Yes |
| MDEC | 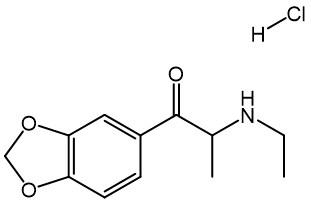 | 295 | Yes | Yes |
| MDMB-FUBINACA | 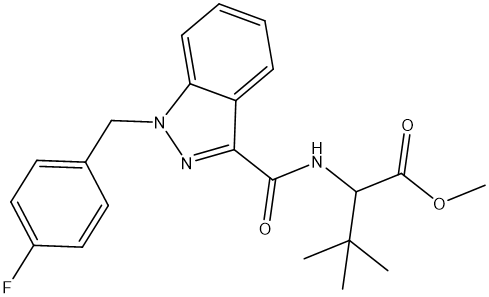 | 290 | Yes | Yes |
| MDMB-CHMCZCA | 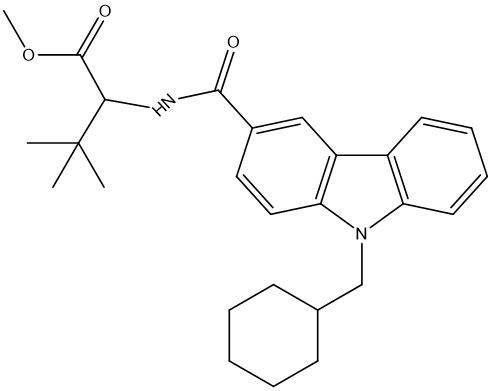 | 300 | Yes | Yes |
| MDMB-CHMICA | 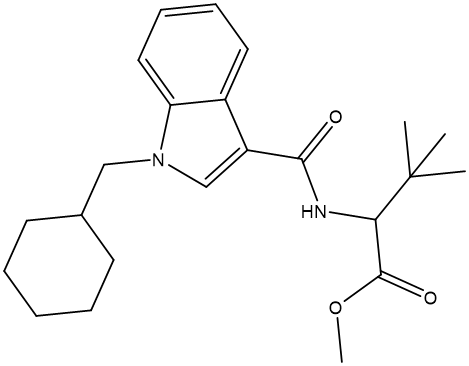 | 260 | Yes | Yes |
| MDMC | 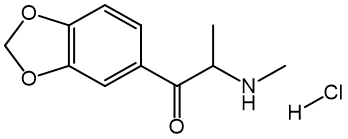 | 310 | Yes | Yes |
| MDPV | 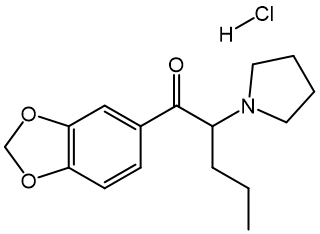 | 110 | Yes | Yes |
| MPHP | 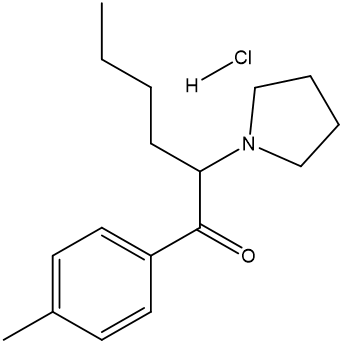 | 200 | Yes | Yes |
| MXE | 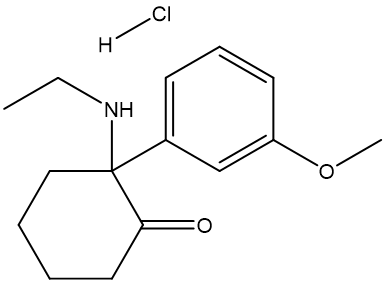 | 144 | Yes | Yes |
| NEK | 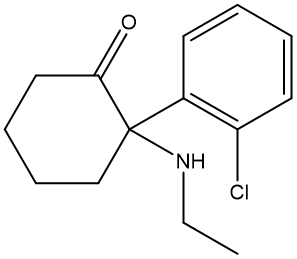 | 203 | Yes | Yes |
| O-PCE | 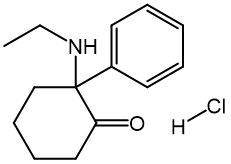 | 150 | Yes | Yes |
| PHENF | 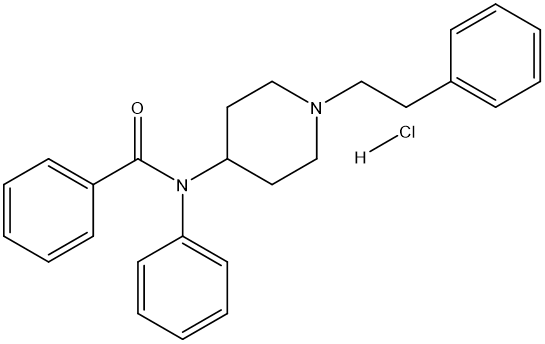 | 217 | Yes | Yes |
| PV9 | 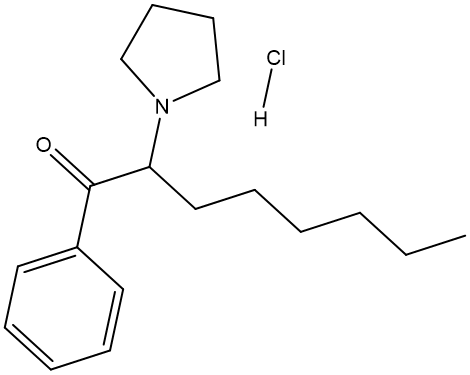 | 250 | Yes | Yes |
| TFMPP | 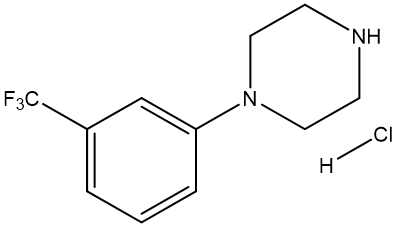 | 295 | Yes | Yes |
| U-47700 | 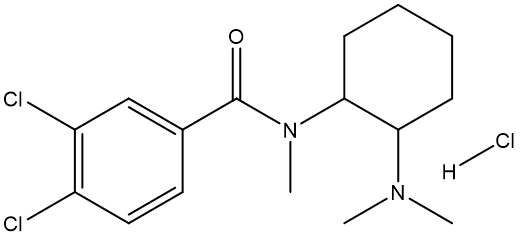 | 300 | Yes | Yes |
| UR-144 | 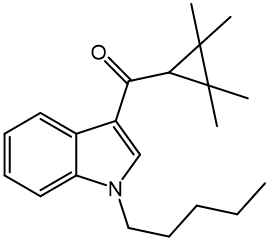 | 306 | Yes | Yes |

S2: ^1^H-^13^C HSQC spectral comparison


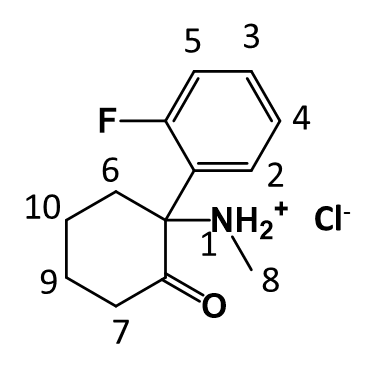


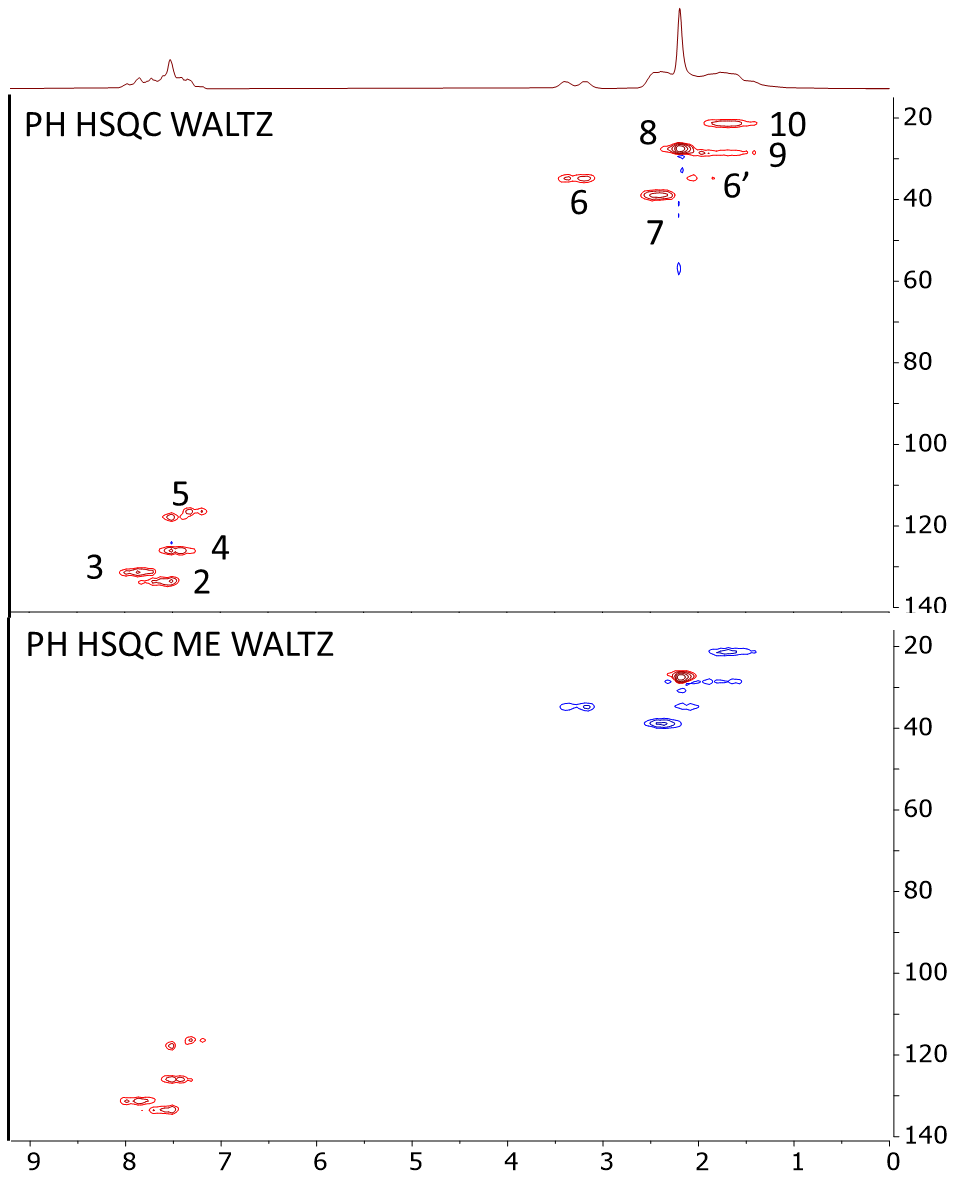


S2: SNR comparison of different ^1^H-^13^C HSQC


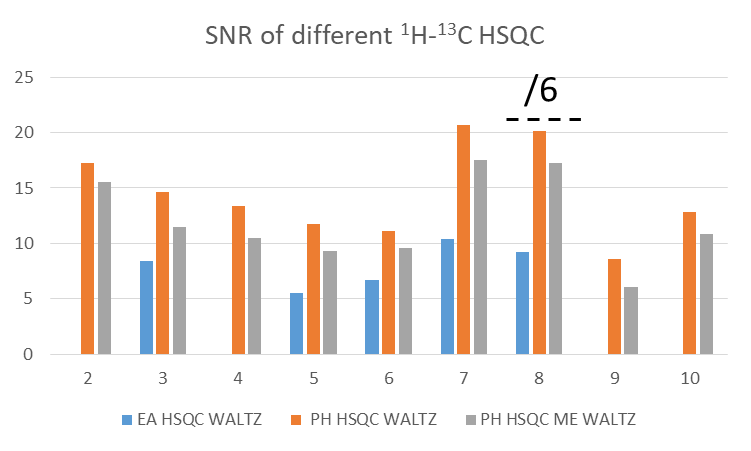


S3: PH HSQC ME pulse program

Procedure (pulse_program,dir,mode)

# Interface description (name, label, x, y, ctrl, vartype)

interface = ["nucleus", "Nuclei","0","0", "tb", "readonly_string",

"b1Freq1H", "Proton Frequency (MHz)","0","0", "tbw","freq",

"offFreq", "Offset Frequency (Hz)","0","1", "tbw","float",

"pulseLength13C", "Pulse length for all(us)", "0","2", "tbw","pulselength",

"90AmplitudeHC", "90 Pulse amplitude (dB)", "0","3", "tbw","pulseamp",

"180AmplitudeHC", "180 Pulse amplitude (dB)", "0","4", "tbw","pulseamp",

"b1Freq13C", "Carbon Frequency (MHz)", "1","0", "tbw","freq",

"90Amplitude13C", "90 Pulse amplitude (dB)", "1","1", "tbw","pulseamp",

"spoilAmp", "Homospoil amplitude", "1","2", "tbw","float,[1,1e4]",

"spoilDur", "Homospoil duration (us)", "1","3", "tbw","sdelay",

"gradStab", "Grad ringdown time (us)", "1","4", "tb", "sdelay",

"aCDec", "Decouple amplitude (dB)", "1","5", "tbw","pulseamp",

"pulseLengthC180", "Decouple length 180 (us)", "1","6", "tbw","pulselength",

"jch", "J(C,H) (Hz)", "2","2", "tbw","float",

"bandwidth2", "Bandwidth2 (kHz)", "2","3", "tbw","float,[1,1e5]",

"nrSteps","Number of steps","2","4", "tbw","integer,[1,1024]",

"repTime","Repetition time (ms)", "3","1", "tbw","reptime",

"dummyCycles", "Number of dummy cycles", "3","2", "tb", "integer,[0,16]",

"gradStabEnd", "Grad ringdown time (us)", "4","4", "tb", "sdelay"

# Relationships to determine remaining variable values

relationships = ["b1Freq = b1Freq1H",

"d9 = 1",

"d4 = 1",

"n1 = nrPnts",

"n3 = nrEchoes",

"n4 = xshim",

"n5 = yshim",

"n6 = zshim",

"n7 = xshim-(spoilAmp*xshim/(abs(xshim)+0.1))",

"n8 = yshim-(spoilAmp*yshim/(abs(yshim)+0.1))",

"n9 = zshim-(spoilAmp*zshim/(abs(zshim)+0.1))",

"n10 = xshim-(0.75*spoilAmp*xshim/(abs(xshim)+0.1))",

"n11 = yshim-(0.0*spoilAmp*yshim/(abs(yshim)+0.1))",

"n12 = zshim-(0.0*spoilAmp*zshim/(abs(zshim)+0.1))",

"n13 = xshim+(spoilAmp*xshim/(abs(xshim)+0.1))",

"n14 = yshim+(spoilAmp*yshim/(abs(yshim)+0.1))",

"n15 = zshim+(spoilAmp*zshim/(abs(zshim)+0.1))",

"a1 = 90AmplitudeHC",

"a2 = 180AmplitudeHC",

"a3 = 90Amplitude13C",

"a4 = 0",

"a5 = -85",

"a6 = aCDec",

"d1 = pulseLength13C",

"d12 = pulseLengthC180/2",

"d13 = 3*pulseLengthC180/2",

"d14 = 2*pulseLengthC180",

"d6 = pulseLengthC180",

"d7 = spoilDur",

"d8 = gradStab", # gradient settle delay

"d2 = 1e6/4/jch-2*pulseLength13C/3.14",

"d3 = 1e6/3/jch-pgo-pulseLength13C/2",

"d10 = 1e6/2/jch-pgo-2*pulseLength13C/3.14",

"d20 = 3.976/4*d7",

"w1 = 1e3/2/bandwidth2",

"d5 = acqDelay",

"O1 = offFreq",

"f1 = double(b1Freq1H)+double(offFreq/1e6d)",

"f2 = 10d*f1",

"f3 = double(b1Freq13C)",

"a111 = -80",

"totPnts = nrPnts",

"totTime = acqTime"]

# Define the tabs and their order

tabs = ["Pulse_sequence","Progress","Acquisition2d",

"Processing_Std","Display_2D","File_Settings"]

# These parameters will be changed between experiments

variables = ["w1"]

# x and y spacing between controls

dim = [190,26]

# Pulse sequence

initpp(dir) # Reset internal parameter list

cleardata(n1)

settxfreqs(f1,f3)

delay(5000) # allow time to finish lock scan

#BIRD module

pulse(1,a1,p1,d1) # 90 H RF pulse, phase 0

delay(d10) # 1/2J

pulse(1,a1,p3,f1,2,a3,p3,f3,d1) # 90 HC pulse

delay(1) # rf delay

pulse(1,a2,p1,f1,2,a4,p1,f3,d1) # 180 HC pulse

delay(1) # rf delay

pulse(1,a1,p3,f1,2,a3,p3,f3,d1) # 90 HC pulse

delay(d10) # 1/2J

pulse(1,a1,p13,d1) # 90 H RF pulse, phase 2

#350 ms delay hardcoded for now

delay(175000) #nulling delay

delay(175000) #nulling delay

pulse(1,a1,p1,d1) # 90 H RF pulse

delay(d2) # coupling delay

pulse(1,a1,p2,f1,2,a3,p5,f3,d1) # 90 HC pulse

delay(1) # rf delay

pulse(1,a2,p3,f1,2,a4,p7,f3,d1) # 180 HC pulse

delay(1) # rf delay

pulse(1,a1,p2,f1,2,a3,p5,f3,d1) # 90 HC pulse

delay(d2) # coupling delay

#Split HC pulse into separate H and C pulses - Z filter between two

pulse(1,a1,p3,d1) # 90 H RF pulse

delay(10)

shim16(1,n7) # x

shim16(2,n8) # y

shim16(0,n9) # z

delay(d7) # homospoil delay

delay(d7) # homospoil delay

delay(d7) # homospoil delay

delay(d7) # homospoil delay

delay(d7) # homospoil delay

shim16(1,n4) # x

shim16(2,n5) # y

shim16(0,n6) # z

delay(d8) # settle delay

pulse(2,a3,p8,d1) # 90 C RF pulse

wait(w1) # evolution delay

pulse(1,a1,p5,d1) # 90 H pulse

delay(1) # rf delay

pulse(1,a2,p4,d1) # 180 H pulse

delay(1) # rf delay

pulse(1,a1,p5,d1) # 90 H pulse

wait(w1) # evolution delay

#Mutiplicity-editing

delay(d10) # 1/2J

pulse(1,a1,p3,f1,2,a3,p3,f3,d1) # 90 HC pulse

delay(1) # rf delay

pulse(1,a2,p1,f1,2,a4,p1,f3,d1) # 180 HC pulse

delay(1) # rf delay

pulse(1,a1,p3,f1,2,a3,p3,f3,d1) # 90 HC pulse

delay(d10) # 1/2J

#End of ME

#Split HC pulses and use Z filter again

pulse(2,a3,p9,d1) # 90 C RF pulse

delay(10)

shim16(1,n7) # x

shim16(2,n8) # y

shim16(0,n9) # z

delay(d7) # homospoil delay

delay(d7) # homospoil delay

delay(d7) # homospoil delay

delay(d7) # homospoil delay

shim16(1,n4) # x

shim16(2,n5) # y

shim16(0,n6) # z

delay(d8) # settle delay

pulse(1,a1,p5,d1) # 90 H RF pulse

delay(d2) # coupling delay

pulse(1,a1,p3,f1,2,a3,p5,f3,d1) # 90 HC pulse

delay(1) # rf delay

pulse(1,a2,p6,f1,2,a4,p10,f3,d1) # 180 HC pulse

delay(1) # rf delay

pulse(1,a1,p3,f1,2,a3,p5,f3,d1) # 90 HC pulse

delay(d2) # coupling delay

#Acquisition and decoupling

acquireon(n1)

loop(l1,n2)

pulse(2,a6,p12,d13); delay(d9); pulse(2,a6,p11,d14); delay(d9); pulse(2,a6,p12,d6); delay(d9); pulse(2,a6,p11,d13); delay(d9); pulse(2,a6,p12,d12); delay(d9); pulse(2,a6,p11,d6); delay(d9); pulse(2,a6,p12,d14); delay(d9); pulse(2,a6,p11,d6); delay(d9); pulse(2,a6,p12,d13);

delay(d4);

pulse(2,a6,p11,d13); delay(d9); pulse(2,a6,p12,d14); delay(d9); pulse(2,a6,p11,d6); delay(d9); pulse(2,a6,p12,d13); delay(d9); pulse(2,a6,p11,d12); delay(d9); pulse(2,a6,p12,d6); delay(d9); pulse(2,a6,p11,d14); delay(d9); pulse(2,a6,p12,d6); delay(d9); pulse(2,a6,p11,d13);

delay(d4);

pulse(2,a6,p11,d13); delay(d9); pulse(2,a6,p12,d14); delay(d9); pulse(2,a6,p11,d6); delay(d9); pulse(2,a6,p12,d13); delay(d9); pulse(2,a6,p11,d12); delay(d9); pulse(2,a6,p12,d6); delay(d9); pulse(2,a6,p11,d14); delay(d9); pulse(2,a6,p12,d6); delay(d9); pulse(2,a6,p11,d13);

delay(d4);

pulse(2,a6,p12,d13); delay(d9); pulse(2,a6,p11,d14); delay(d9); pulse(2,a6,p12,d6); delay(d9); pulse(2,a6,p11,d13); delay(d9); pulse(2,a6,p12,d12); delay(d9); pulse(2,a6,p11,d6); delay(d9); pulse(2,a6,p12,d14); delay(d9); pulse(2,a6,p11,d6); delay(d9); pulse(2,a6,p12,d13);

delay(d4);

delay(10);

endloop(l1)

acquireoff("overwrite",n1)

shim16(1,n7) # x

shim16(2,n8) # y

shim16(0,n9) # z

delay(100000) # homospoil delay

shim16(1,n4) # x

shim16(2,n5) # y

shim16(0,n6) # z

delay(50000)

lst = endpp(mode) # Return parameter list

# Phase cycle list

phaseList = [0,0,0,0; # phase 1

0,0,0,0; # phase 2

1,1,1,1; # phase 3

0,0,0,0; # phase 4

1,1,1,1; # phase 5

0,0,0,0; # phase 6

0,0,0,0; # phase 7

0,2,0,2; # phase 8

0,0,2,2; # phase 9

0,0,0,0; # phase 10

0,0,0,0; # Decouple phase 1

2,2,2,2; # Decouple phase 2

2,2,2,2; # BIRD

0,2,2,0] # Acquire phase

endproc(lst,tabs,interface,relationships,variables,dim,phaseList)

S4: LF, HQI optimization

For 1D ^1^H, we evaluated both types of search comparison on 3-MMC and 4-MMC but the best results were obtained with the similarity search applied to a spectral width between -1 and 12 ppm. This is due to the fact that 60 MHz ^1^H NMR spectra are overcrowded and highly hampered by overlapping, which makes the peak searching method inefficient. In most cases, a large part of the database is returned by the software with an HQI between 0 and 100, therefore only the first four results will be presented. For the identification of 3-MMC, the following results were obtained with an absolute similarity search (HQI values are indicated in brackets for each hit): 3-MMC (96.45), 4-MMC (94.94), 3-CMC (91.79) and 4-MEC (90.13). The following results were obtained with a Euclidian search: 4-MMC (74.81), 3-CMC (69.99), 4-MEC (67.85) and 3-MMC (66.76). While the first four results were from the cathinone family in both cases, only the absolute search provided the correct molecule as a first hit. For 4-MMC, the absolute similarity search provided as match 4-MMC (100), 3-MMC (95.41), bk-MPA (91.23), 4-MEC (90.64) while the Euclidian similarity search returned 4-MMC (81.89), 4-MEC (68.02), 3-MMC (65.98), 3-CMC (65.73). While the first four results were from the cathinone family again, only the absolute search was able to provide 4-MMC with an HQI of 100 and 3-MMC as a second match. Therefore, absolute search was chosen as a default approach for all 1D ^1^H spectra. With this pre-validation, 1D ^1^H NMR can be used to identify but also to find substances close to the unknown one.

For AB-FUBINACA, the 1D ^19^F spectrum was compared with similarity and peak search. For similarity search with absolute algorithm, the first four matches were AB-FUBINACA (92.21), MDMB-FUBINACA (92.20), AMB-FUBINACA (91.87) and 3-FPM (91.46), while for the peak search only 3 molecules were returned with a LF fixed at 0.3 ppm: MDMB-FUBINACA (100), AMB-FUBINACA (100) and AB-FUBINACA (100). Therefore, peak search was found more efficient, probably because it also considers the multiplicity of the NMR signal.

For HSQC comparison, peak search was chosen by default because it is the only approach available in the software to compare HSQC maps. A first LF value needs to be set for NPS identification. In the 3-MMC / 4-MMC example, a single match should ideally be obtained. Such results were obtained with a LF of 0.1 ppm for ^1^H and 1 ppm for ^13^C. The next LF that must be chosen is the one for spectral homology comparison. This one must allow to obtain at least one another match for the 4-MMC. 3-MMC was found with an HQI of 100 if LF of 0.2 for ^1^H and 2 ppm for ^13^C.

Finally, for IR spectroscopy, comparison by peak for 3-MMC was tested but gave disappointing results. In fact, HQI was not calculated and the 4 first results were 3-MMC, APINACA, ADB-CHMINACA, 4-MMC. The second and the last matches were from cannabinoids family, so this type of comparison was excluded. Then absolute and Euclidian algorithm with similarity search were compared for the two pre validated molecules. For absolute classification of 3-MMC, the first matches were 3-MMC (87.36), 4-MMC (62.23), 4-MEC (60.02) and bk-MPA (52.91) while with Euclidian, 3-MMC (88.98), 4-MMC (60.54), 4-MEC (56.70) and bk-MPA (55.35) were obtained. For identification of 3-MMC, the largest difference between the two isomers was obtained with the Euclidian algorithm. The IR comparison of 4-MMC with absolute algorithm gave 4-MMC (100.00), 4-MEC (70.79), 4-EPD (62.56) and bk-MPA (60.76) as match while the Euclidian algorithm returned 4-MMC (100.00), 4-MEC (67.57) a homologous lateral chain of 4-MMC, 3-MMC (57.94) a positional isomer and 5F-ADB (57.10).To find NPS close to the unknown one, it seems that the Euclidian search is more correct since the found hits are very close to 4-MMC most of them have a substituent in para of their aromatic ring.

**The pre validation step allowed to find the best parameters for comparison. Thus, for the next steps the database comparison was performed with similarity search with Absolute algorithm for 1D ^1^H spectra, peak search for 1D ^19^F spectra, and similarity search with Euclidian algorithm for IR spectroscopy. In the case of HSQC, peak search with LF 0.1/1 ppm was chosen for identification and at least 0.2/2 ppm for elucidation**.

S5: 1D ^1^H 3-MMC peak search


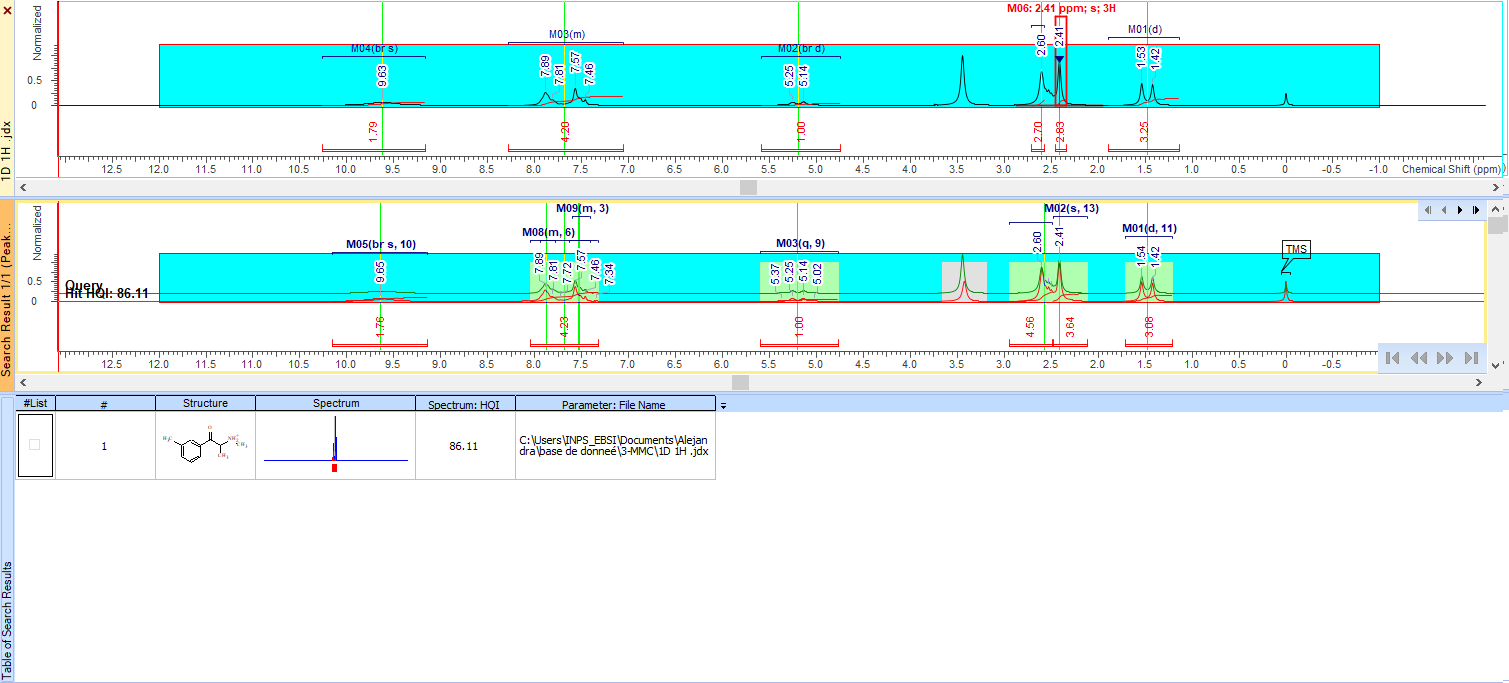


S5: 1D ^1^H 4-MMC peak search


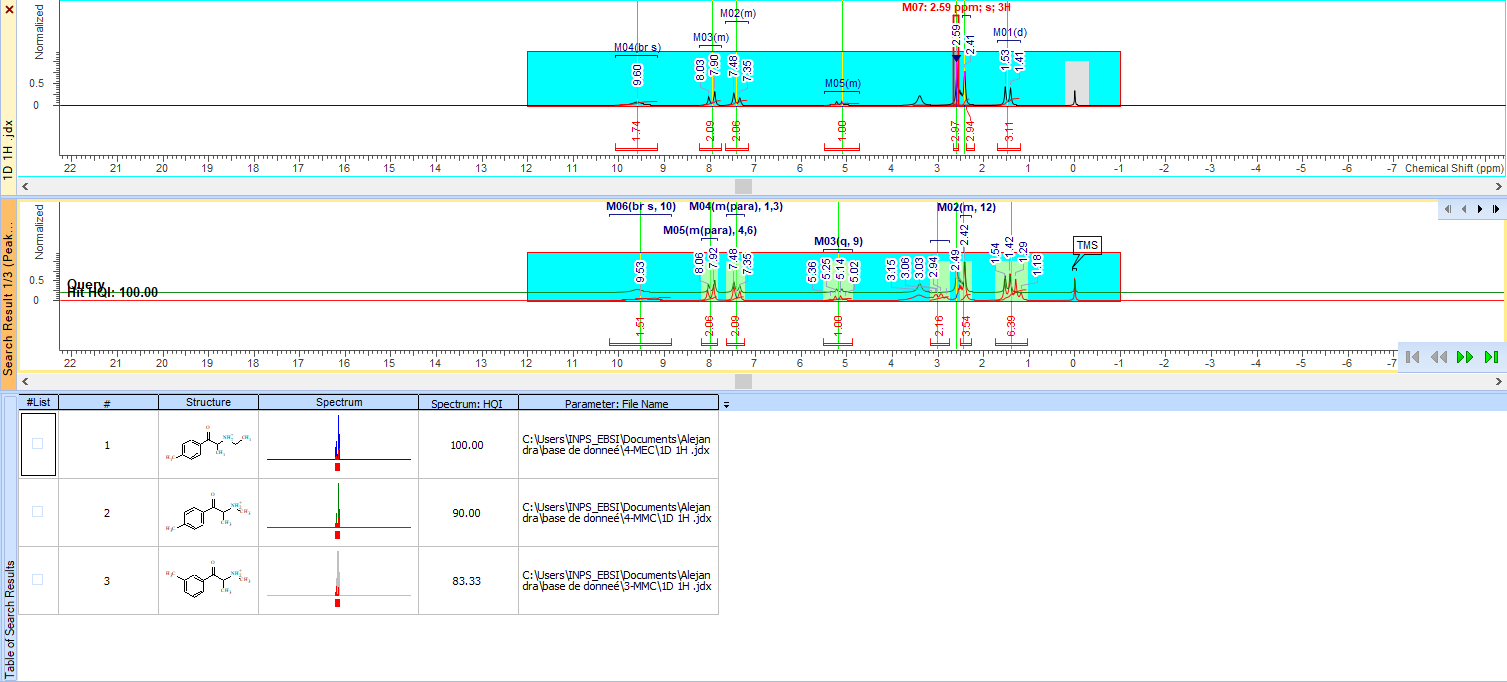


S5: 1D ^1^H 3-MMC absolute algorithm


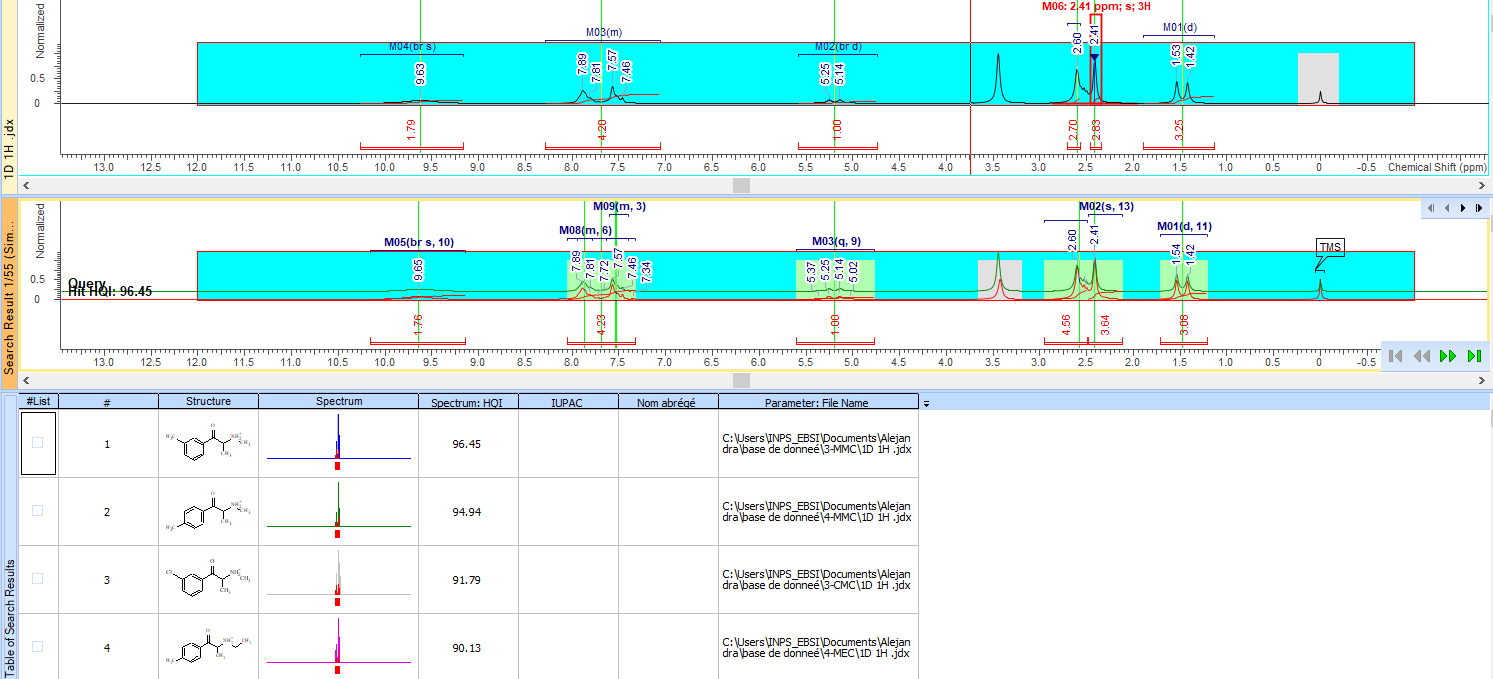


S5: 1D ^1^H 3-MMC euclidian algorithm


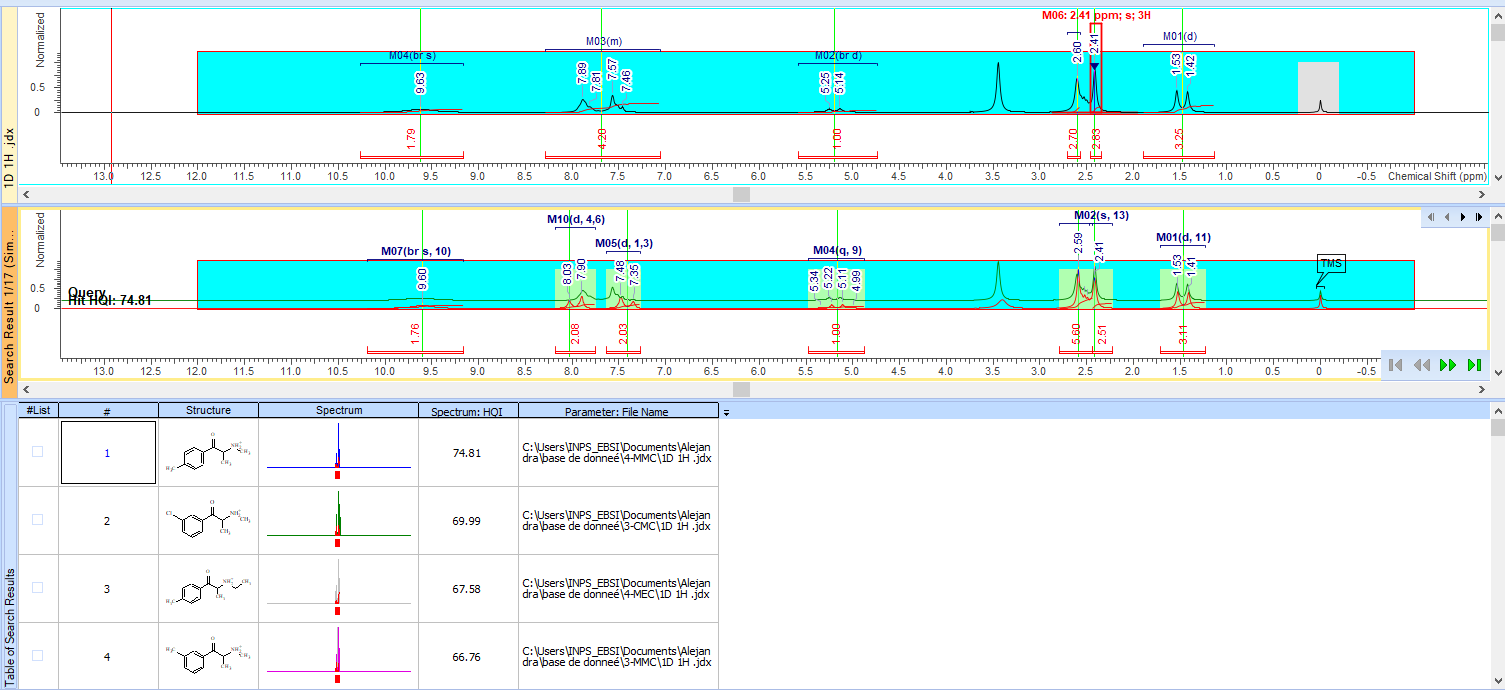


S5: 1D ^1^H 4-MMC absolute algorithm


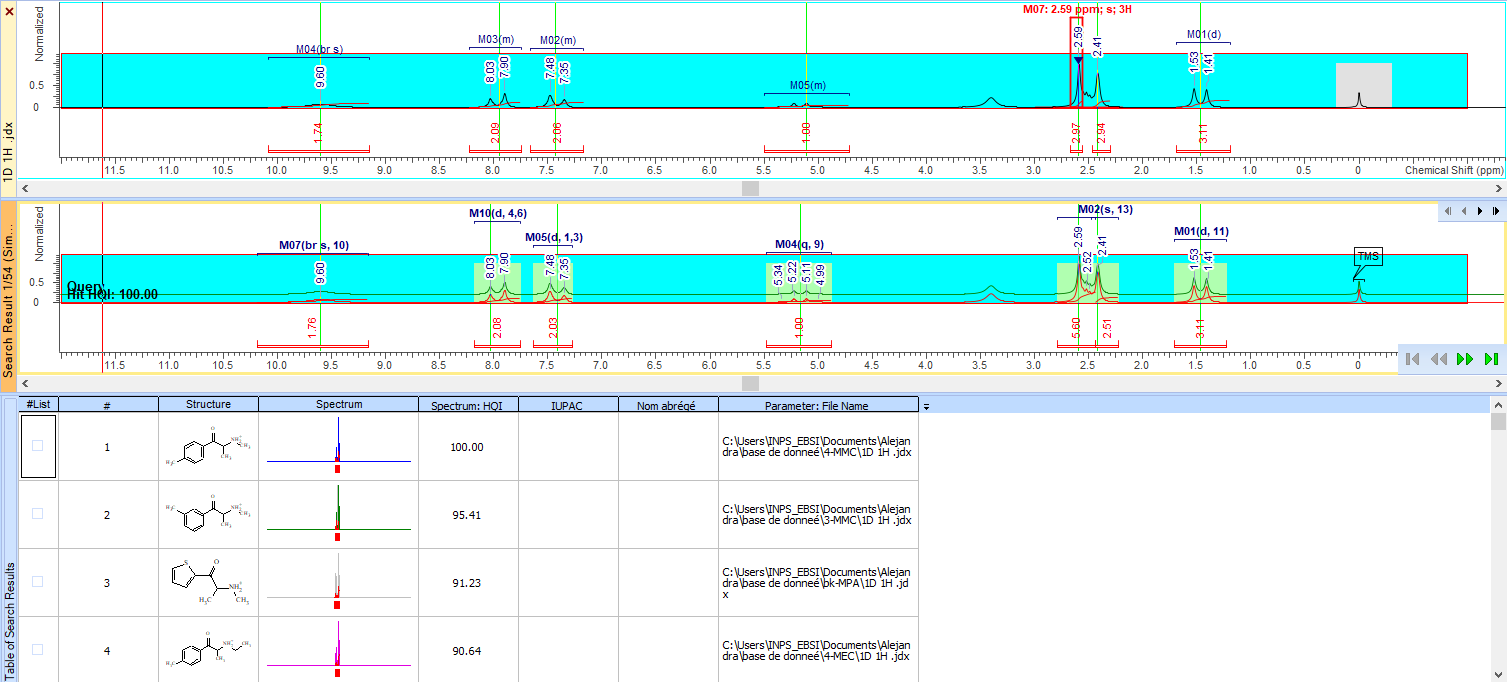


S5: 1D ^1^H 4-MMC euclidian algorithm


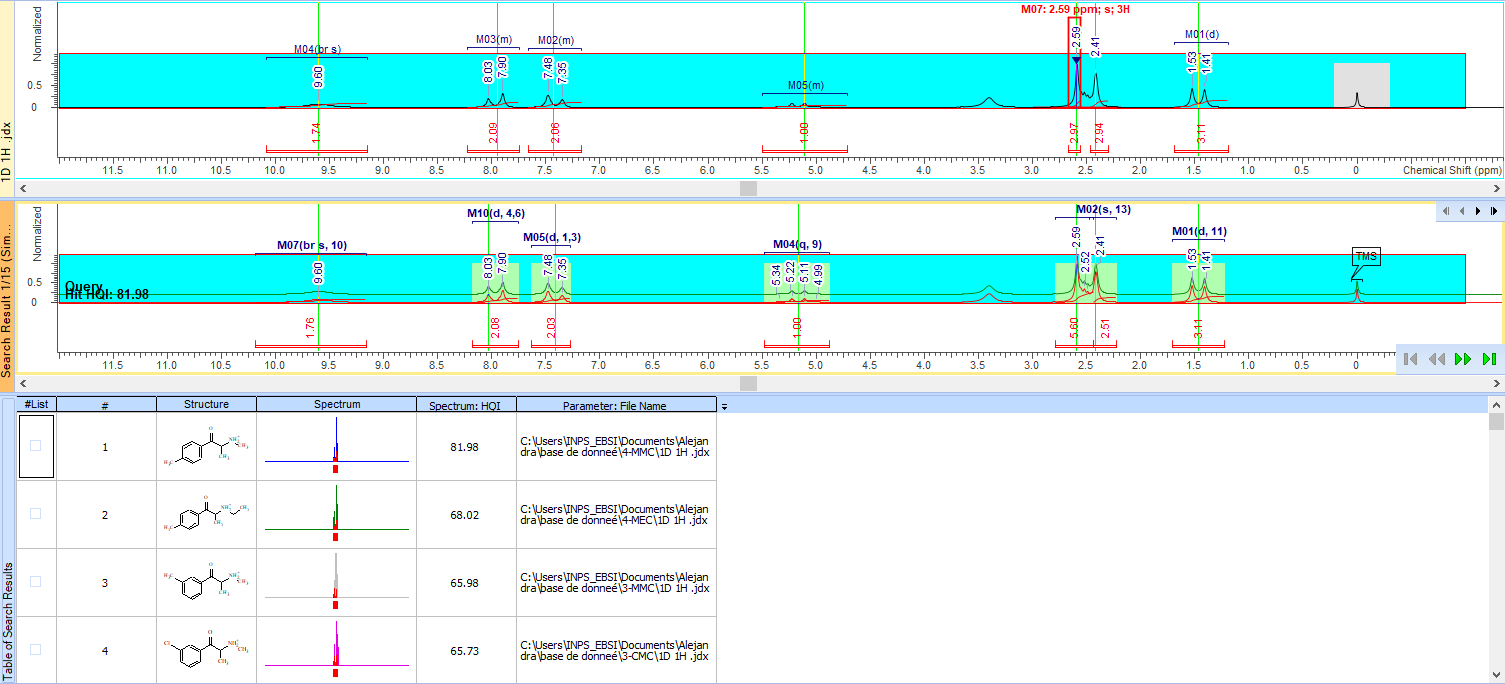


S5: 1D ^19^F AB-FUBINACA similarity search


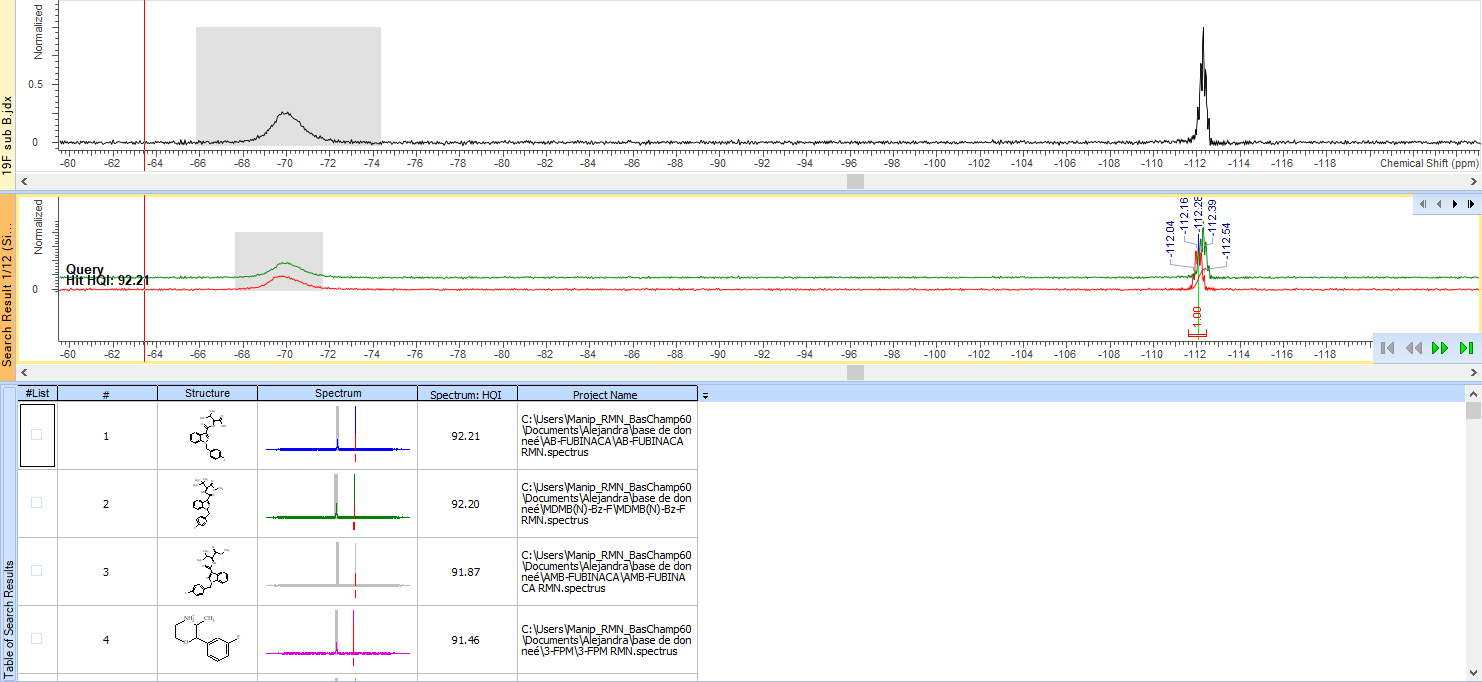


S5: 1D ^19^F AB-FUBINACA peak search


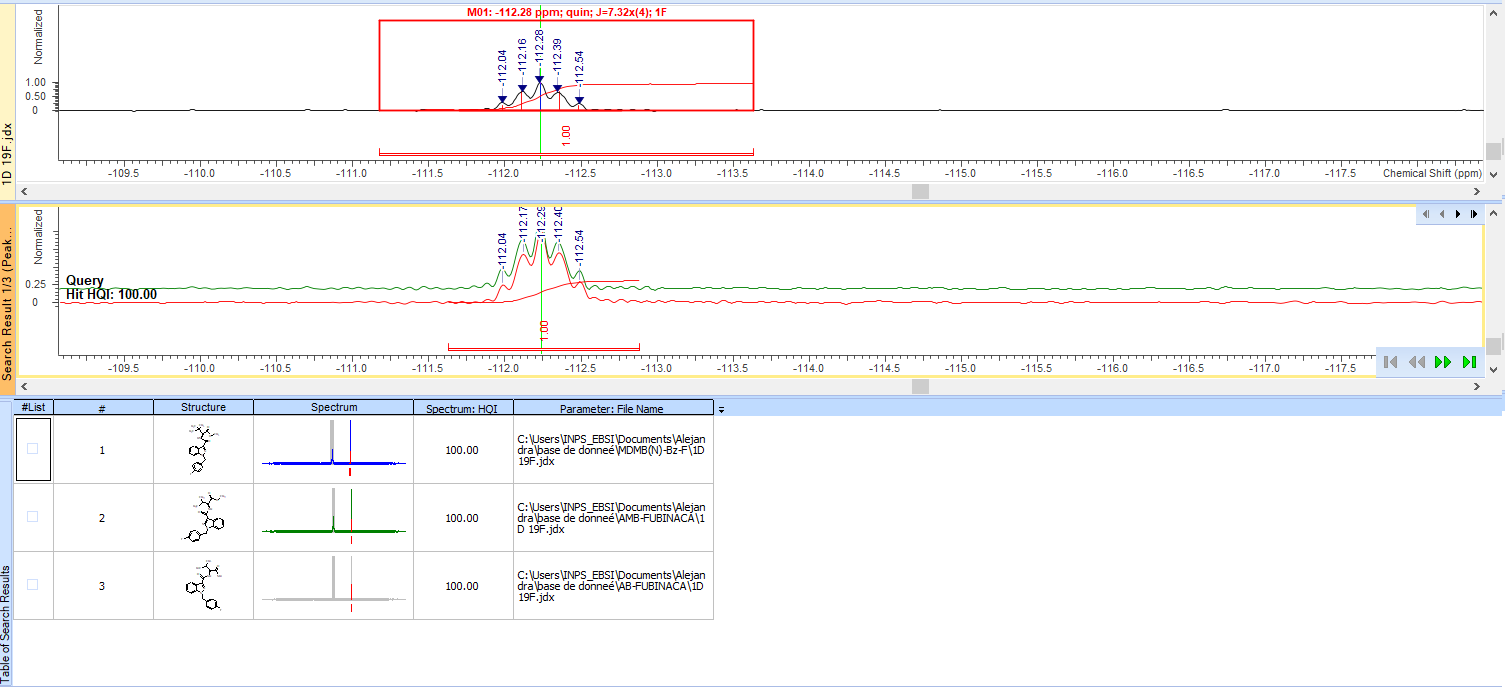


S5: HSQC 3-MMC identification


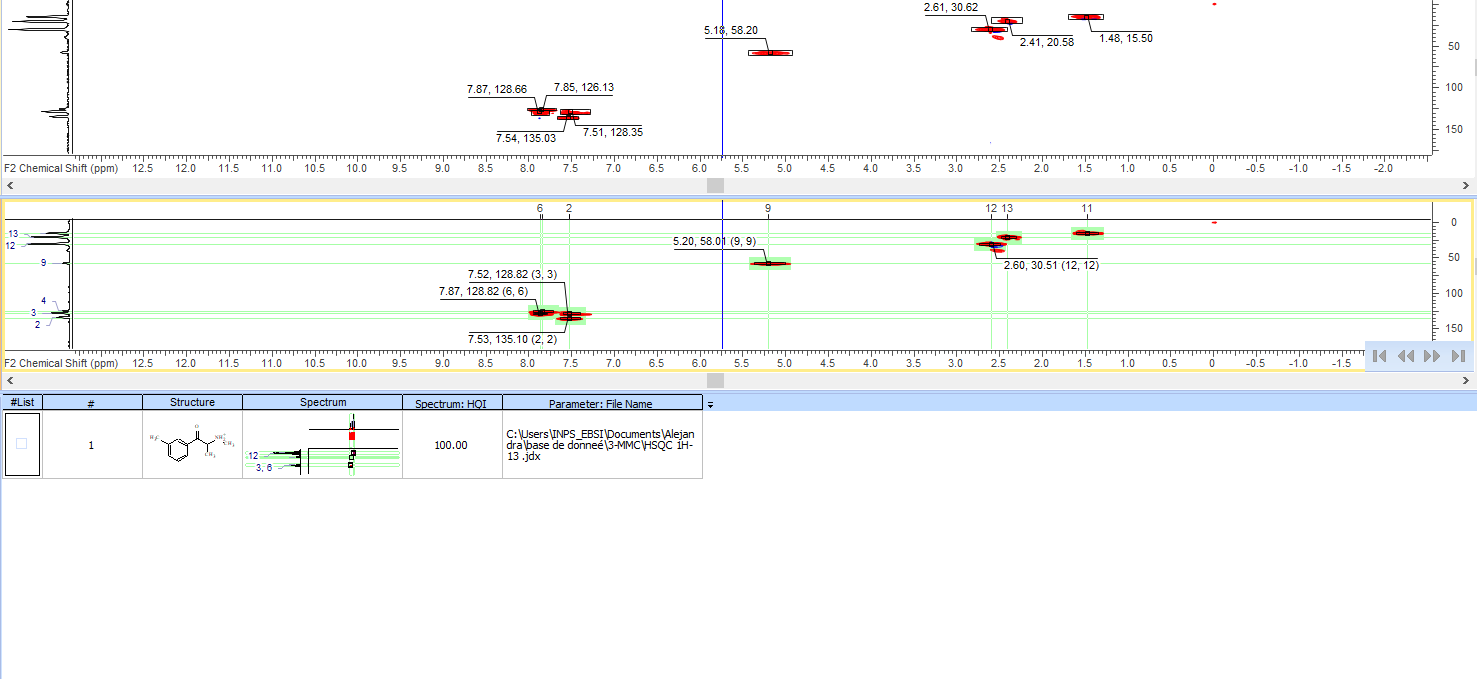


S5: HSQC 4-MMC identification


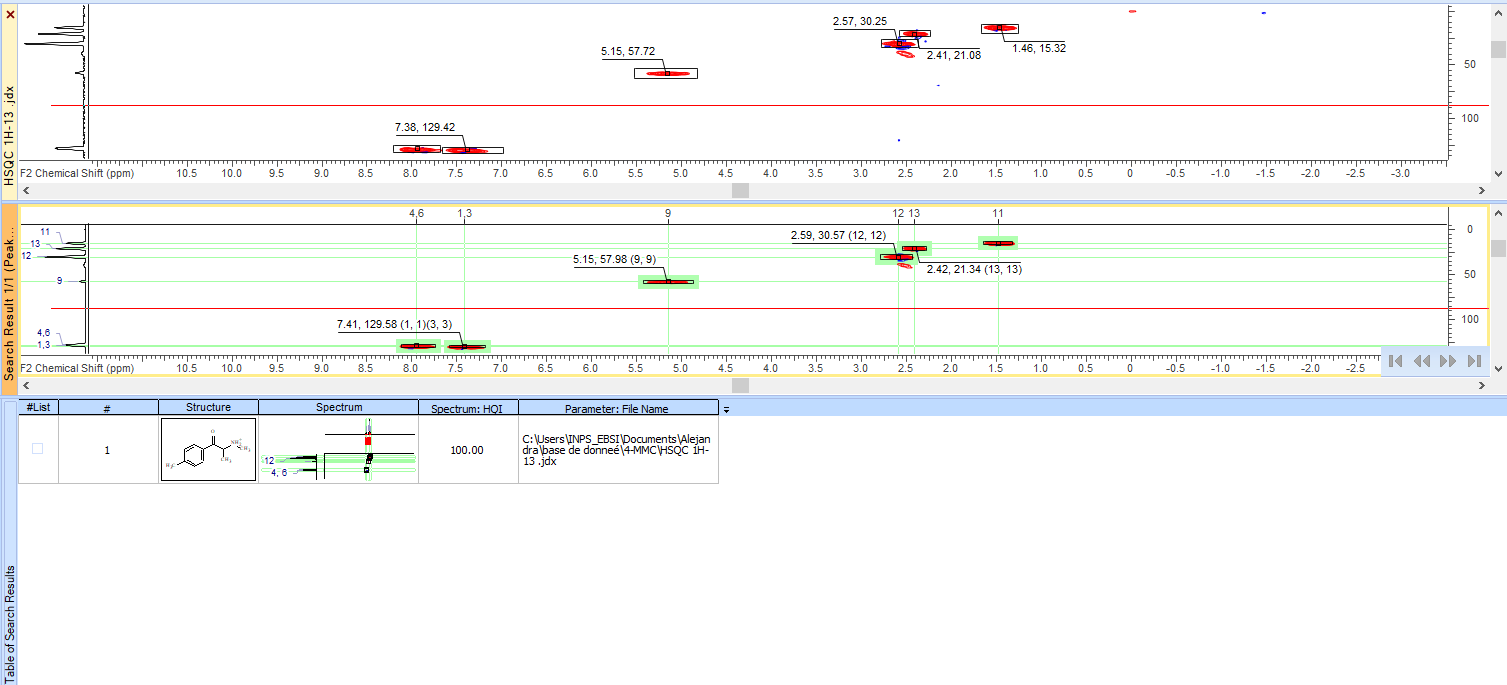


S5: HSQC 4-MMC spectral homology


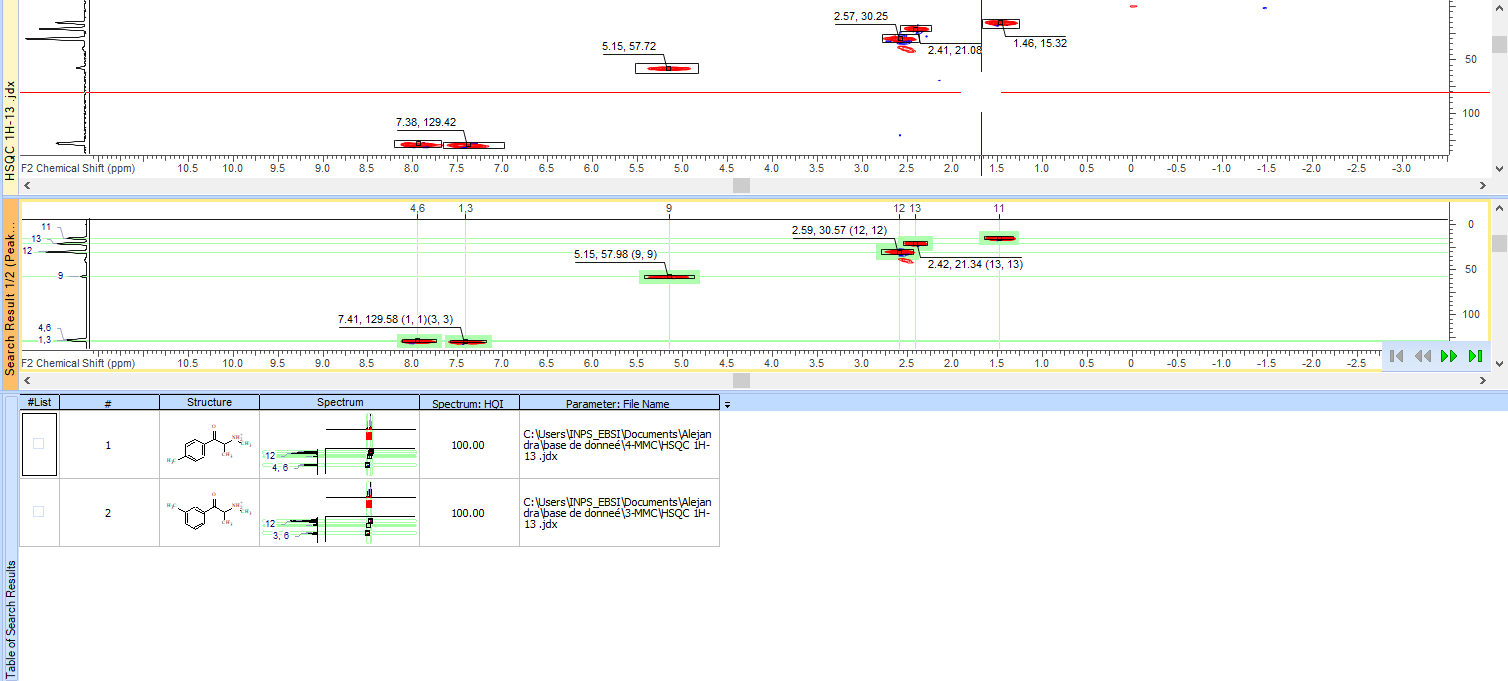


S5: IR 3-MMC peak search


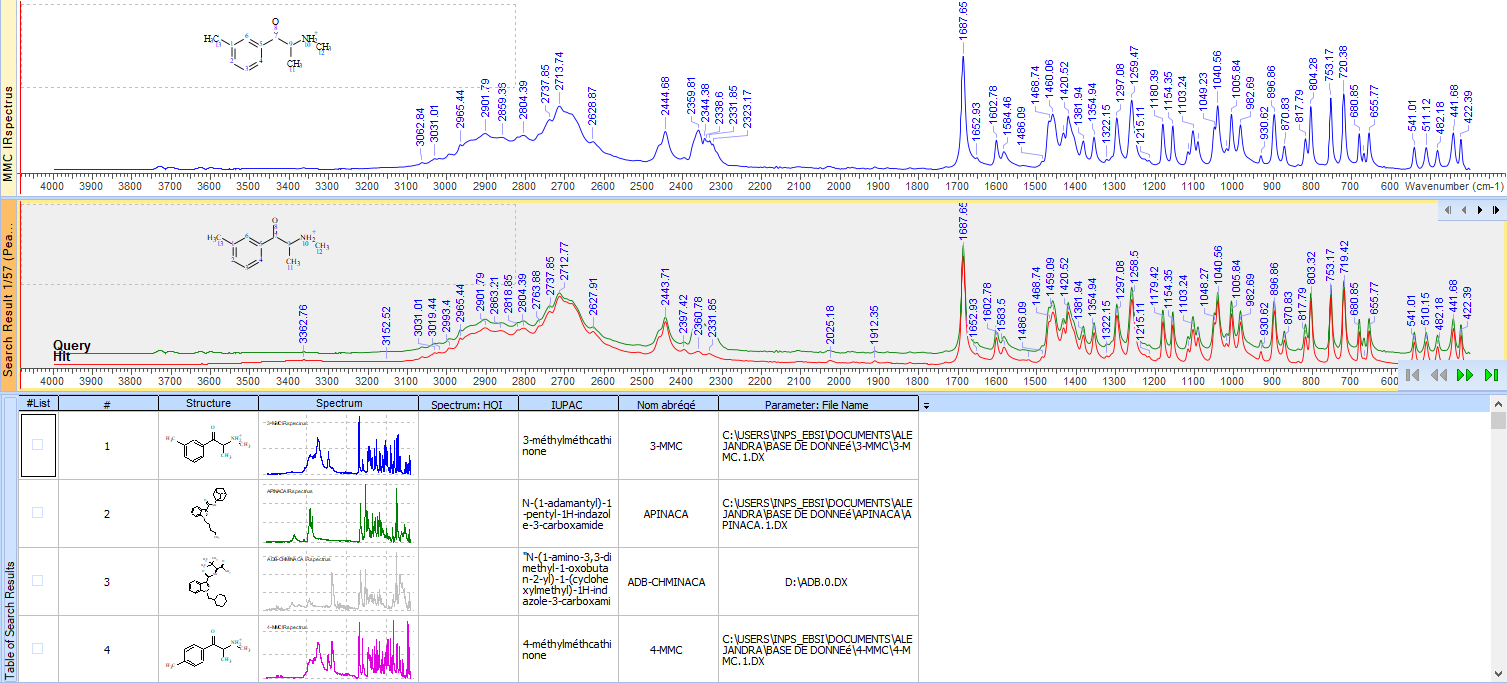


S5: IR 3-MMC absolute algorithm


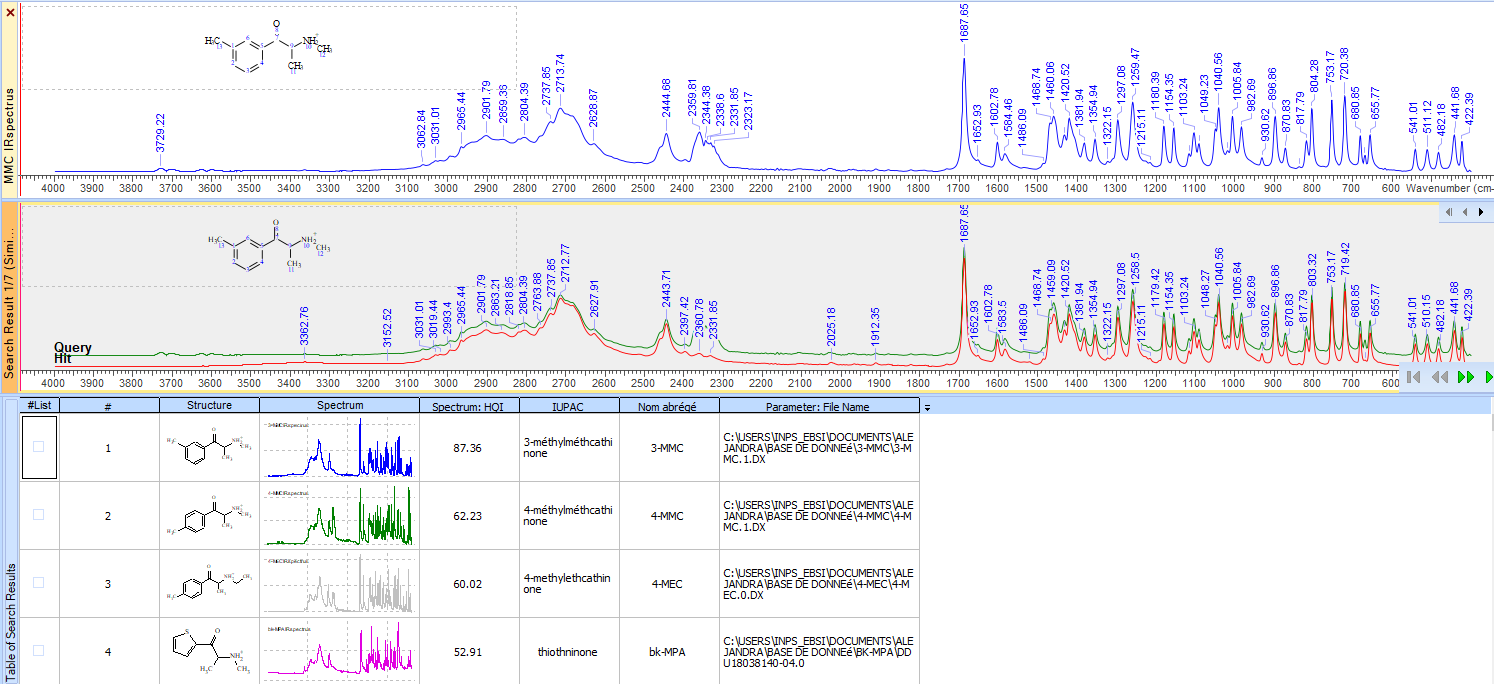


S5: IR 3-MMC euclidian algorithm


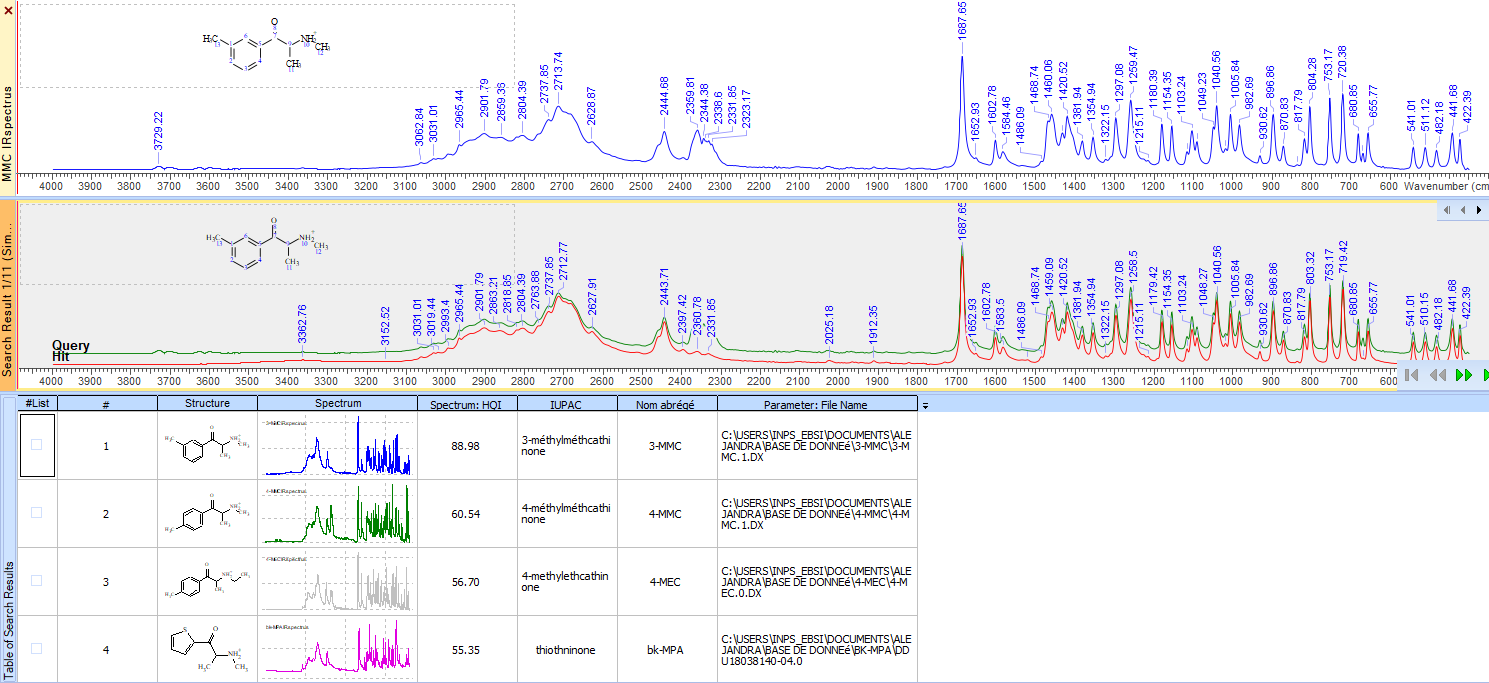


S5: IR 4-MMC peak search


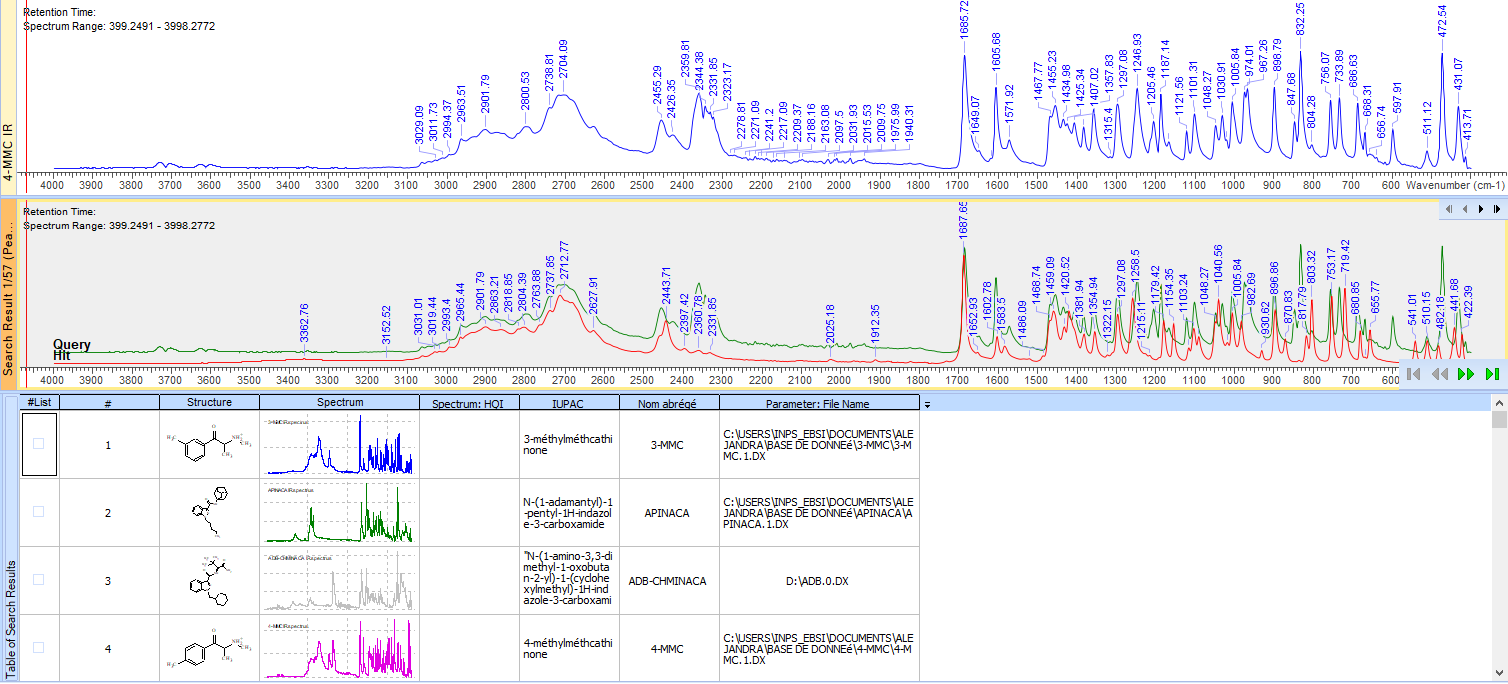


S5: IR 4-MMC absolute algorithm


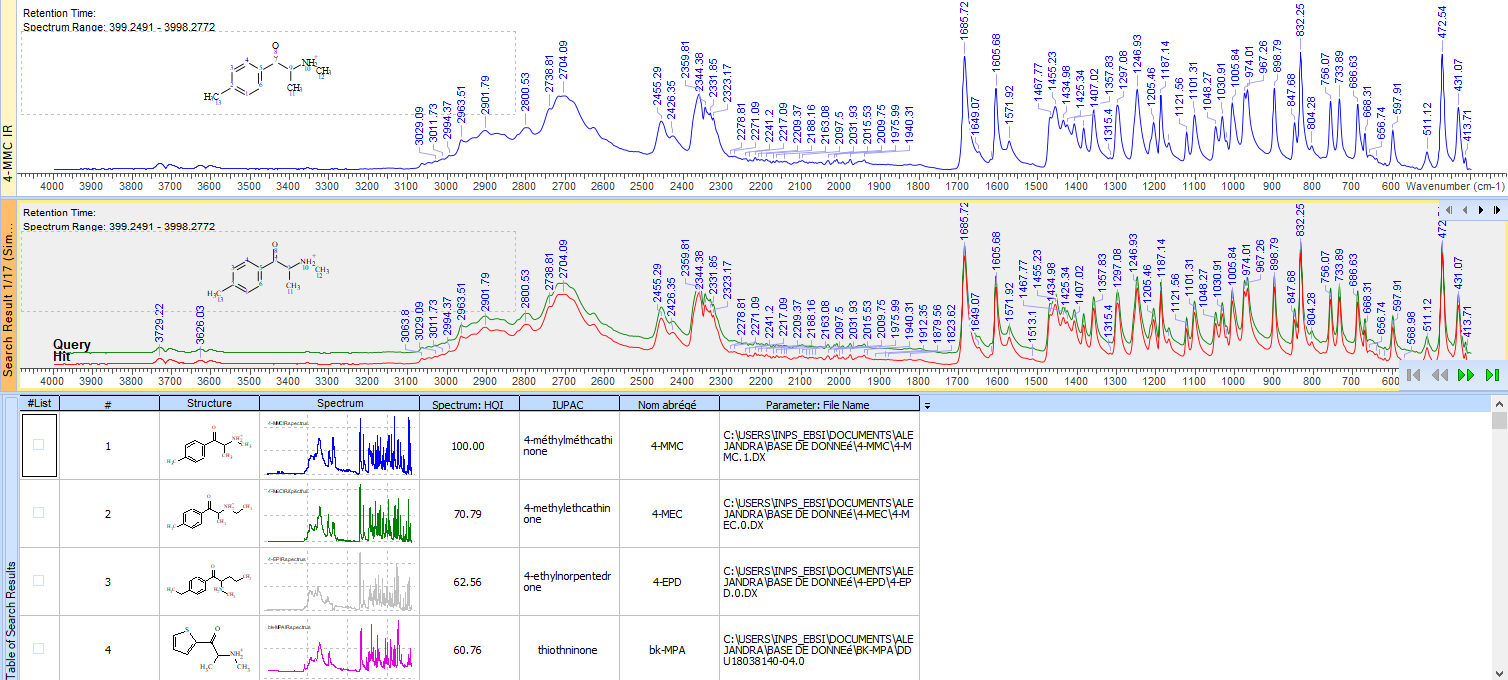


S5: IR 4-MMC euclidian algorithm


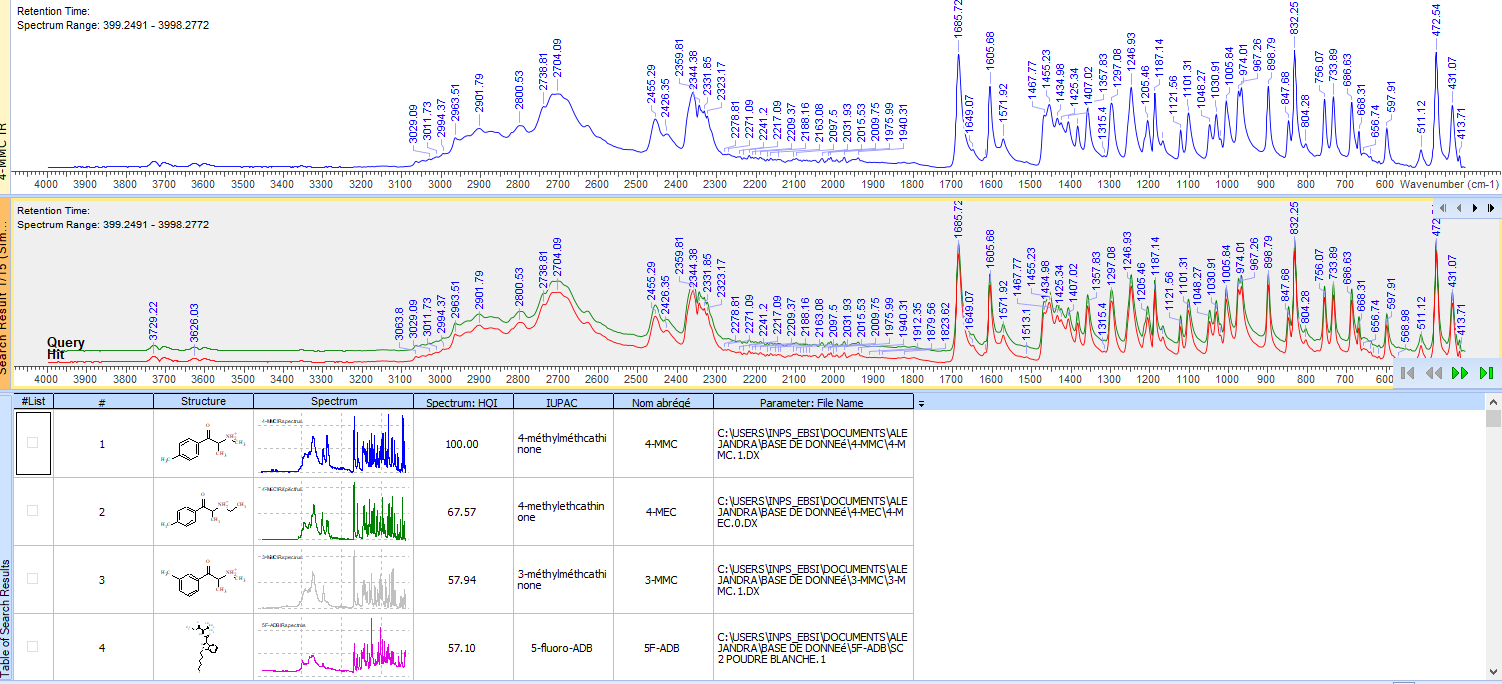


S6: 1D ^19^F substance n°1


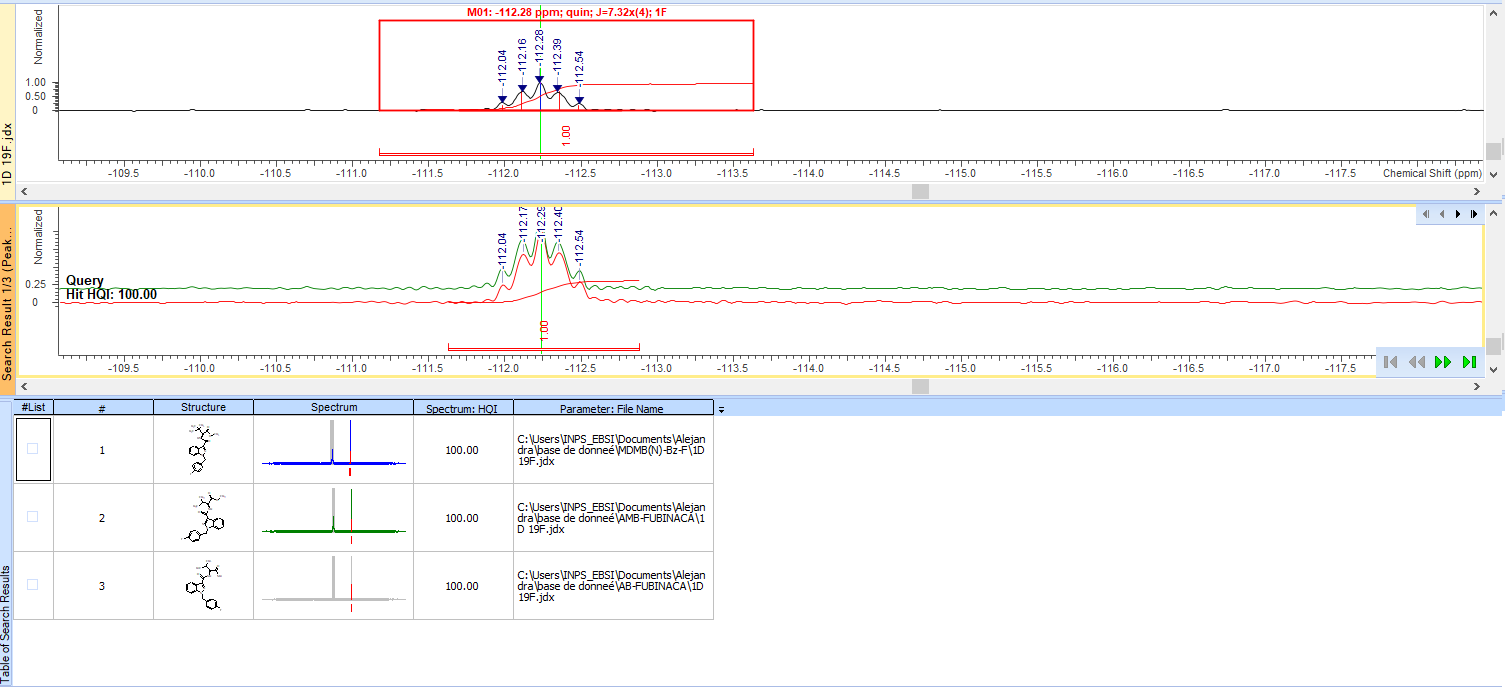


S6: 2D HSQC substance n°1


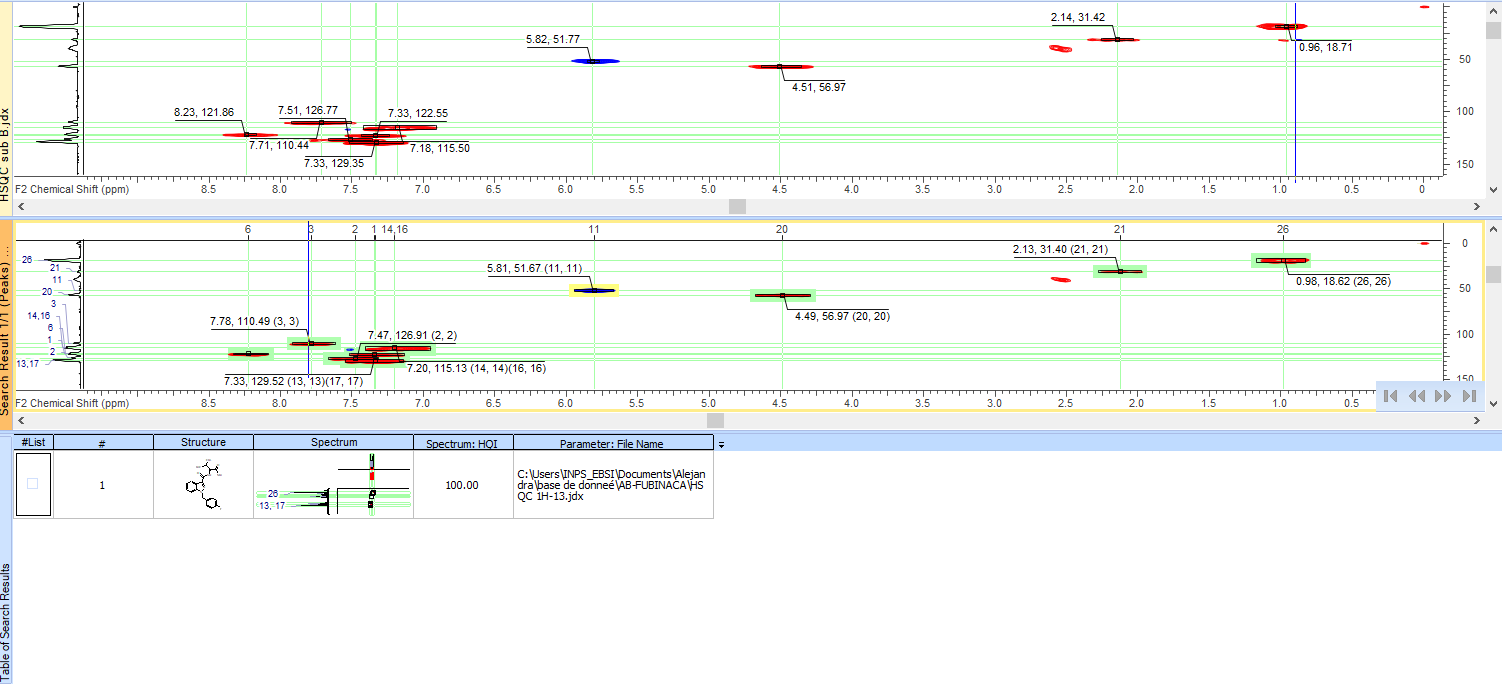


S6: IR substance n°1


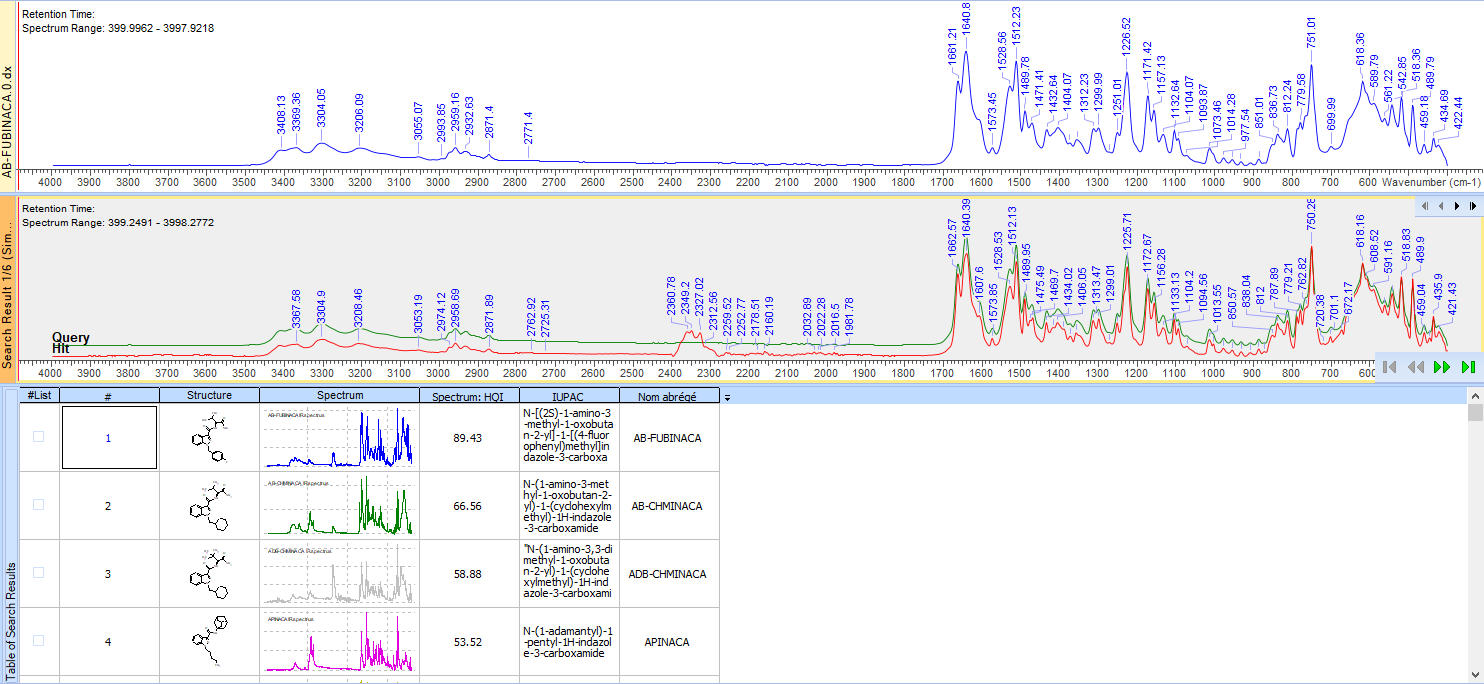


S6 : Identification substance 1 at 700MHz

1D ^1^H


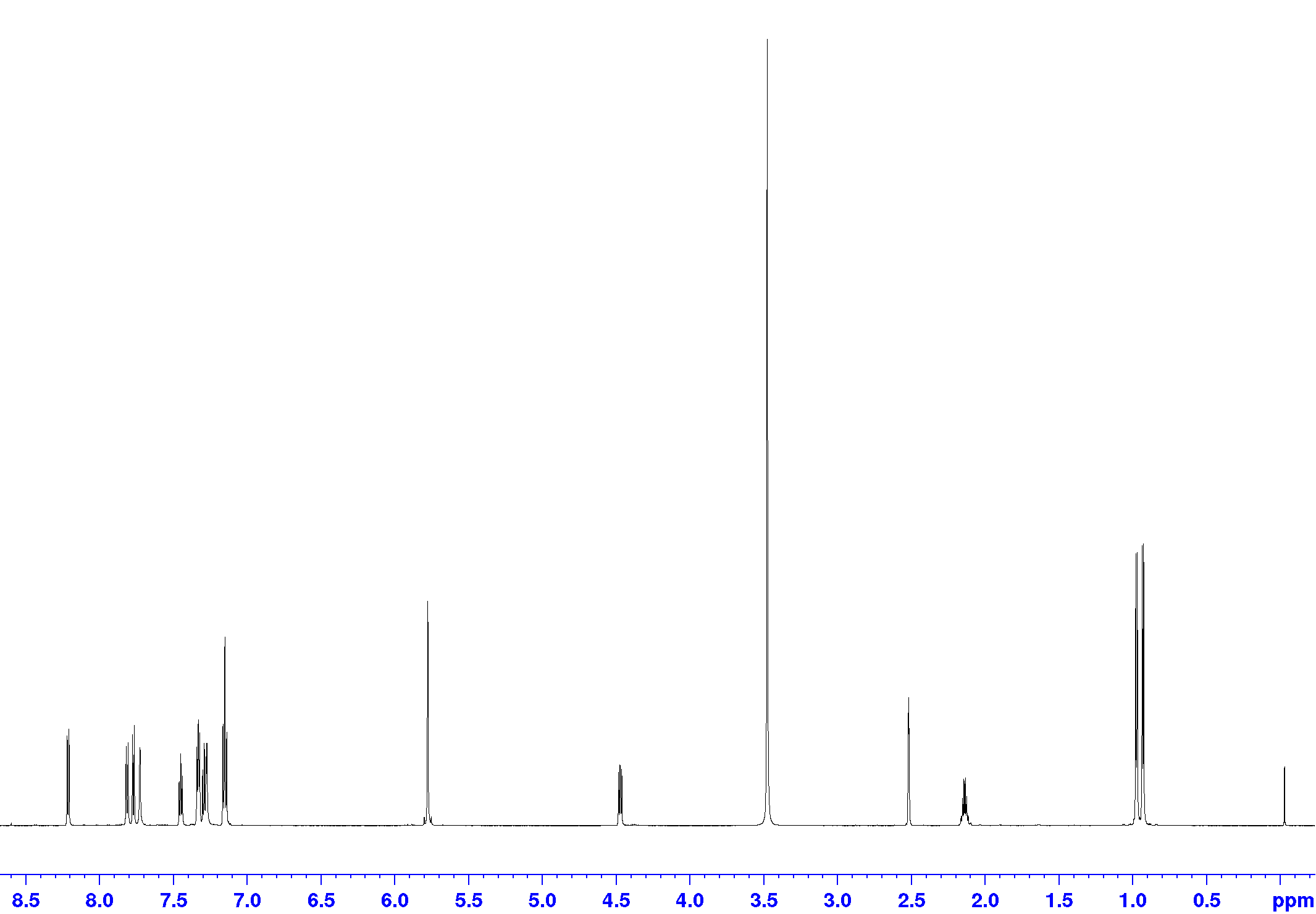


^1^H-^1^H COSY


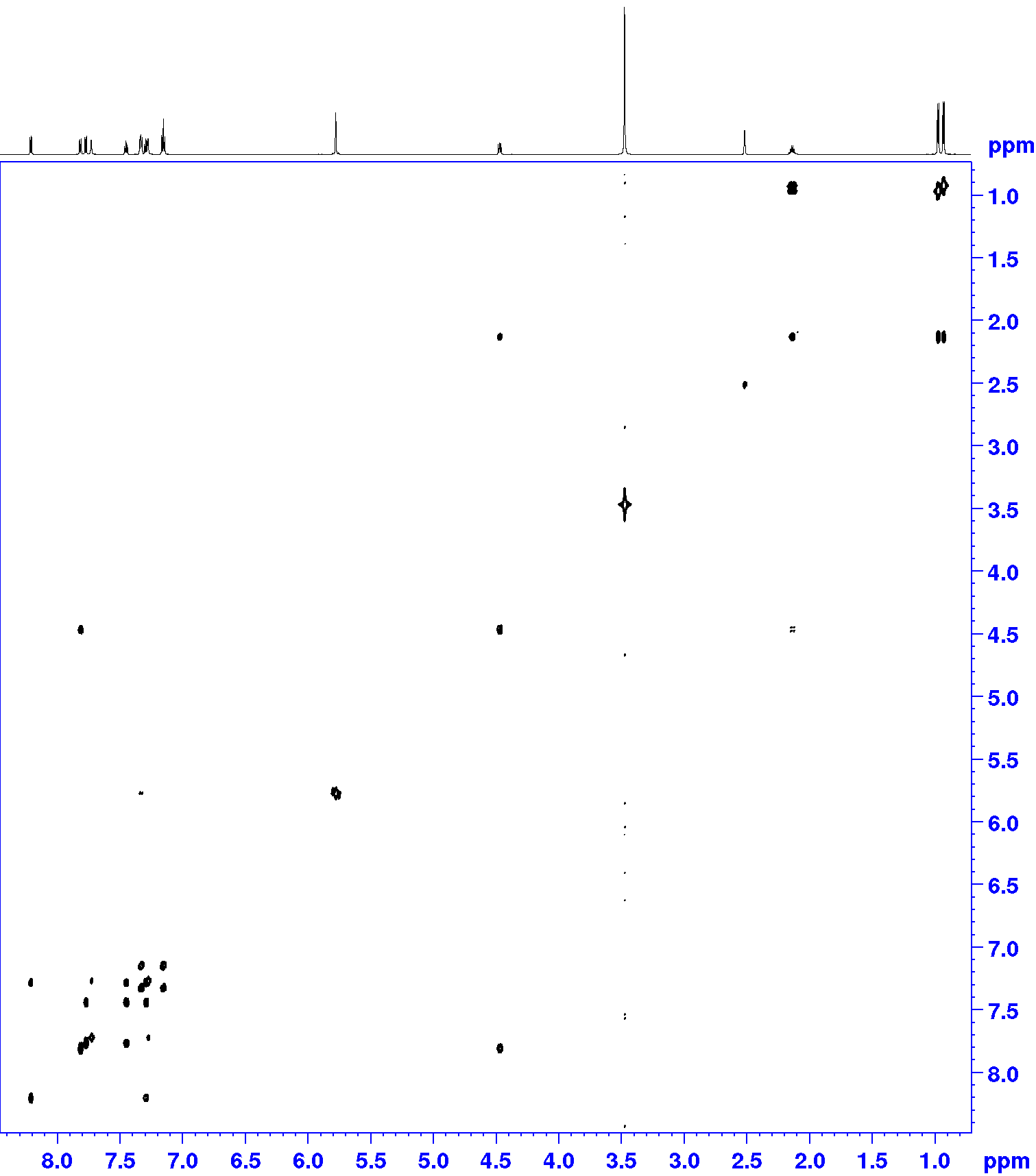


^1^H-^13^C HSQC


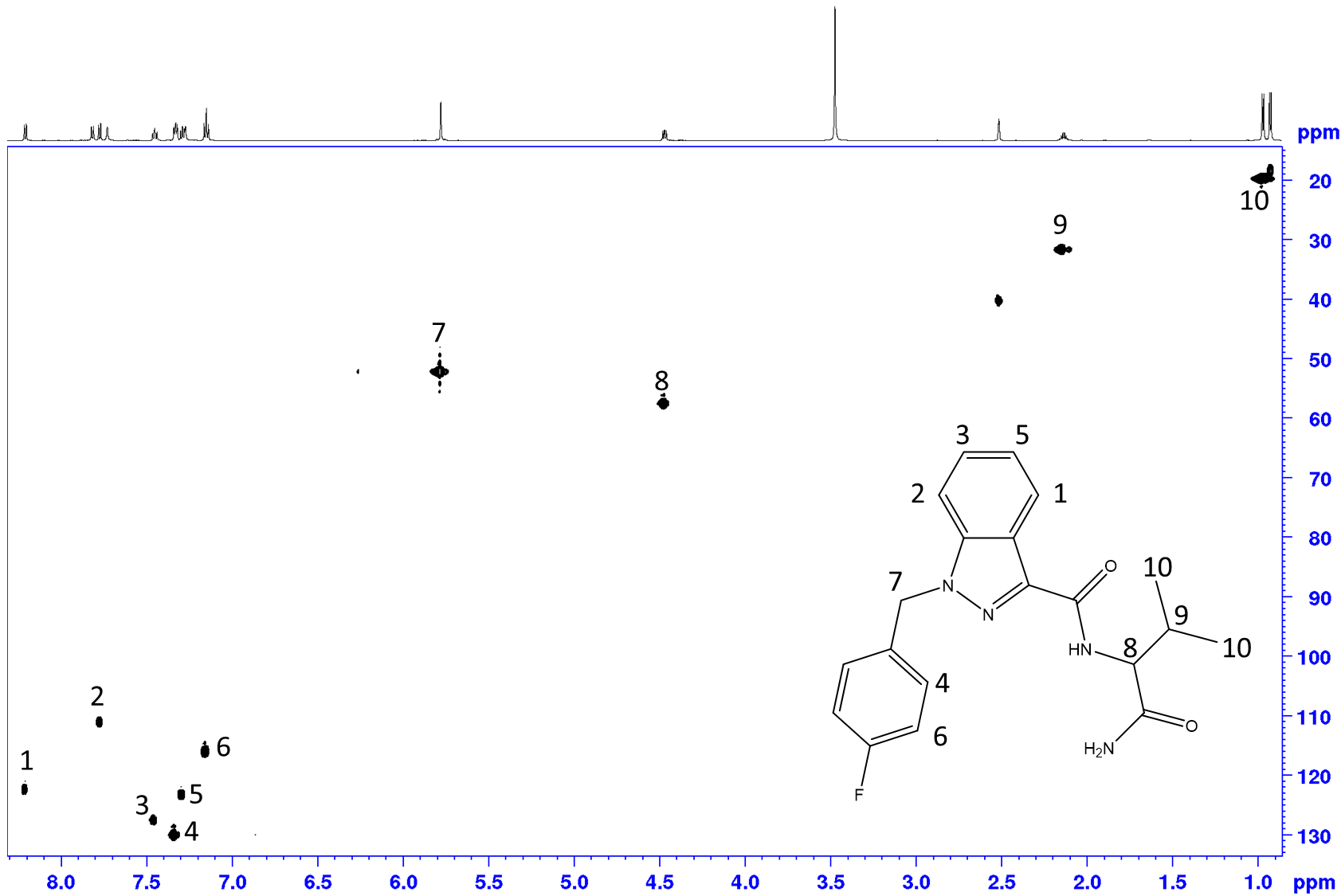


S7: 2D HSQC substance n°2


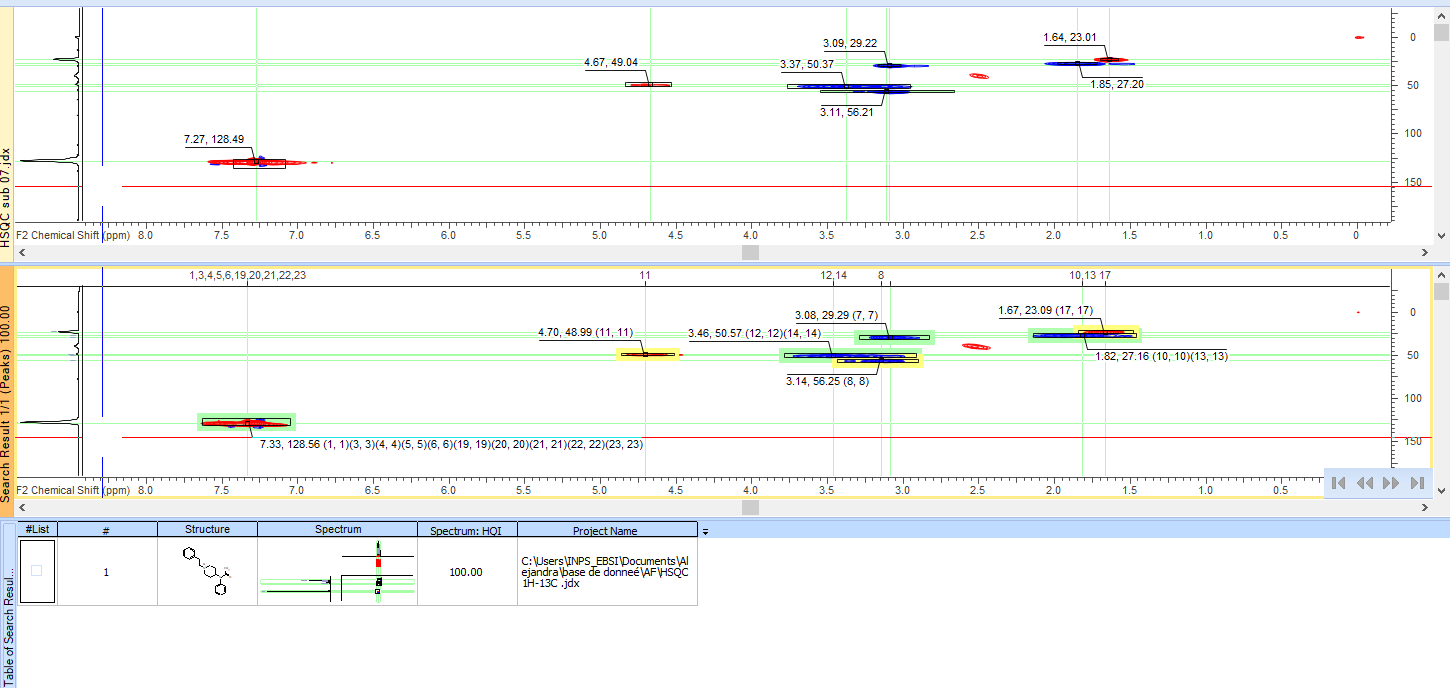


S7: IR substance n°2


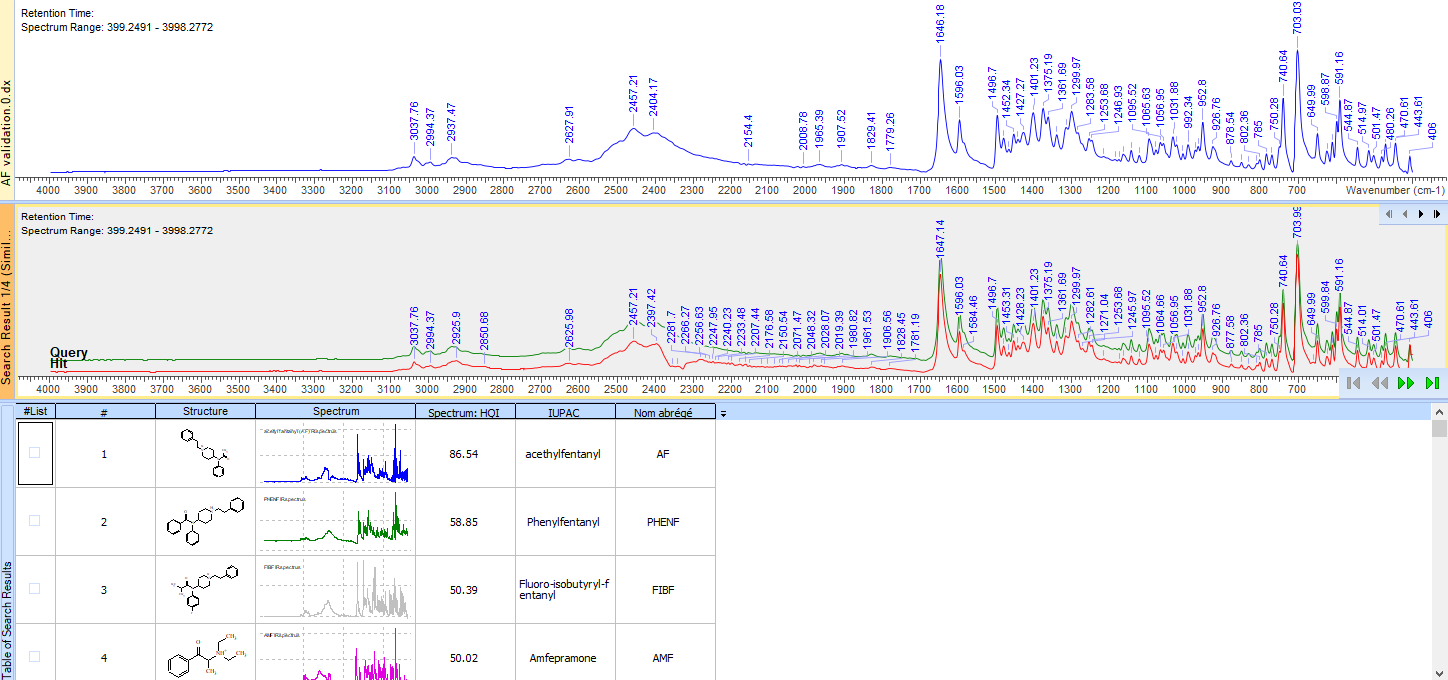


S7: Identification substance 2 at 700MHz

1D ^1^H


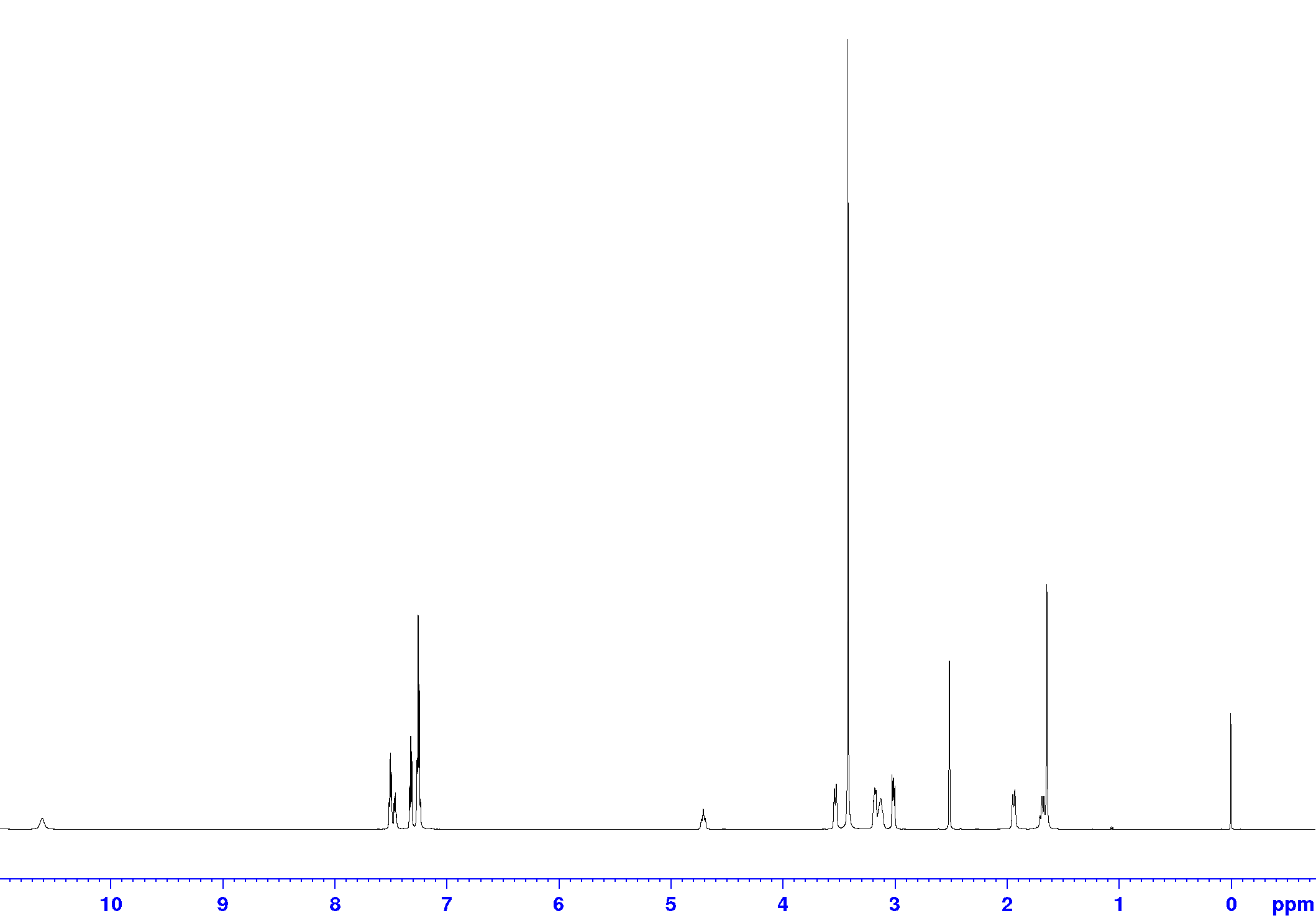


^1^H-^1^H COSY


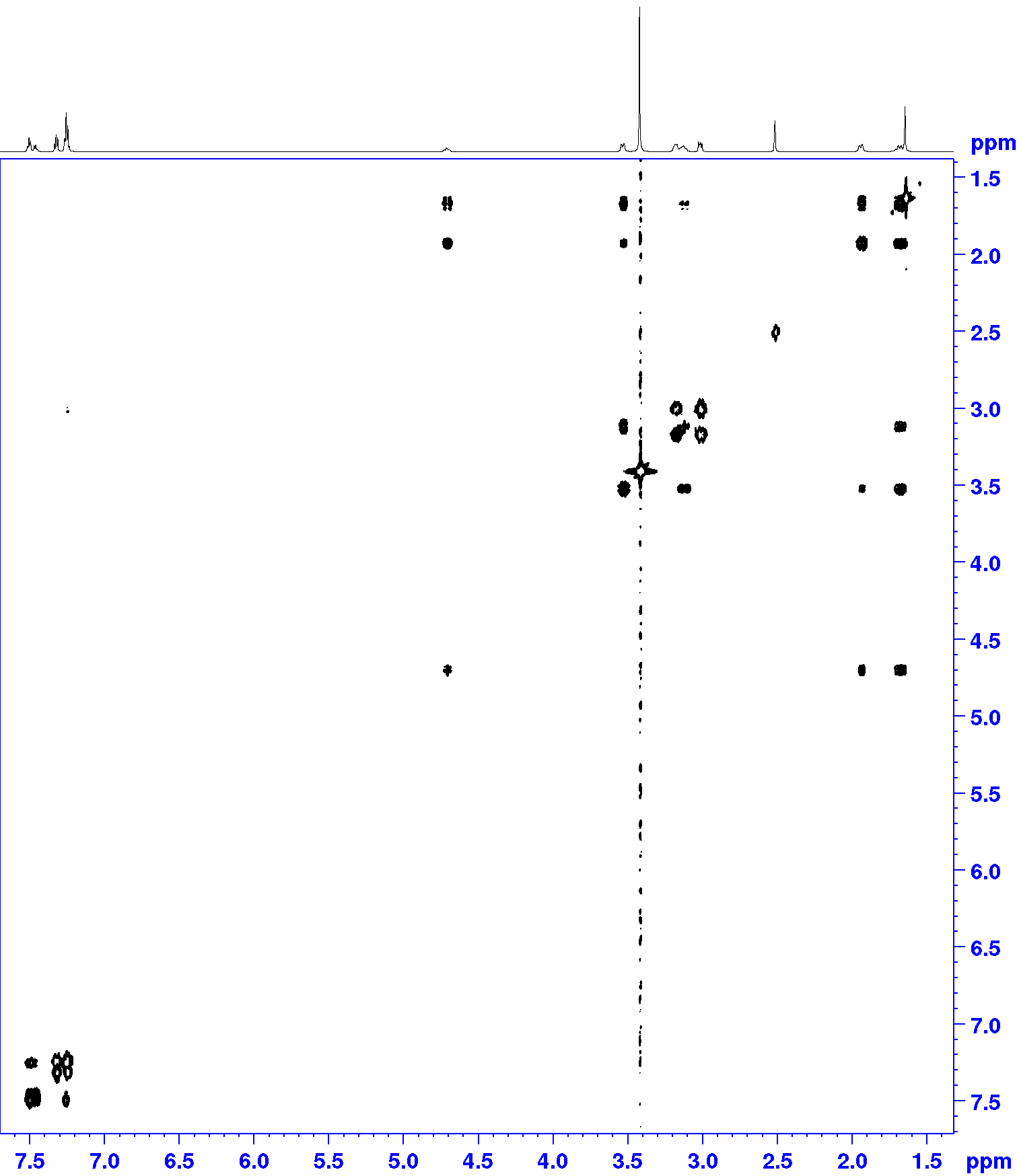


^1^H-^13^C HSQC


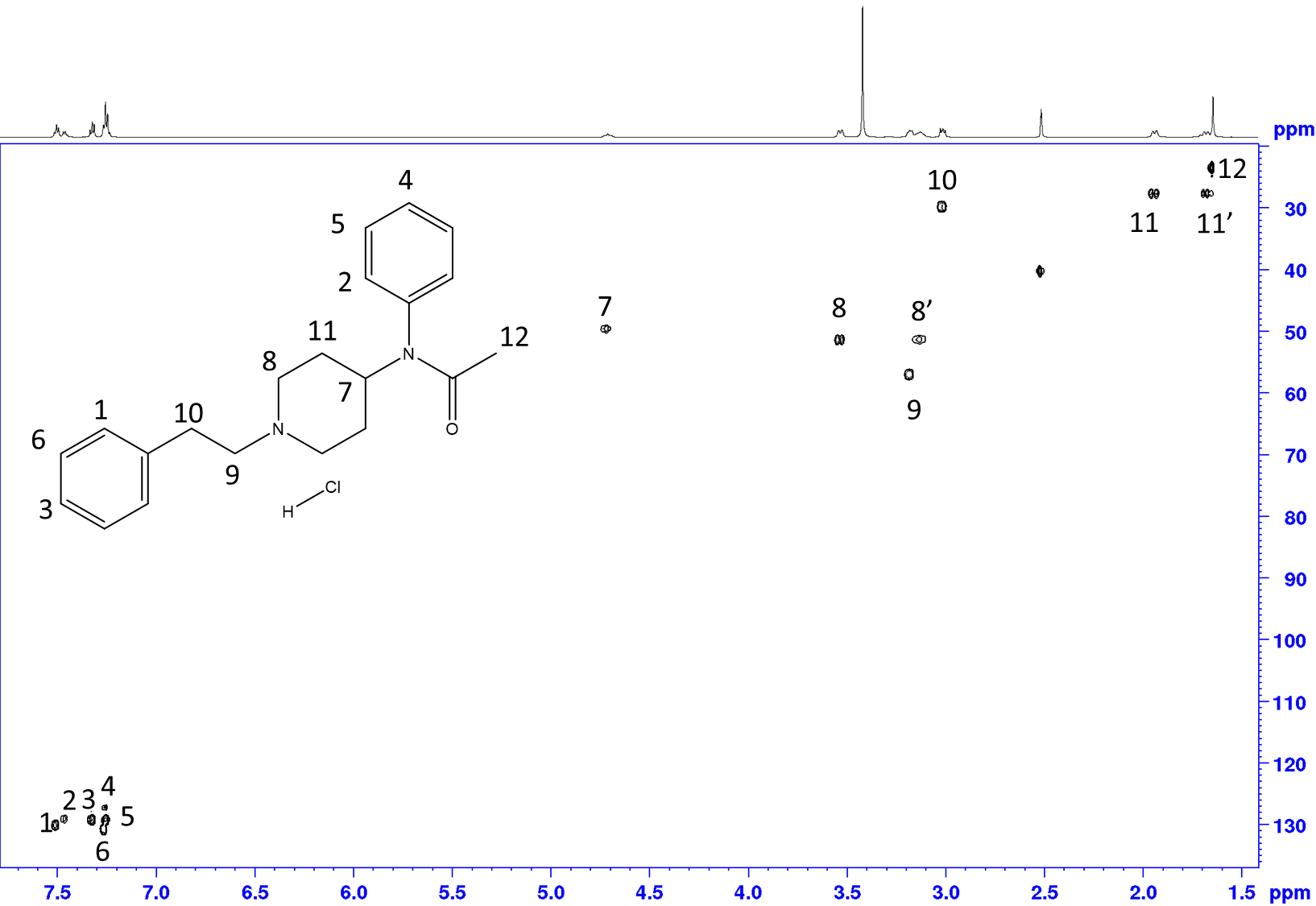


S8: 2D HSQC substance n°3


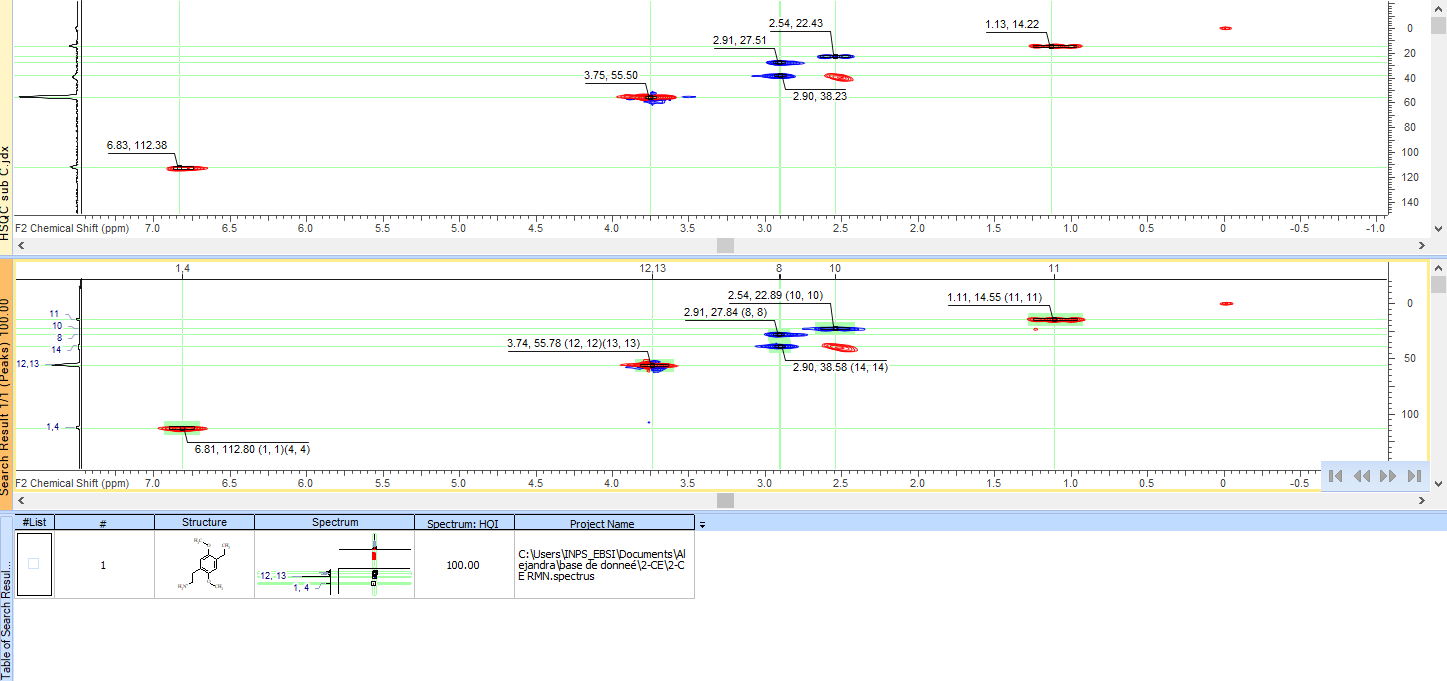


S8: 1D ^1^H substance n°3


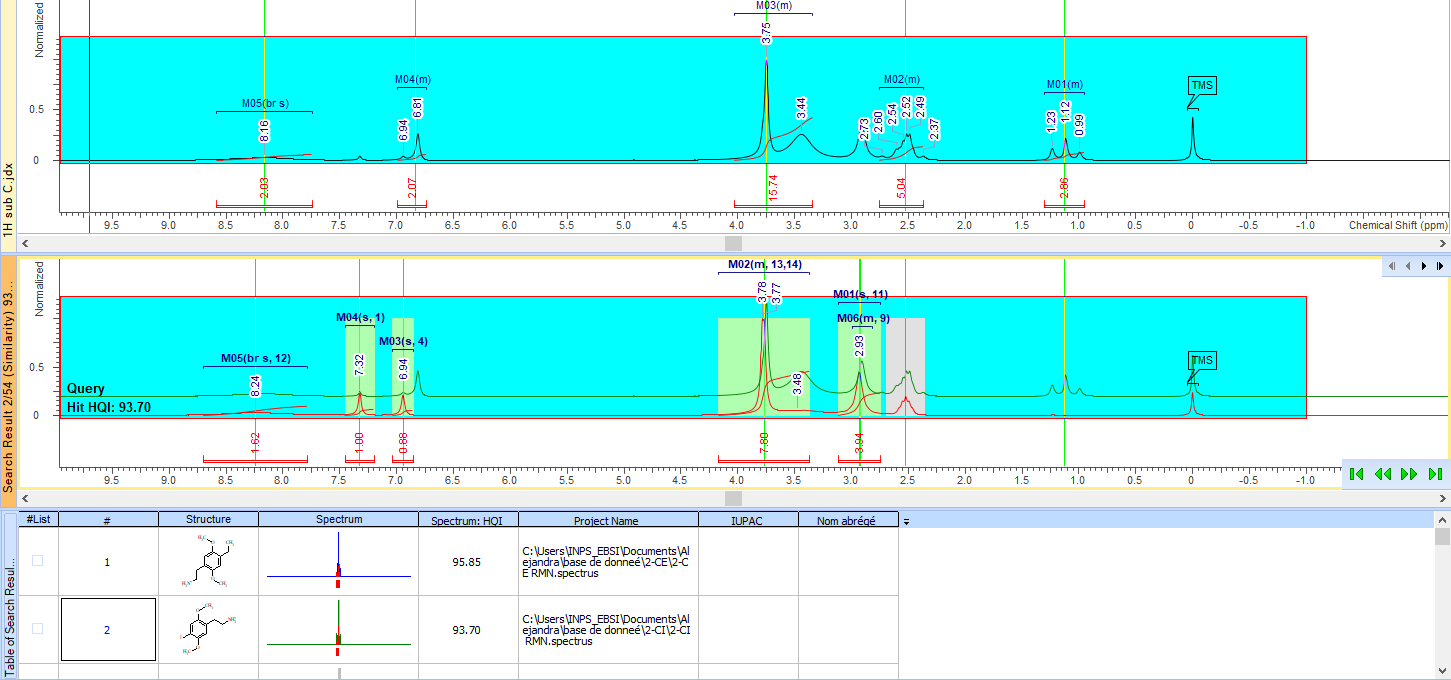


S8: Identification substance 3 at 700MHz

1D ^1^H NMR comparison with pure samples


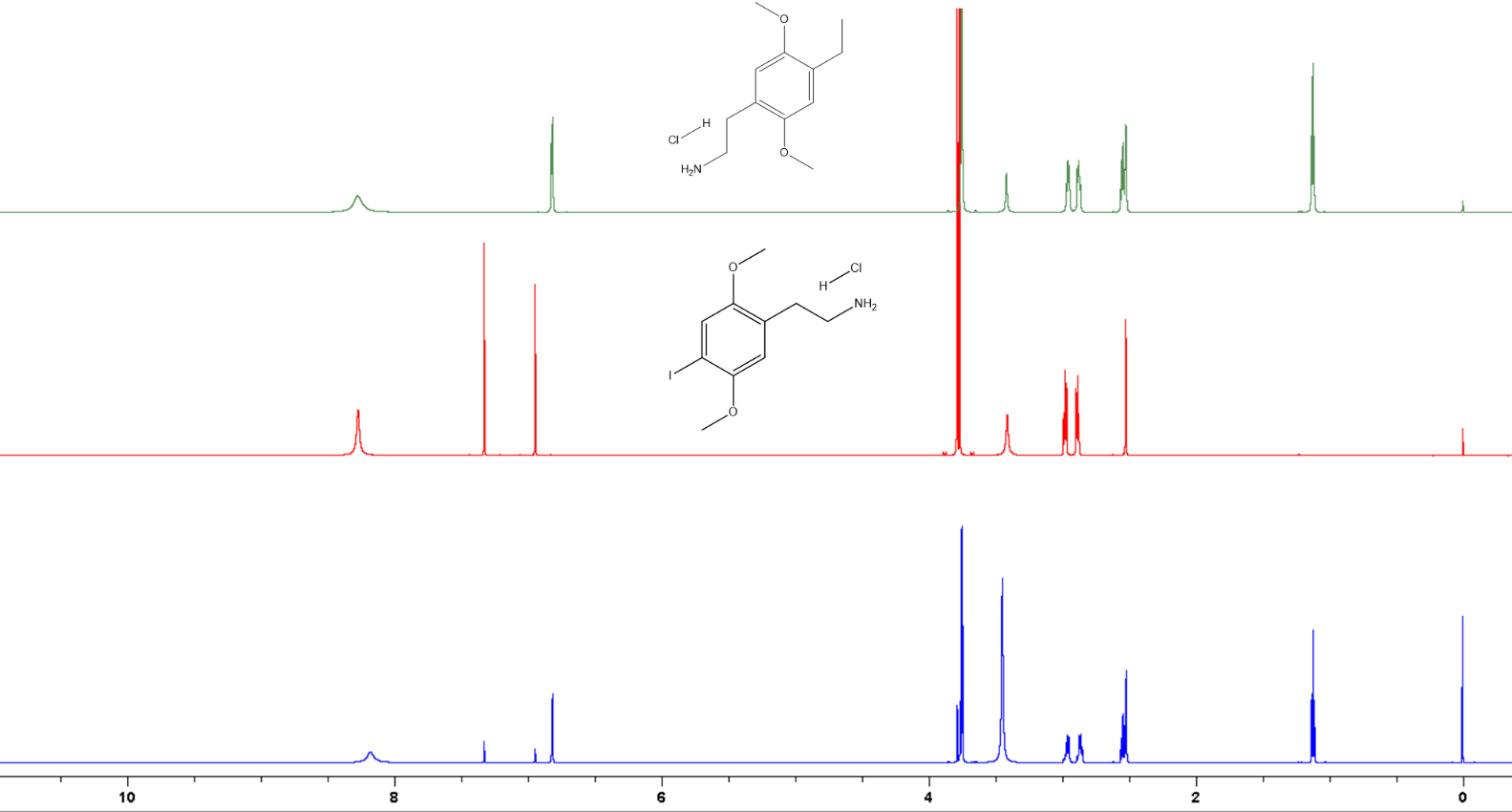


S9: 2D HSQC identification substance n°4


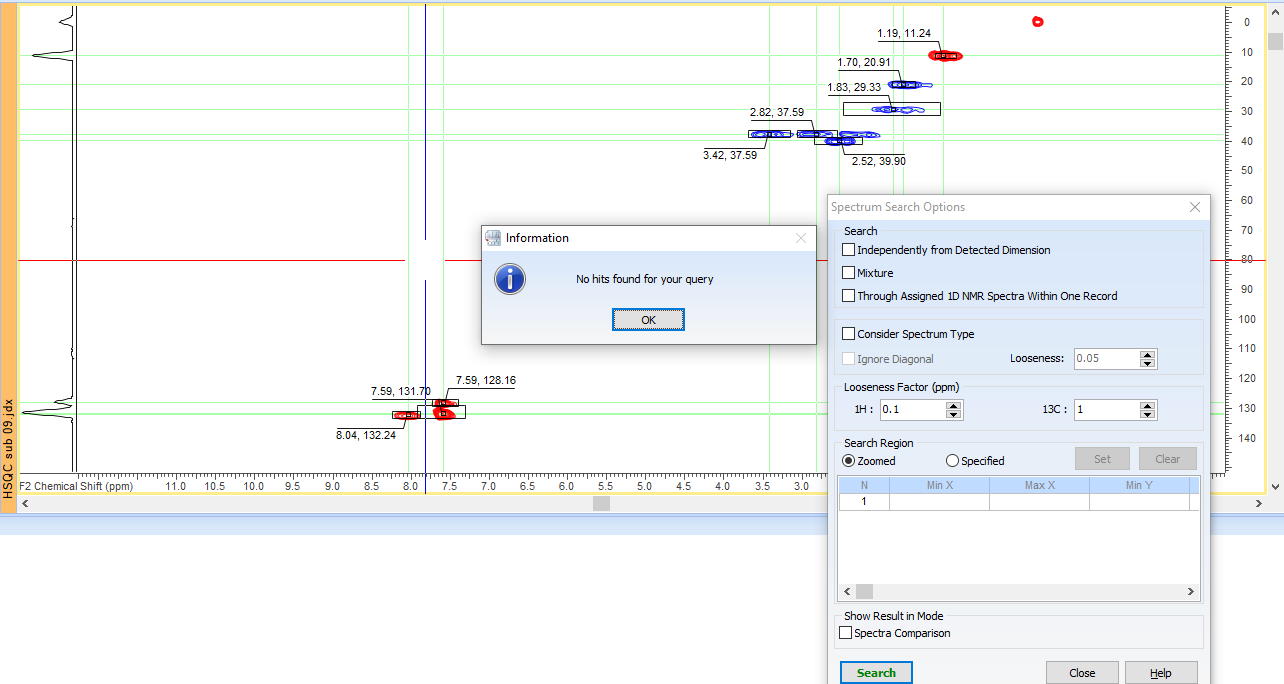


S9: 2D HSQC elucidation substance n°4


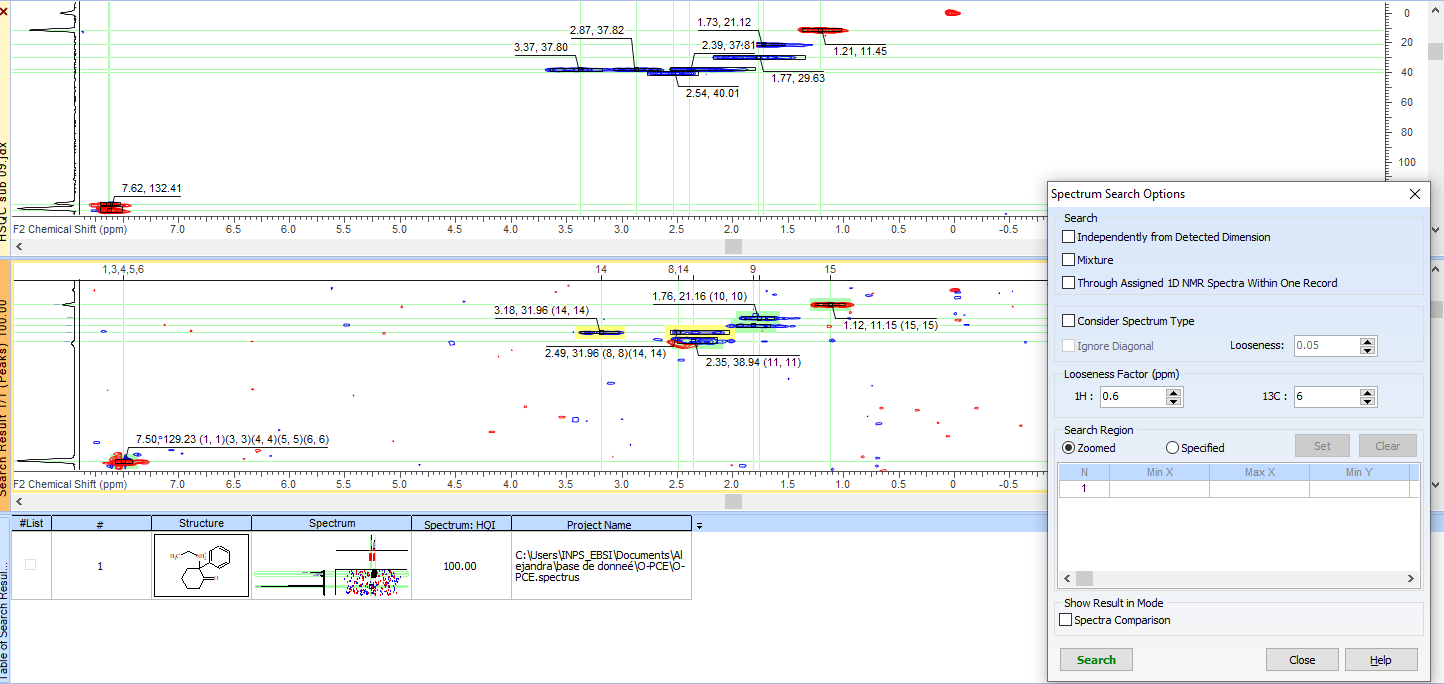


S9: 1D ^1^H substance n°4


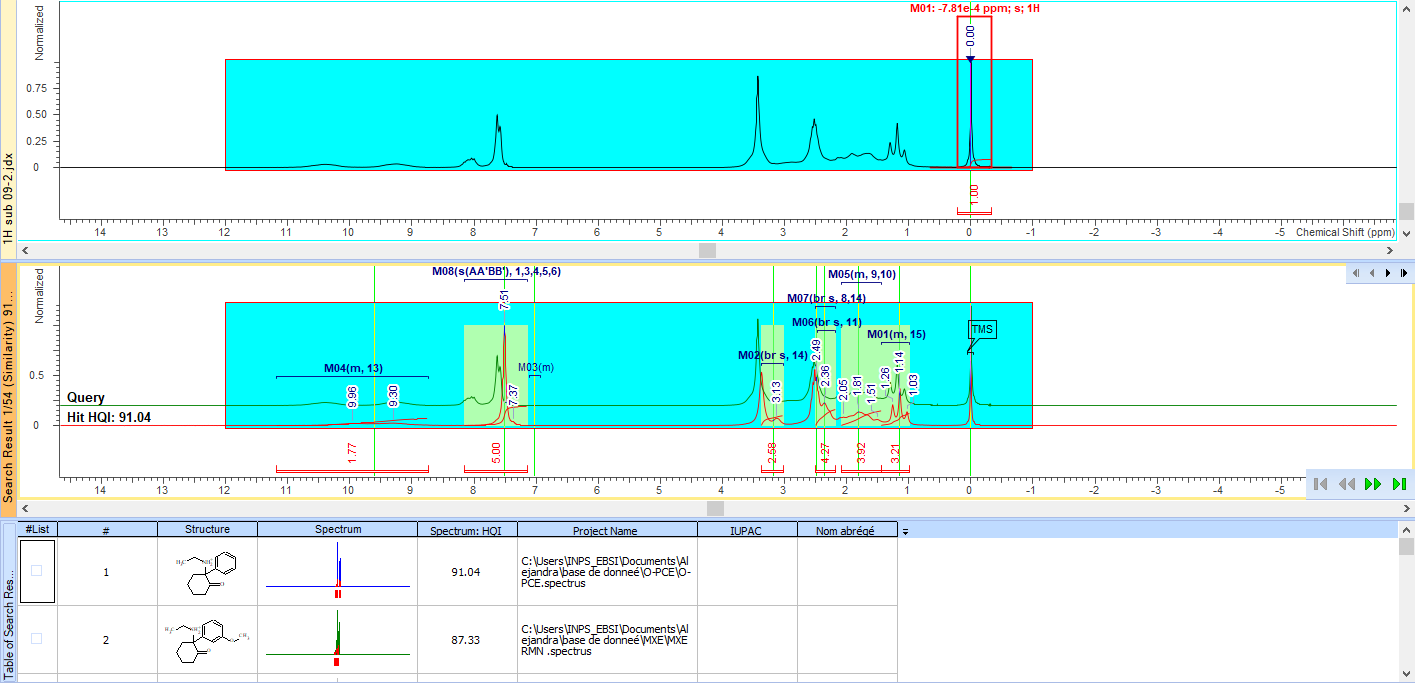


S9: IR substance n°4


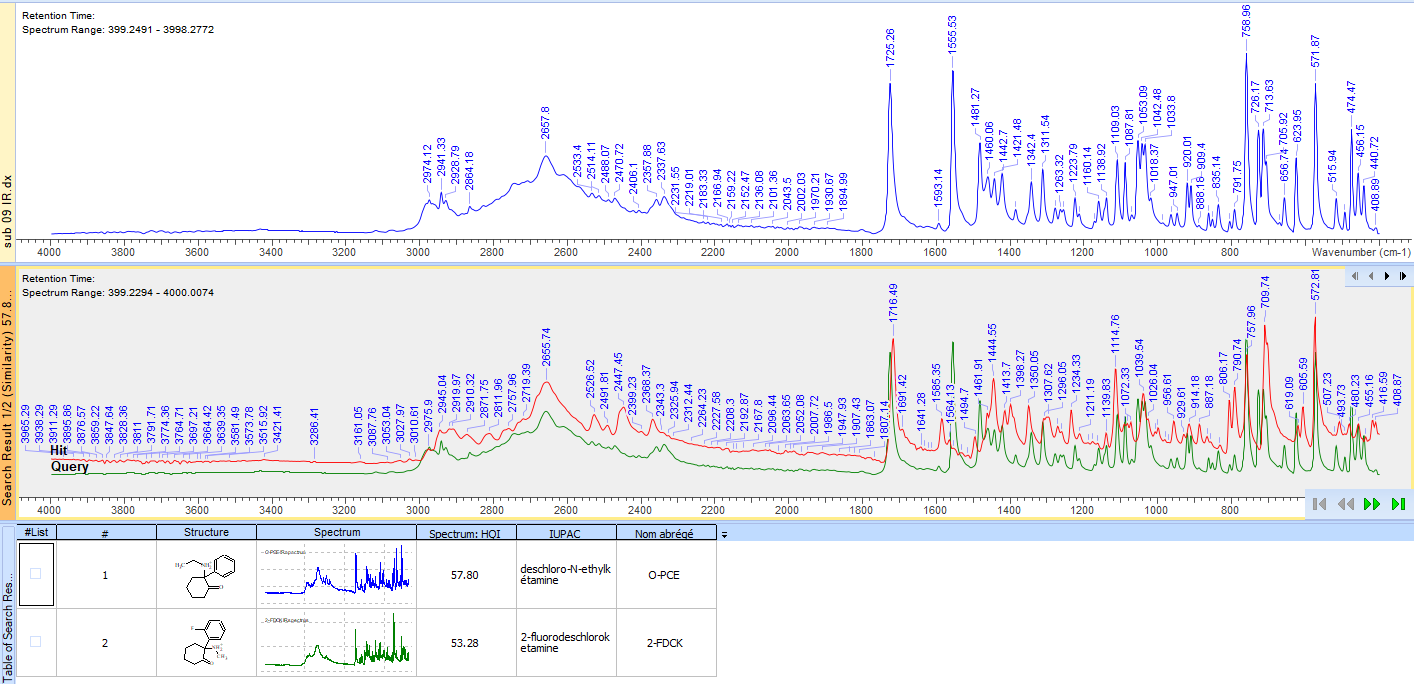


S9: Elucidation substance n°4 at 700MHz

1D ^1^H


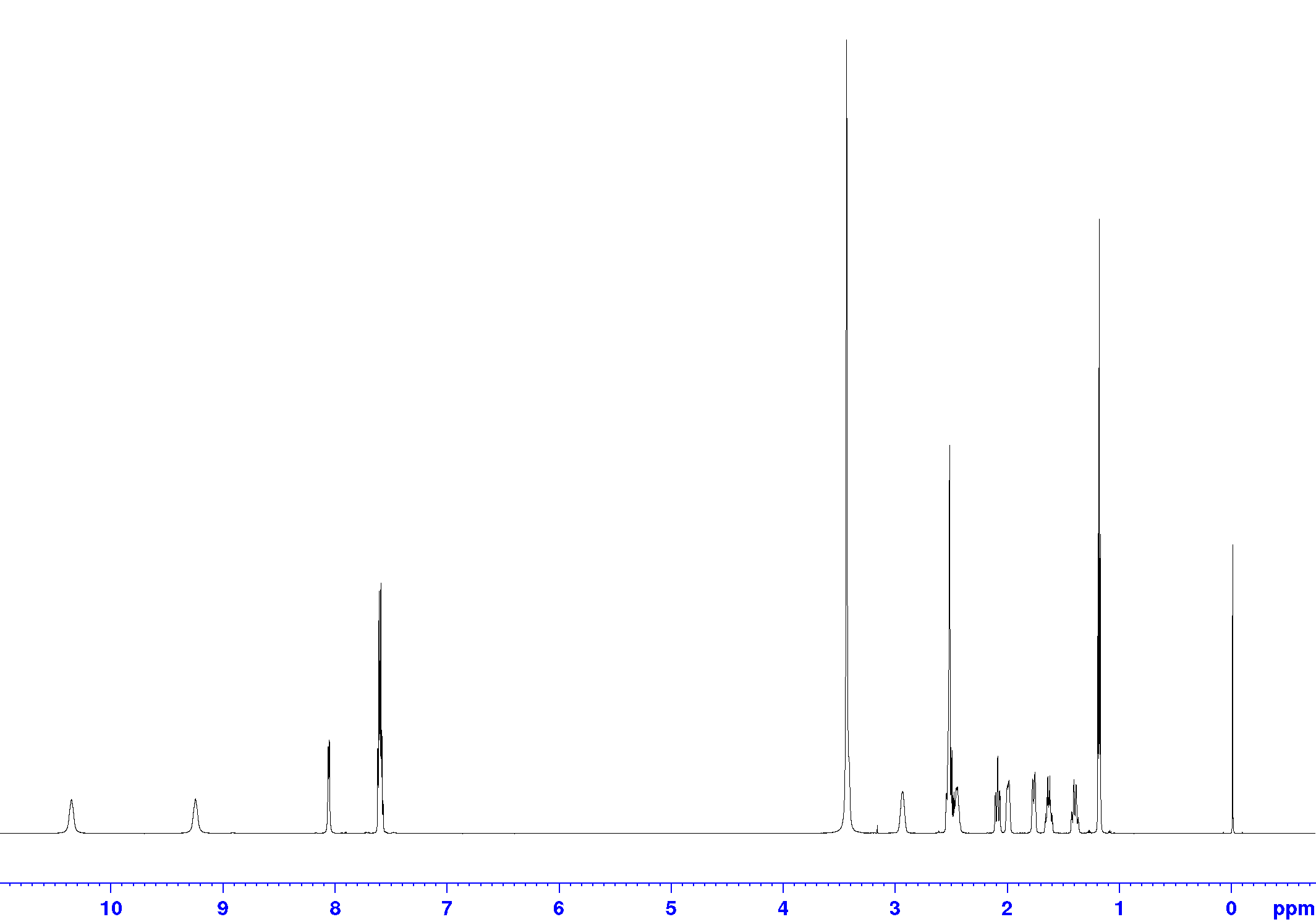


^1^H-^1^H COSY


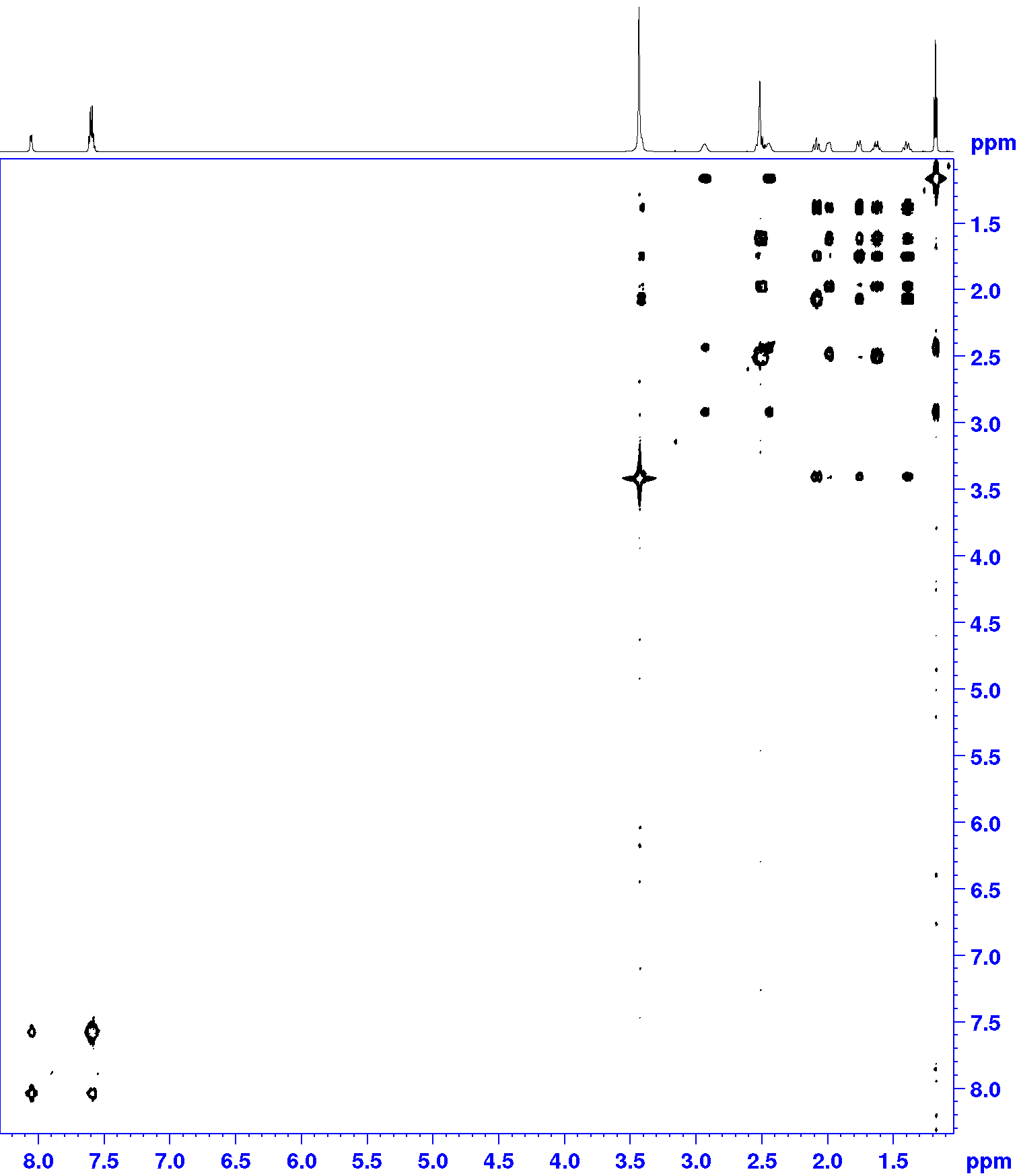


^1^H-^13^C HSQC


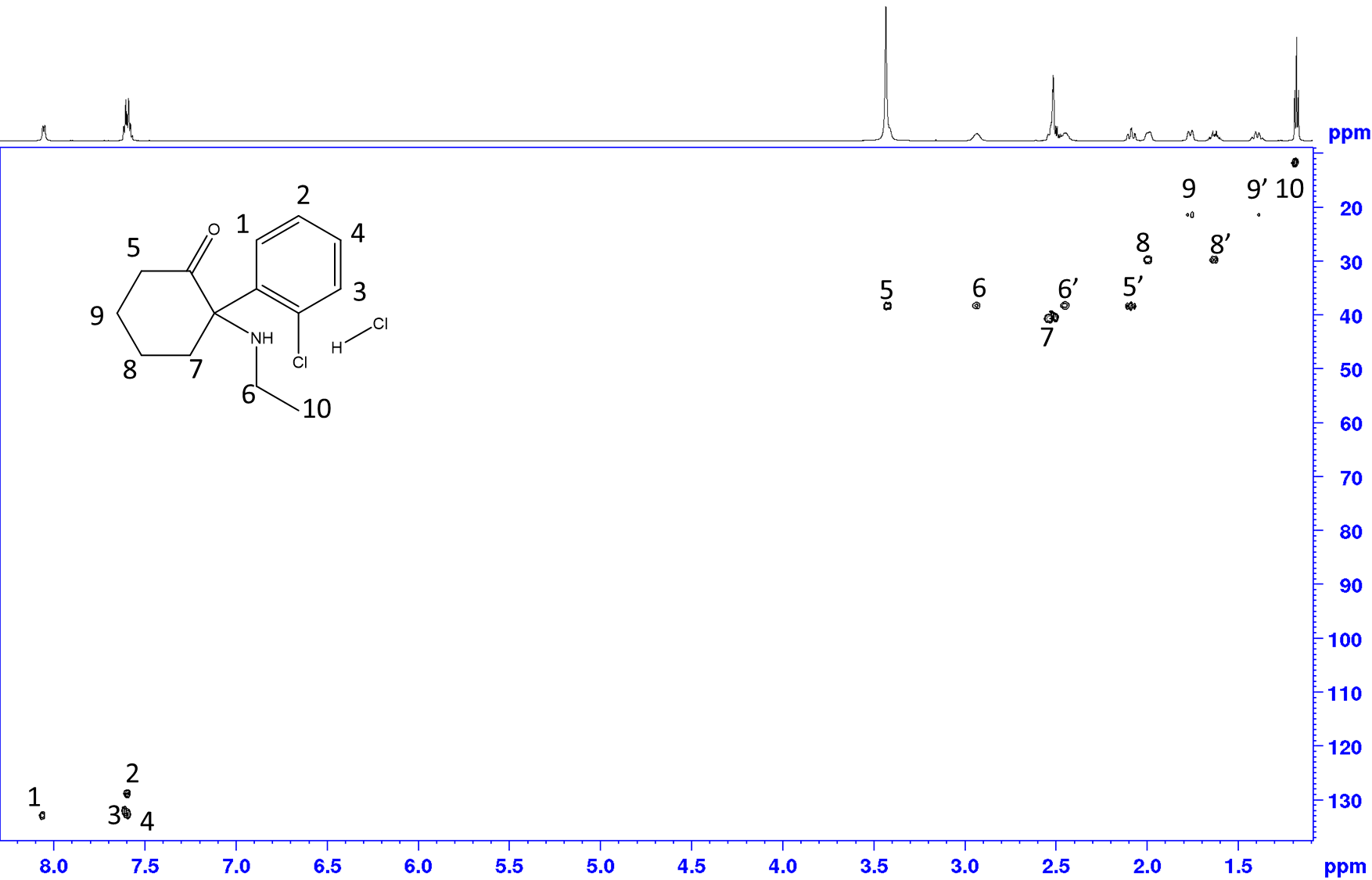


S10: 2D HSQC identification substance n°5


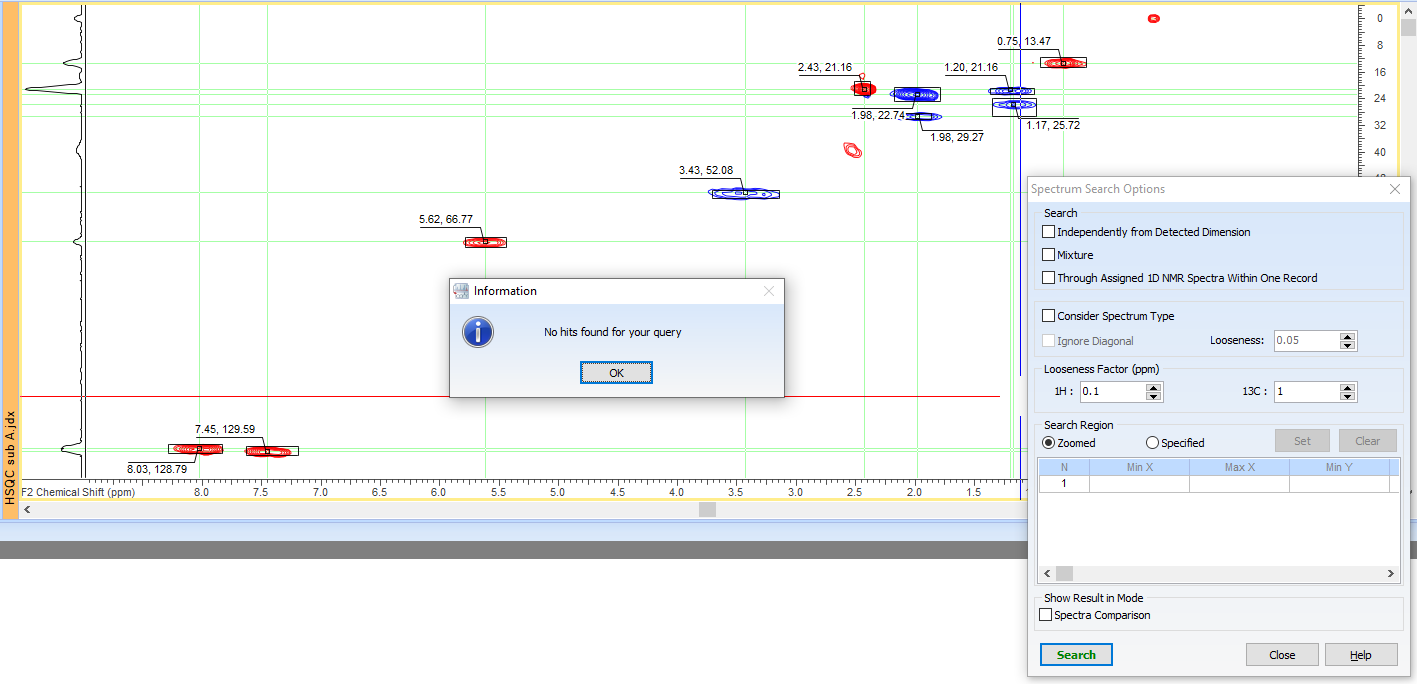


S10: 2D HSQC elucidation substance n°5


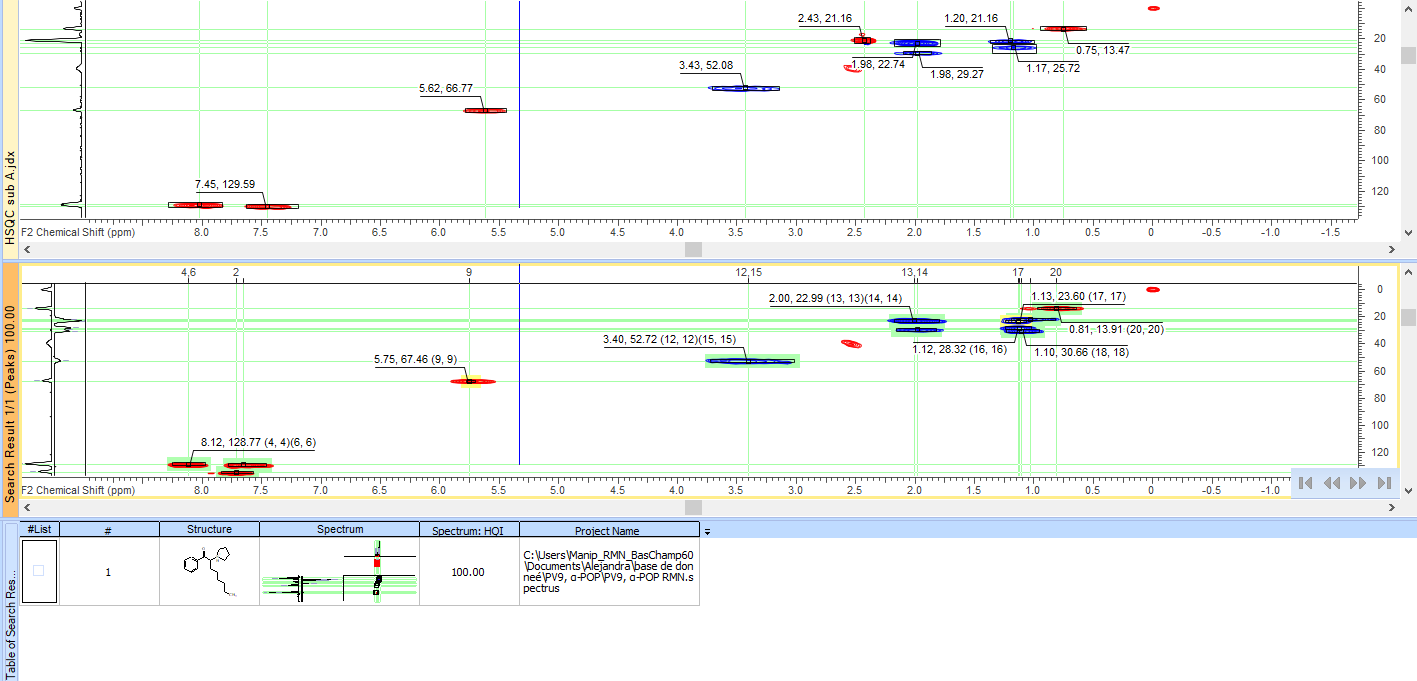


S10: 1D ^1^H substance n°5


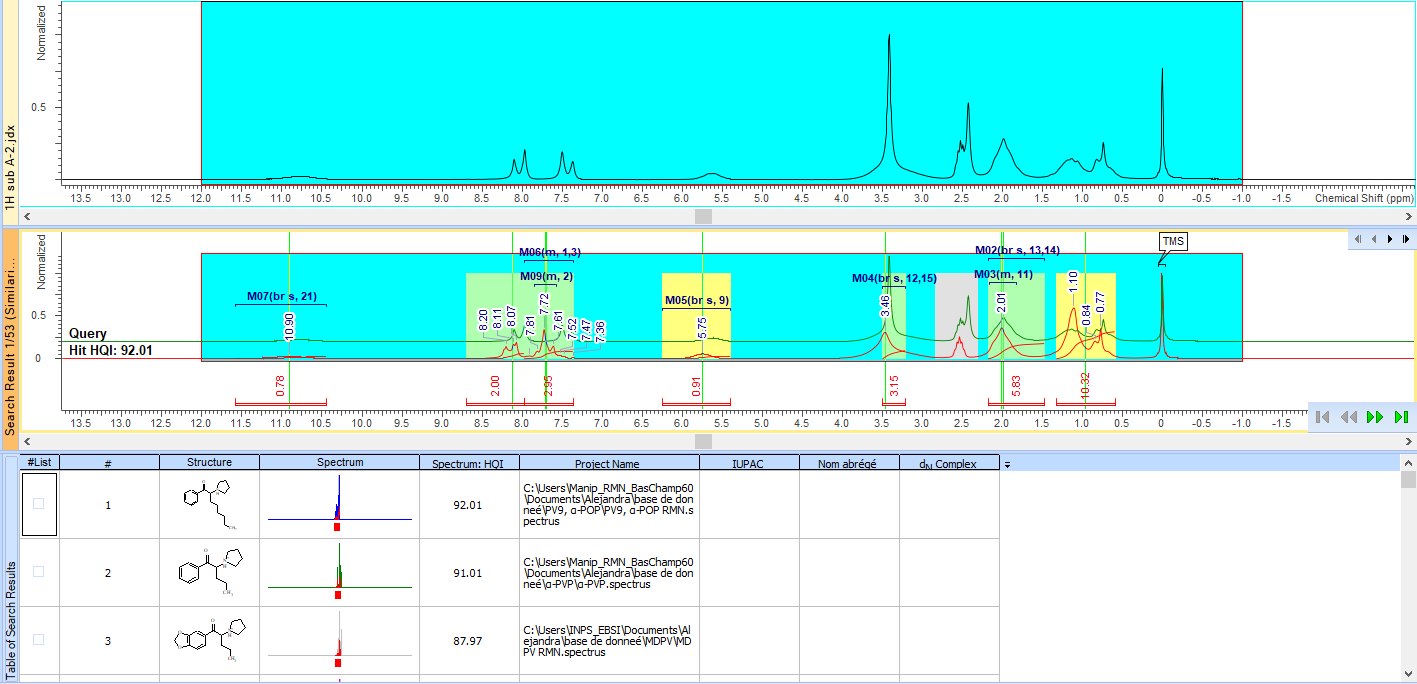


S10: IR substance n°5


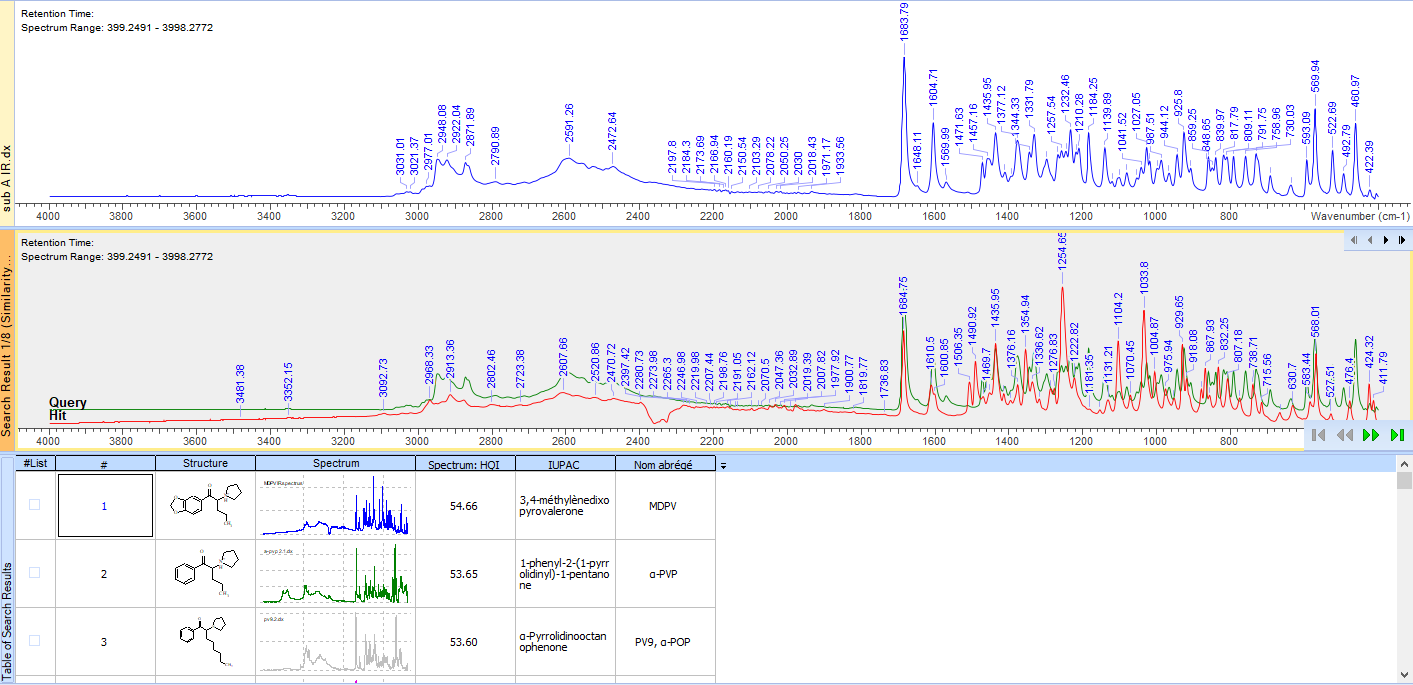


S10: HSQC prediction for MPHP


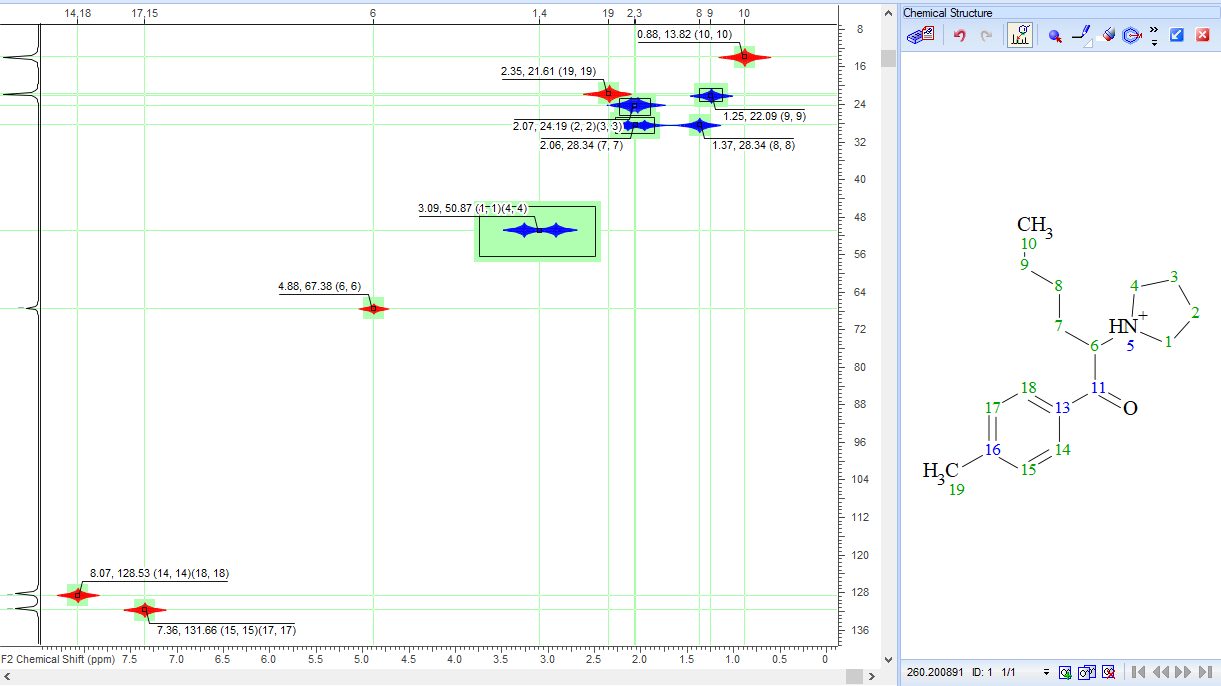


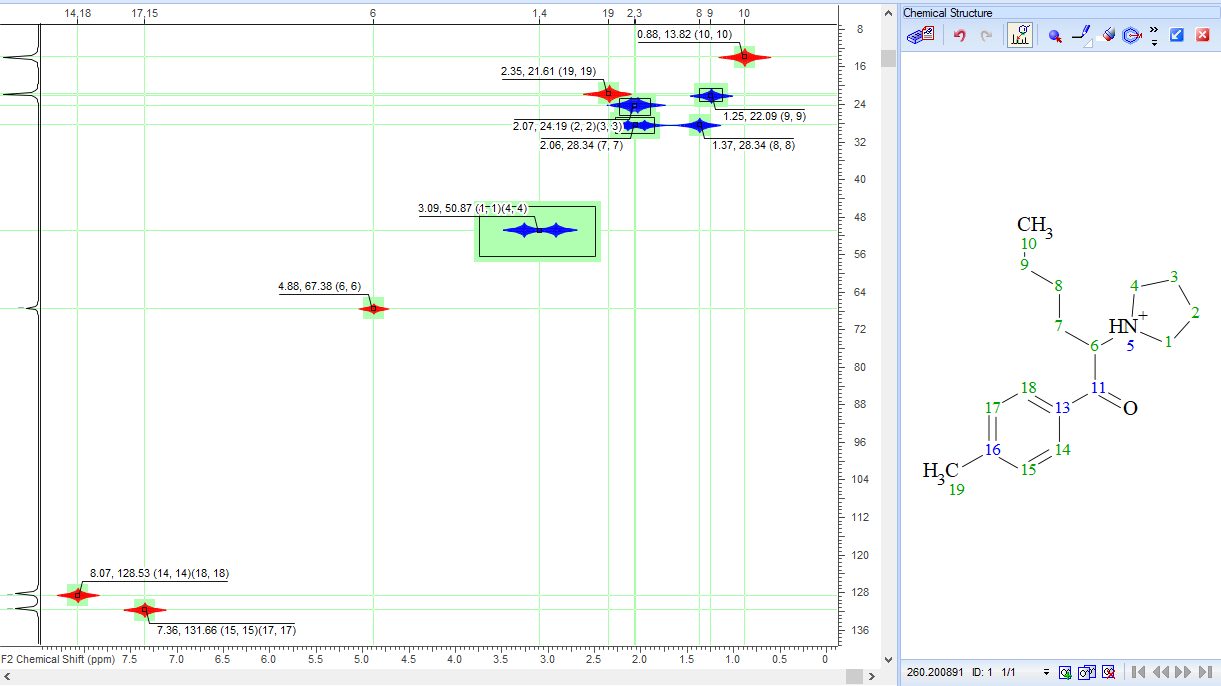


S10A: 700 MHz and predicted HSQC comparison


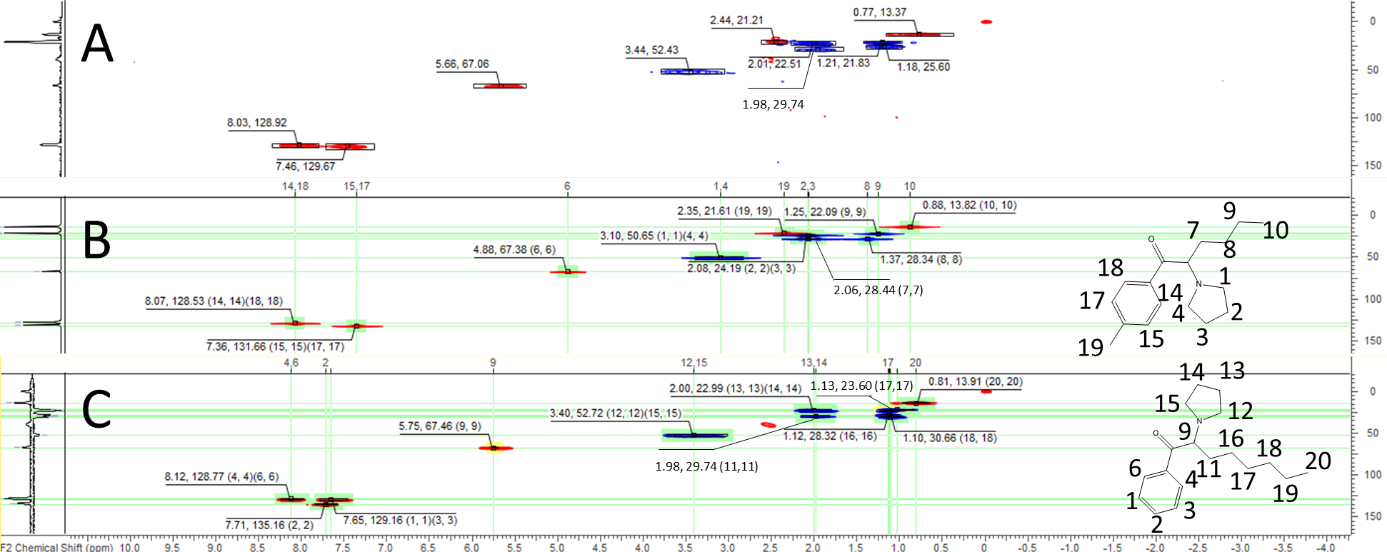


S10: Elucidation substance n°5 at 700MHz

1D ^1^H


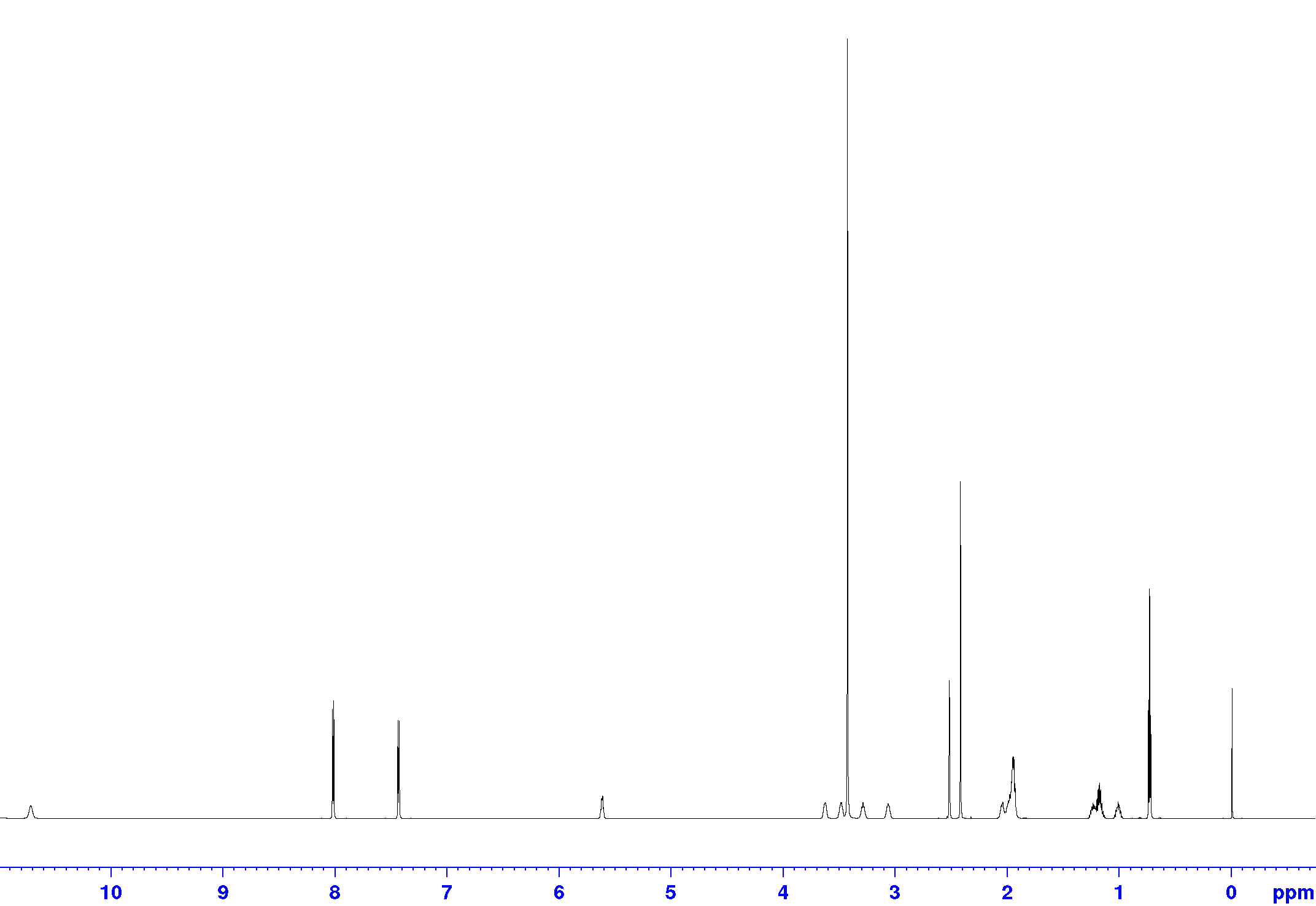


^1^H-^1^H COSY

^1^H-^13^C HSQC

S11: 2D HSQC identification substance n°6

S11: 2D HSQC elucidation substance n°6

S11: 1D ^1^H substance n°6

S11: Elucidation substance 6 at 700MHz

1D ^1^H

^1^H-^1^H COSY

^1^H-^13^C HSQC

S12: Integrations area for the determination of purity
